# Central role for fast nociceptors in mechanical nocifensive behavior and sensitization

**DOI:** 10.1038/s41467-026-75948-z

**Published:** 2026-07-25

**Authors:** John Chwen-Yu Chen, Oumie Thorell, Felipe Meira de-Faria, Aikeremu Ahemaiti, Olivia Le Moëne, Lech Kaczmarczyk, Katharina Henriksson, Jonathan Cole, David A. Mahns, Håkan Olausson, Walker S. Jackson, Saad S. Nagi, Malin C. Lagerström, Marcin Szczot, Max Larsson

**Affiliations:** 1 Division of Cell and Neurobiology, Department of Biomedical and Clinical Sciences, Linköping University, Linköping, Sweden; 2 Centre for Social and Affective Neuroscience, Department of Biomedical and Clinical Sciences, Linköping University, Linköping, Sweden; 3 School of Medicine, Western Sydney University, Sydney, NSW Australia; 4 Department of Immunology, Genetics and Pathology, Uppsala University, Uppsala, Sweden; 5 Wallenberg Centre for Molecular Medicine, Department of Biomedical and Clinical Sciences, Linköping University, Linköping, Sweden; 6 Clinical Neurophysiology, University Hospitals Dorset and Bournemouth University, Poole, UK

**Keywords:** Pain, Somatic system

## Abstract

Nociceptors, primary afferent nerve fibers that signal noxious stimuli, are broadly divided into slowly conducting unmyelinated C fibers and fast-conducting myelinated A fibers. Whereas C-nociceptors have been extensively studied, considerably less is known about the function of A-nociceptors. Here we demonstrate, combining genetic targeting of these fibers in mice with observations in human participants, a key involvement of A-nociceptors in mechanical nociceptive withdrawal reflexes and affective pain. In mice, optogenetic stimulation induced rapid and precise withdrawal reflexes as well as place aversion and facial expression changes consistent with pain affect, while inhibition strongly impaired mechanical nociceptive withdrawal reflexes. Prolonged A-nociceptor activation induced mechanical pain hypersensitivity and central sensitization. In a rare individual lacking thickly myelinated Aβ fibers, and in healthy participants during preferential Aβ-fiber nerve block, mechanical withdrawal reflexes were absent and mechanical pain perception reduced. Together, these findings identify fast-conducting mechano-nociceptors as essential drivers of nocifensive behaviors in mice and humans.

## Abstract (web summary)

The role of fast A-nociceptors in mechanical pain and protective reflexes is unclear. This study shows that these fibers mediate rapid reflexes and contribute to pain perception in humans and mice, and that prolonged activation induces sensitization.

## Introduction

Nociceptors are primary sensory neurons specialized to detect stimuli that damage or threaten to damage tissue integrity. Mammalian nociceptors were initially generally considered to be slowly conducting, unmyelinated C fibers, until Burgess and Perl’s unequivocal observation of faster-conducting, myelinated A fiber nociceptors (A-nociceptors) responsive to high-intensity mechanical skin stimulation in the cat^1^. Since then, electrophysiological studies have identified both mechano-selective, mechano-cold and mechano-heat responsive populations of A-nociceptor in several mammalian species^2–7^, while single axon reconstructions have provided insight into the anatomy of A-nociceptor terminations in the spinal cord^8–10^. More recently, transcriptomic and other studies utilizing genetic techniques have identified gene markers delineating certain A-nociceptor populations^11–18^.

However, the roles of A-nociceptors in pain and nocifensive behavior, and how they provide input to central sensory pathways, remain poorly understood. For instance, while different populations of C-nociceptors are implicated in nociceptive withdrawal reflexes (NWRs) as well as in affective pain and coping behavior^19–25^, A-nociceptor contribution to these processes is less studied^14,16,26^. This can partly be attributed to a dearth of tools for efficient functional manipulation and interrogation of A-nociceptors in animal models. In this study we sought to address this issue by developing a intersectional route for genetic targeting of A-nociceptors as a broad population, taking advantage of the unique co-expression of the neurofilament heavy chain (NFH) and the voltage-gated Na^+^ channel Na_V_1.8 in myelinated nociceptors in the mouse^15,27–30^. We chose to target a broad population of A-nociceptors to uncover a comprehensive picture of the integrated functional role of these fibers, rather than the more subtle roles afforded by smaller subpopulations.

Here, we show that our intersectional targeting approach allows for highly efficient and selective targeting of mechanically activated A-nociceptors, and confirm that such fibers conduct at Aδ to Aβ velocities. We find that these fibers are fundamental for rapid mechanical NWRs but also play a substantial role in affective pain. This is further supported by observations in a rare human Aβ-deafferented individual as well as in healthy individuals subjected to peripheral nerve block of Aβ fibers, who fail to exhibit mechanically evoked NWRs and experience reduced affective pain. Prolonged optogenetic stimulation of murine A-nociceptors induces mechanical allodynia as well as both peripheral and central sensitization, revealing a new route by which pain hypersensitivity can be induced.

## Results

### NFH^CreERT2^;Na_V_1.8^FlpO^ mice enable highly selective targeting of A fiber mechano-nociceptors (A-MNs)

**Fig. 1:**
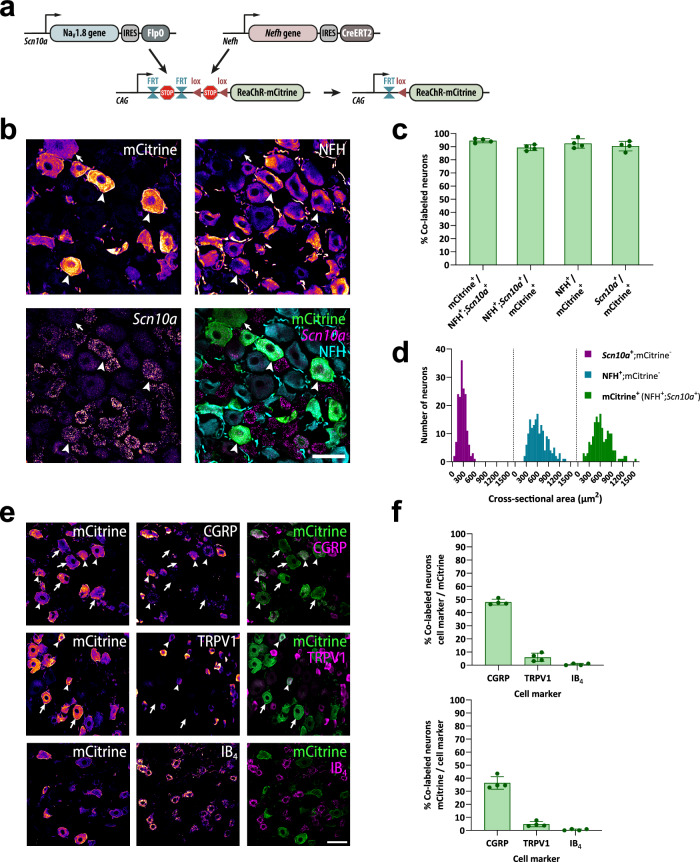
Verification of the NFH;Na_V_1.8;ReaChR mouse line. **a** Schematic of the intersectional genetic targeting approach. **b** Co-localization of mCitrine immunoreactivity (IR) with *Scn10a* transcript detected using in situ hybridization and NFH immunofluorescence in a lumbar dorsal root ganglion (DRG). Single optical section, 20×/0.8 objective. mCitrine, *Scn10a*, and NFH panels are pseudo-colored using the Fire look-up table in Fiji to enhance visibility. Examples of co-localized cells are indicated by arrowheads, while arrows show examples of mCitrine-IR cells with low levels of *Scn10a* transcript. Scale bar, 50 µm. **c** Recombination efficiency with respect to *Scn10a*^+^ cells (mean ± S.D.) in L3-L5 DRGs. Each data point indicates DRG sections from a single mouse; *n* = 4 mice. **d** Size histogram of mCitrine^+^, *Scn10a*^+^;mCitrine^−^, and NFH^+^;mCitrine^−^ DRG neurons. *n* = 4 mice (3 ganglia per mouse). **e** Co-localization of mCitrine-IR with CGRP-IR, IB4 binding, and TRPV1-IR in lumbar DRGs. Arrowheads indicate examples of co-localized cells, whereas arrows indicate examples of mCitrine^+^ cells that do not co-localize with CGRP or TRPV1 immunoreactivity. Single optical section, 20×/0.8 objective. Scale bar, 50 µm, valid for all panels. **f** Quantification of the co-localization of mCitrine with the indicated cell markers. Error bars indicate mean ± S.D. *n* = 4 mice (3 ganglia per mouse). Source data are provided as a Source Data file.

To target a broad population of A-nociceptors in a specific and selective manner, we took advantage of two mouse lines recently developed by us^31,32^: an NFH^CreERT2^ mouse line expressing tamoxifen-dependent Cre recombinase in cells expressing neurofilament heavy chain (NFH), which in the dorsal root ganglion (DRG) is selectively expressed in myelinated neurons; and a Na_V_1.8^FlpO^ mouse line, which selectively targets FlpO recombinase to neurons expressing Na_V_1.8 (encoded by the *Scn10a* gene), a voltage-gated Na^+^ channel selectively expressed in nociceptors and low-threshold C fiber mechanoreceptors. We reasoned that crossing these mouse lines would direct Cre/Flp-dependent reporter expression specifically to DRG neurons expressing both NFH and Na_V_1.8 (Fig. 1a), and that this targeted group of neurons largely would comprise A-nociceptors. A triple cross of these mouse lines with a mouse line expressing, in a Cre/Flp-dependent manner, the red-activated channelrhodopsin ReaChR^33^ fused to the yellow fluorescent protein mCitrine resulted in membrane-targeted mCitrine expression in numerous DRG neurons. In L3–5 DRGs of these mice (here termed NFH;Na_V_1.8;ReaChR), 94.5 ± 1.6% (mean ± S.D.; *n* = 4 mice) of neurons exhibiting both NFH immunoreactivity and *Scn10a* mRNA were mCitrine^+^; 92.5 ± 3.6% showed NFH immunoreactivity and 90.5 ± 3.6% exhibited *Scn10a* mRNA signal, while 89.3 ± 2.2% of mCitrine^+^ cells were both NFH^+^ and *Scn10a*^+^ (Fig. 1b, c). Thus, this intersectional strategy resulted in targeting of NFH^+^/Na_V_1.8^+^ neurons with high specificity and selectivity. mCitrine^+^ DRG neurons were medium-to-large sized, markedly different from the size distribution of neurons that were *Scn10a*^*+*^ only, but similar to that of NFH^+^ only neurons (Fig. 1d). Notably, only 48.0 ± 2.2% (*n* = 4 mice) of mCitrine^+^ neurons in L3–5 DRGs showed detectable immunoreactivity for calcitonin gene-related peptide (CGRP), a marker of peptidergic nociceptors; conversely, 36.4 ± 4.8% of CGRP^+^ neurons were mCitrine^+^ (Fig. 1e, f). Few mCitrine^+^ neurons exhibited TRPV1 immunoreactivity (6.0 ± 3.1%), while essentially no co-localization was observed with binding sites for isolectin B_4_ (IB_4_), a marker of non-peptidergic C fibers.

**Fig. 2:**
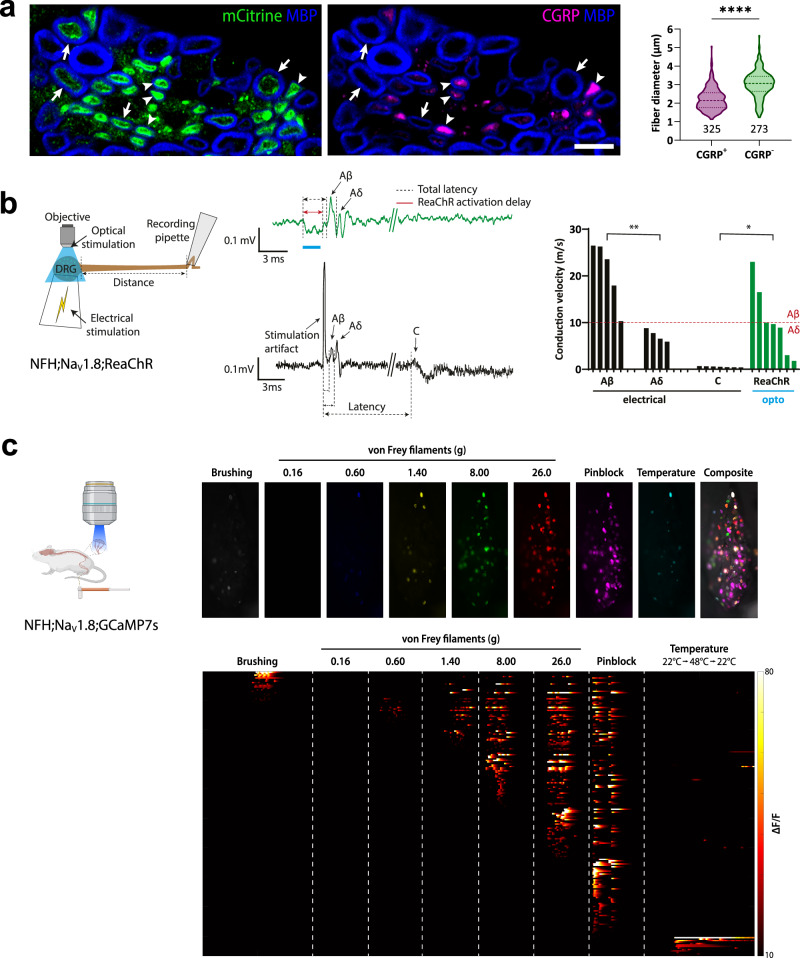
Fiber classes of targeted neurons in NFH;Na_V_1.8;ReaChR mice. **a** Left panels show example micrograph of sciatic nerve sections immunolabelled for mCitrine and CGRP. Single optical section, 63×/1.4 objective. Examples of mCitrine^+^ fibers without CGRP-IR are indicated by arrows, and mCitrine^+^ fibers colocalized with CGRP-IR are indicated by arrowheads. Scale bar, 5 µm. The right panel shows violin plots of fiber diameters (including MBP^+^ myelin sheath) of CGRP^+^ and CGRP^−^ mCitrine^+^ fibers in the sciatic nerve. *n* = 3 mice (1 sciatic nerve section per mouse). The number beneath each violin plot indicates the number of fibers analyzed. ****, *p* < 0.0001, Kolmogorov–Smirnov test. Statistics details are available in Supplementary Data 1. **b** Conduction velocity (CV) of optogenetically evoked compound action potentials (CAPs). The left panel shows a schematic of the DRG-dorsal root ex vivo preparation used. The measured distance was used for determining the CV. Middle panels, example traces of optogenetically (top) or electrically (bottom) evoked recordings. Blue line indicates time of optogenetic activation; arrow in top trace indicates optogenetic response. The dashed arrow indicates the stimulation to signal (total) latency, while the red arrow indicates the delay for ReaChR activation, which was subtracted from the total latency to yield the conduction latency. Right panel, a summary plot of the CVs of CAPs evoked by electrical (black) or optogenetic stimulation (green). The red dashed line indicates the border between Aδ and Aβ fiber CVs (Djouhri et al.^35^). **c** In vivo Ca^2+^ imaging of NFH^+^/Na_V_1.8^+^ DRG neurons. NFH;Na_V_1.8;GCaMP7s mice were subjected to in vivo imaging of L4 DRGs to assess responses to natural stimuli applied to the skin of the plantar hind paw. The top panels show example micrographs of responses to different stimuli in an L4 DRG. Bottom panel, a heat map of the responses of GCaMP7s^+^ cells to cutaneous stimuli as indicated. Each row indicates the responses to the applied stimuli of an individual cell. *n* = 193 cells, collected from three L4 DRGs (one ganglion per mouse). Schematic created in BioRender. Larsson, M. (2026) https://BioRender.com/yim1dhh. Source data are provided as a Source Data file.

Given the medium-to-large size of mCitrine^+^ neurons in the NFH;Na_V_1.8;ReaChR mice and their expected identity as myelinated nociceptors, we decided to examine the size of the peripheral fibers arising from the targeted neurons in transverse sciatic nerve sections. As some but not all of the neurons were found to express CGRP, we also co-immunolabeled the sections for this neuropeptide. Both CGRP^+^ and CGRP^−^ populations of mCitrine^+^ fibers were found. mCitrine^+^ fibers that were CGRP^+^ were of significantly smaller diameter (including MBP^+^ myelin sheath; median 2.1 µm, interquartile range 1.8–2.6 µm) than those that lacked detectable CGRP (median 3.1 µm, interquartile range 2.6–3.5 µm; *P* < 0.0001, Kolmogorov–Smirnov test) (Fig. 2a). This indicates a clear distinction with respect to conduction velocity (CV) between peptidergic and non-peptidergic (or peptide-poor) A-MNs. To determine CVs of targeted fibers in the NFH;Na_V_1.8;ReaChR mice, we employed an ex vivo optogenetics/electrophysiology approach, where optogenetically induced compound action potentials (CAP) were recorded in DRG-dorsal root preparations. First, reference measurements were conducted using electrical dorsal root stimulations. Incrementally increasing electrical stimulations (0–2 mA, 0.1 ms) to the DRG progressively activated Aβ, Aδ, and C fibers. The fastest CAP was considered to be the Aβ component, followed by the Aδ and C fiber components^34^. The CV of Aβ fibers ranged from 10.3 to 26.3 m/s with an average value of 20.9 ± 2.7 m/s (Fig. 2b). Aδ fibers exhibited CVs between 6.6 and 8.8 m/s, averaging at 7.3 ± 0.6 m/s, and C fibers ranged from 0.42 to 0.68 m/s, with an average value of 0.54 ± 0.04 m/s. Based on our findings and previous reports^3,35^, fibers with CVs slower than 1.2 m/s were classified as C fibers, those ranging from 1.2 to 10 m/s were categorized as Aδ fibers, and Aβ fibers were defined by CVs greater than 10 m/s. Unlike electrical stimulation, which instantly delivers activation currents to target cells, optogenetic stimulation introduces an activation delay due to the kinetics of ReaChR^33^. To account for this, CV values of ReaChR-activated fibers were corrected by subtracting the average spike delay of ReaChR (2.27 ms, *n* = 6) measured via cell-attached current-clamp recordings (Fig. S1). The corrected CV values (*n* = 5) ranged from 1.81 to 22.97 m/s, indicating that optogenetic stimulation of ReaChR activated Aβ and Aδ fibers, but not C fibers (Fig. 2b). Among all ReaChR stimulations, two recordings showed activation of both Aβ and Aδ fibers, while in the remaining recordings, only Aδ fiber activation was detected. Based on the optogenetic/electrophysiological data and the morphological observations, we propose that a proportion of CGRP-lacking A-MNs are likely to conduct at Aβ fiber velocities in the mouse.

To probe the response characteristics of NFH^+^/Na_V_1.8^+^ fibers to natural stimuli, we performed in vivo Ca^2+^ imaging in lumbar (L4) DRGs of NFH;Na_V_1.8;GCaMP7s mice, which expressed GCaMP7s in a Cre/Flp-dependent manner. Very few cells responded to gentle brushing or thermal stimuli applied to the plantar hind paw, whereas the vast majority of cells responded to a pin prick applied to the same region of the skin (Fig. 2c). Mechanical thresholds were determined manually using von Frey filaments. Most responsive cells had a moderate-to-high mechanical threshold; no cells were activated by a 0.16 g filament, whereas 9% (17/193) of cells responded to 0.60 g, and 15% responded to 1.40 g but not to the 0.60 g filament. Although some LTMRs may show thresholds above 0.16 g (corresponding to ~1.6 mN)^3,5,11^, it is likely that most cells responding first to 0.60 g or 1.40 g were moderate pressure receptors (MPRs), a subclass of A-nociceptors with relatively low mechanical thresholds capable of encoding the intensity of mechanical forces in the noxious range^1,8,36^. About 45% of cells had a very high threshold, with 20% responding to 26.0 g but not 8.00 g or lower force filaments, and around 25% of cells responded only to a pin prick. As few cells responded to a temperature ramp from 22 to 48 °C, we conducted a separate set of experiments to more comprehensively assess thermal responses (Fig. S2). Here, a pin prick was used to identify mechanical responsivity, after which a cold ramp (34 °C → 10 °C → 34 °C) and lastly a heat ramp (34 °C → 55 °C → 34 °C) were applied to the plantar hind paw. As in the first set of experiments, few neurons were thermoreceptive; only 4% (7/170) of mechanoreceptive cells responded to either cold or heat, whereas 5% (9/179) of all cells were thermo but not mechanoresponsive. Note that because some mechano-cold A-nociceptors in the mouse have thermal thresholds below 10 °C^5^, a portion of neurons classified as pure mechanoreceptors in our experiments may have been mechano-cold receptors. To conclude, the population of DRG neurons targeted in these mice largely consisted of A-MNs, a small proportion of which were also thermoreceptive.

### Cutaneous A-MNs form free nerve endings and circumferential hair follicle endings

**Fig. 3:**
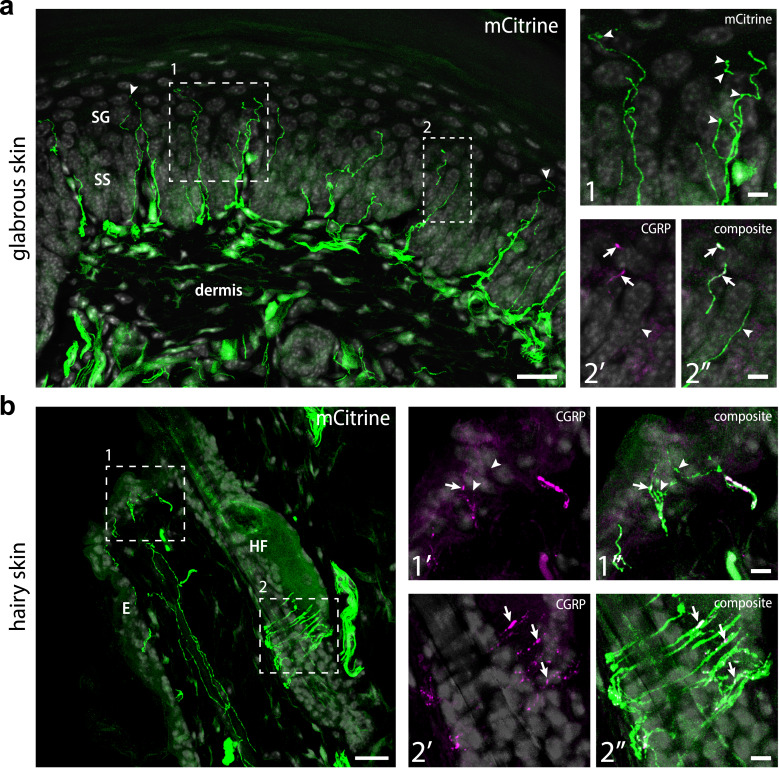
Cutaneous termination of NFH^+^/Na_V_1.8^+^ fibers. **a** Example micrograph of hind paw glabrous skin from an NFH;Na_V_1.8;ReaChR mouse showing mCitrine^+^ fibers forming free nerve endings in the epidermis. DAPI nuclear staining is shown in grey to visualize layers of the skin. Arrowheads indicate examples of mCitrine^+^ fibers extending through stratum spinosum (SS) deep into stratum granulosum (SG). Dashed regions in the left panel are shown magnified as numbered in panels to the right. Arrowhead in (1) indicates example varicosities in mCitrine^+^ fibers. 2’, 2” Show CGRP-IR and mCitrine/CGRP-IR, respectively. Arrows indicate an mCitrine^+^/CGRP^+^ fiber, while arrowheads indicate an mCitrine^+^/CGRP^−^ fiber. Scale bar in left panel, 20 µm; scale bar in right panels, 5 µm. Micrographs are maximum intensity projections of 18 deconvolved optical sections obtained at 0.57 µm separation using a 25×/0.95 objective. **b** Example micrograph from the hairy back skin of an NFH;Na_V_1.8;ReaChR mouse, showing a circumferential nerve ending around a hair follicle (HF) and fibers forming free nerve endings in the adjacent epidermis (E). DAPI staining is shown in grey. Dashed numbered regions are shown magnified in the panels to the right. Panels 1’, 1” show epidermal mCitrine^+^ free nerve endings positive (arrow) or negative (arrowheads) for CGRP. Panels 2’, 2” show the hair follicle circumferential nerve ending co-localizing with CGRP-IR. Scale bar in left panel is 20 µm; scale bar in right panels is 5 µm. Maximum intensity projection of 20 deconvolved optical sections acquired at 0.57 µm separation using a 25×/0.95 objective. Micrographs are representative of independent experiments from four mice.

A-nociceptors were long posited to form free nerve endings in the skin^37^, but direct evidence has been scarce; indeed, at least one type of A-MN forms circumferential nerve endings around hair follicles^11,13,14^. To gain an overview of the spectrum of cutaneous nerve ending morphologies formed by A-MNs, we assessed the skin innervation of mCitrine^+^ fibers in NFH;Na_V_1.8;ReaChR mice. In glabrous hind paw skin, numerous mCitrine^+^ fibers emanated from subepidermal plexa into the epidermis, often extending deep into stratum granulosum (Fig. 3). Some of these fibers formed varicosities along their path through the epidermis, and sometimes, where these could be followed to their distal end, also formed an end bouton. No association with Meissner corpuscle-like structures was found (Fig. S3). Some fibers were CGRP^+^, often weakly so, but many were not. In hairy back skin, epidermal CGRP^+^ and CGRP^−^ free nerve endings were found, but also circumferential endings around hair follicles. The circumferential endings around hair follicles were CGRP^+^ and likely corresponded to the previously characterized hair pull nociceptors^13,14^. Apart from epidermal free nerve endings and circumferential nerve endings, no other type of specialized nerve ending could be distinguished.

### A-MNs terminate throughout most of the spinal dorsal horn in a segmentally specific manner

**Fig. 4:**
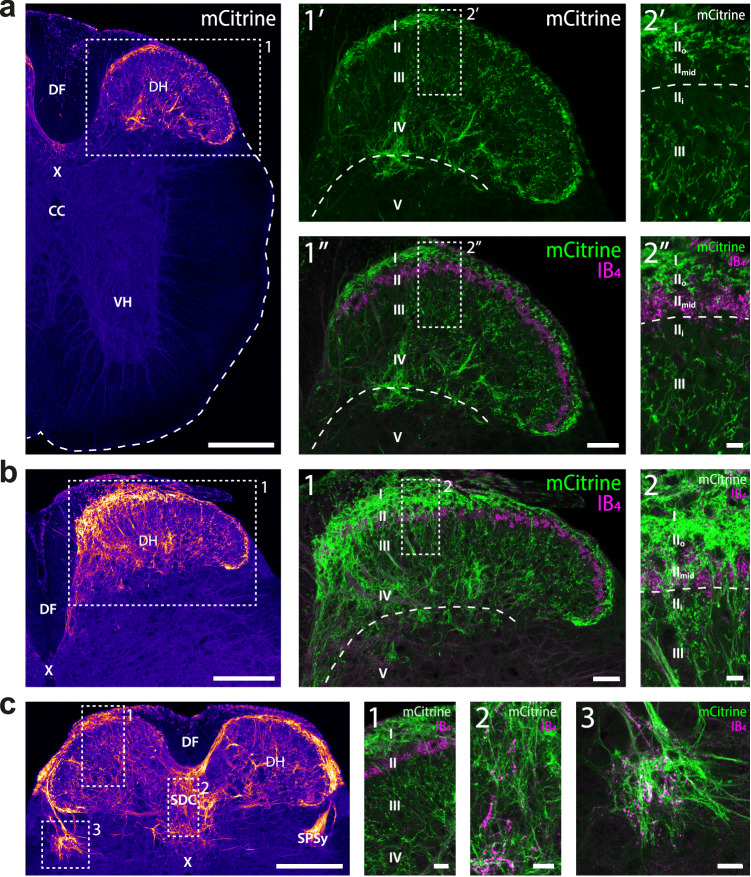
Spinal cord termination of NFH^+^/Na_V_1.8^+^ fibers. Transverse spinal cord sections from NFH;Na_V_1.8;ReaChR mice showing native mCitrine fluorescence. **a** Thoracic spinal cord. The left panel is pseudo-colored using the Fire look-up table in Fiji to enhance visibility of weak fluorescence. mCitrine fluorescence is restricted to the dorsal horn. The dashed region is magnified in 1’, showing mCitrine fluorescence, and 1”, showing mCitrine with IB_4_ binding (magenta) to outline the middle third of lamina II. Dashed lines show the approximate dorsal border of lamina V. Note the sparsity of mCitrine^+^ fibers in lamina V except the dorsal part, and the comparative density of fibers in lamina IV. Dashed region in (1’, 1”) is further magnified in the rightmost panels. Dashed line indicates the ventral border of mid-lamina II (II_mid_), as defined by IB_4_. Note the dense plexus of mCitrine^+^ fibers in lamina I and outer lamina II (II_o_), the sparseness of fibers in II_mid_ and inner lamina II (II_i_), and the intermediate density of fibers in lamina III. Scale bar in left panel, 200 µm; scale bar in center panels, 50 µm; scale bar in right panels, 10 µm. **b** L4 spinal cord. Dashed frame indicates region magnified in center panel (1) showing mCitrine fluorescence and IB_4_ binding. The dashed line indicates the border between laminae IV and V. The dashed region in the center panel is further magnified in (2). Note the higher fiber density in lamina II_mid_ and II_i_ as compared to the lateral parts of the same section, and to the thoracic spinal cord in (**a**) Scale bars are as in (**a**). **c** S1 spinal cord. The sacral dorsal commissural nucleus (SDC) and sacral parasympathetic nucleus (SP) are densely innervated. Dashed frames indicate numbered insets with mCitrine fluorescence and IB_4_ labeling. Scale bar in left panel, 200 µm; scale bars in insets, 20 µm. DF dorsal funiculus, DH dorsal horn, VH ventral horn, CC central canal; Roman numerals indicate Rexed’s laminae. All micrographs are maximum intensity projections of 5 optical sections acquired at 1.04 µm separation with a 20×/0.75 objective. The micrographs are representative of experiments repeated in at least four mice.

The superficial dorsal horn (Rexed’s laminae I and dorsal lamina II) is considered the major target site of nociceptive primary afferent fibers and a core site of nociceptive signal processing^38^. By contrast, the deeper dorsal horn, including the ventralmost lamina II to V, is generally thought to mainly receive terminations from low-threshold mechanoreceptors (LTMRs)^38–41^. Notably, although this region also contains neurons that receive nociceptive input, such input is thought to primarily occur at synapses onto these neurons’ dorsally directed dendrites in the superficial dorsal horn, or via polysynaptic routes^38,40^. However, morphological reconstructions of intracellularly recorded afferent fibers in mice have suggested that some myelinated A-nociceptors arborize throughout the dorsal horn, including laminae III–V^8^. To resolve this discrepancy, we examined the terminations of mCitrine^+^ fibers in the spinal cord of NFH;Na_V_1.8;ReaChR mice. Central processes of targeted mCitrine^+^ neurons were distributed throughout much of the dorsal horn (Fig. 4). The densest innervation was found in lamina I, extending into outer lamina II (II_o_), the ventral border of which was defined by the band of IB_4_ binding terminals. In general, few processes were found in the ventral two-thirds of lamina II; however, in the lumbar enlargement, medial parts of lamina II exhibited considerably denser innervation also in mid- and inner lamina II (Fig. 4b). A similar pattern of denser medial innervation was found in the C7–C8 spinal cord that receives input from the fore paw (Fig. S4a). As the medialmost dorsal horn in these spinal segments selectively receives input from the glabrous skin of front and hind paws^42,43^, this pattern suggests that a glabrous skin-specific myelinated nerve fiber population, which uncharacteristically terminates in this region, is among those targeted in NFH;Na_V_1.8;ReaChR mice. To further examine this, we immunolabeled spinal cord sections from C7–8 to L4–5 segments of NFH;Na_V_1.8;ReaChR mice for VGluT3, a marker of rodent C-LTMRs that terminate in inner lamina II^44^. Because this C-LTMR population only innervates hairy skin, lamina II regions with input from glabrous skin are devoid of VGluT3^+^ C-LTMR terminals^32^. In accordance with a glabrous skin-selective origin of the dense A-MN innervation of lamina II, VGLUT3^+^ terminals were excluded from, and lateral to, areas of dense mCitrine^+^ innervation (Fig. S4a). We further injected cholera toxin B subunit conjugated to the fluorophore CF594 (CTB^CF594^) at several sites in the glabrous skin of the right hindpaw. In the L4–5 spinal dorsal horn, regions targeted by fibers labeled by glabrous skin CTB^CF594^ were also sites of dense lamina II innervation by mCitrine^+^ fibers (Fig. S4b), further strengthening the notion that certain A-MN subpopulations innervating glabrous but not hairy skin give rise to central terminations in the ventral two-thirds of lamina II.

Apart from the superficial dorsal horn, mCitrine^+^ processes in NFH;Na_V_1.8;ReaChR mice were detected in lamina III and IV albeit at a lower density than in more superficial laminae (Fig. 4). A few medial lamina IV fibers appeared to cross the midline to the contralateral dorsal horn, while a few processes entered dorsal lamina X. Somewhat surprisingly however, in lamina V substantial innervation was restricted to the most dorsal part of the lamina, whereas more ventrally only occasional fibers were observed. Moreover, mCitrine^+^ fibers were not uniformly distributed along the mediolateral axis in laminae IV–V, as some of them congregated into large plexa. In the sacral spinal cord, in addition to innervation of lamina I and deeper laminae, dense innervation was also observed in the sacral dorsal commissural nucleus (SDC) and in the sacral parasympathetic nucleus (SPN) (Fig. 4c), indicating a large contribution of A-nociceptors to pelvic visceral afferent sensory modalities and to the autonomous regulation of pelvic organs.

**Fig. 5:**
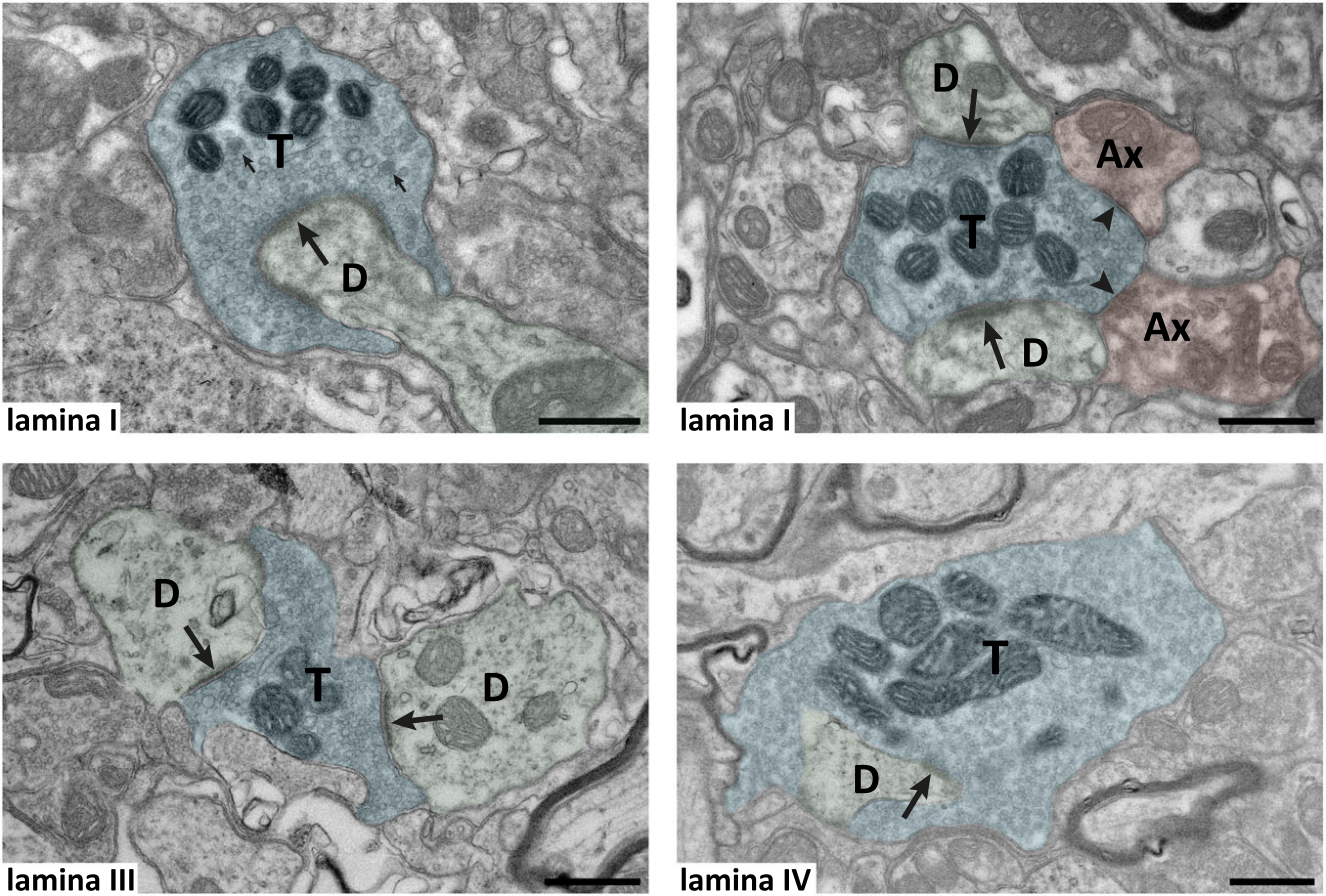
Ultrastructure of spinal termination of NFH^+^/Na_V_1.8^+^ fibers. Shown are example electron micrographs from the lumbar spinal cord of NFH;Na_V_18;APEX2 mice. Mitochondria of APEX2-expressing cells show electron-dense reaction product in the matrix. In all panels, nerve terminals labeled (T) possess mitochondria with APEX2 reaction product that makes those clearly darker in appearance than mitochondria in the surrounding neuropil, thus identifying their parent terminal as originating from NFH^+^/Na_V_1.8^+^ fibers. The top left panel shows an APEX2^+^ terminal in lamina I with a simple dome-shaped appearance forming a single synapse (large arrow) onto a dendrite (D). Small arrows indicate examples of dense core vesicles that presumably contain neuropeptides. Top right panel, an APEX2^+^ terminal in lamina I, that is a central terminal of a synaptic glomerulus, forming two synapses (arrows) onto two dendrites but also receiving synapses (arrowheads) from two presumed inhibitory presynaptic axons (Ax). Bottom left, an APEX2^+^ terminal in lamina III forming a synaptic glomeruli with synapses onto two dendrites. Bottom right, a moderately large APEX2^+^ terminal forming a single obliquely sectioned synapse onto a dendrite. Scale bars in all panels are 500 nm. In all panels, terminals and dendrites are pseudo-colored to increase readability. The micrographs are representative of experiments from three mice.

Primary afferent fibers in the dorsal horn often establish complex synaptic structures known as synaptic glomeruli in the spinal dorsal horn. These glomeruli, in which a central primary afferent terminal makes multiple axodendritic synaptic contacts but also receives inhibitory axo- and dendroaxonic synapses, afford complex signal integration already at the first sensory synapse. However, while these structures are well-established features of C fiber termination^45,46^, the synaptic organization of A-MNs has only been assessed for a small number of physiologically identified fibers in cat and monkey^47^. Moreover, for these fibers, only terminals in laminae I and V were reported. To determine the synaptic organization of mouse A-MNs and confirm that the presence of mCitrine^+^ fibers in laminae III–IV of NFH;Na_V_1.8;ReaChR mice reflects A-MN synapses in this region, we crossed NFH^CreERT2^;Na_V_1.8^FlpO^ mice with an LSL-FSF-APEX2 mouse line, which expresses the peroxidase APEX2 in the mitochondrial matrix in a Cre/Flp-dependent manner. After peroxidase histochemistry, the electron-dense reaction product is readily detected within mitochondria in APEX2-expressing cells by electron microscopy. In laminae I and II_o_, we observed numerous terminals harboring APEX2^+^ mitochondria; some of these showed a distinct dome-shaped morphology, forming a single axodendritic synapse, while others instead were central terminals of synaptic glomeruli, establishing multiple synapses onto postsynaptic structures (Fig. 5). Notably, these central glomerular terminals were occasionally targets of presynaptic axons forming symmetric, presumed inhibitory^48^, synapses onto the central terminal. Dense core vesicles were found in some terminals in the superficial dorsal horn. In laminae III and IV, APEX2^+^ terminals were also observed, although more sparsely. These terminals were often central terminals of glomeruli, but especially in lamina IV, many terminals, despite their generally large size, were found to establish single synapses onto postsynaptic dendrites. As we did not perform full three-dimensional reconstructions of individual APEX2^+^ terminals in serial sections, we could not assess the true number of synapses formed by these terminals.

### A-MNs are required and sufficient for precise nociceptive withdrawal reflexes

**Fig. 6:**
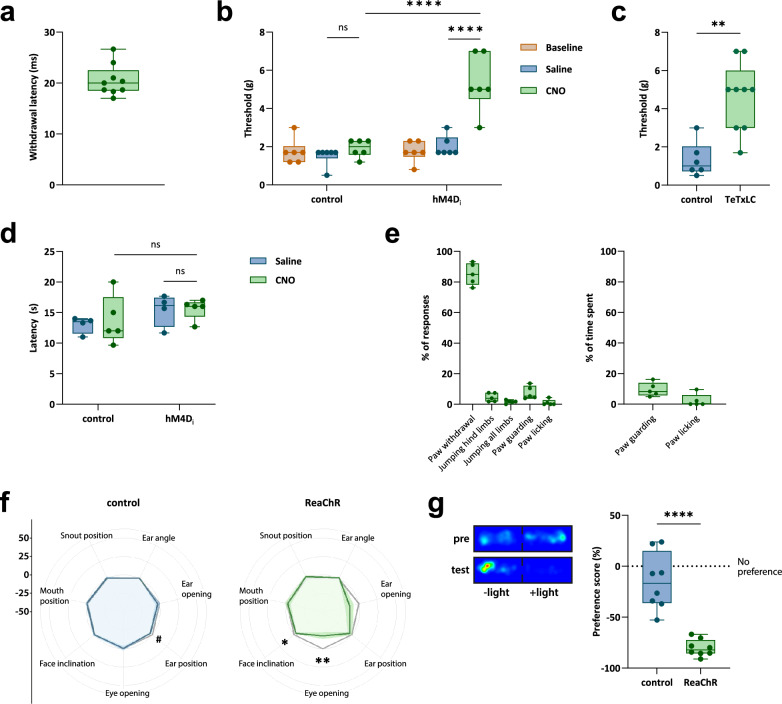
NFH^+^/Na_V_1.8^+^ fibers in nocifensive behavior. **a** Paw withdrawal reflex induced by optogenetic stimulation. For NFH;Na_V_1.8;ReaChR (triple heterozygous) mice, latency to paw withdrawal was 21 ± 3 ms. Control mice did not withdraw their paws (see Supplementary Movie 3). *n* = 9 NFH;Na_V_1.8;ReaChR mice, *n* = 9 control mice. **b**, **c** Mechanical withdrawal reflex thresholds after inhibition of NFH^+^/Na_V_1.8^+^ fibers. **b** NFH;Na_V_1.8;hM4D_i_ (triple heterozygous) and control mice were injected with either CNO or saline. NFH;Na_V_1.8;hM4D_i_ mice showed higher paw withdrawal thresholds after CNO injection. *n* = 6 mice per group. ****, *p* < 0.0001; ns, *p* > 0.05; two-way ANOVA followed by Tukey’s *post hoc* test. Data passed the Shapiro–Wilk normality test. **c** NFH;Na_V_1.8 (double heterozygous) mice were neonatally injected with AAV vectors expressing Cre/Flp-dependent TeTxLC or a control protein. TeTxLC-expressing mice showed higher paw withdrawal thresholds. *n* = 9 TeTxLC mice, *n* = 6 control mice. **, *p* = 0.0016; Mann–Whitney two-tailed test. **d** Hot plate assay. Each mouse was administered either saline or CNO. No significant difference of latencies to mouse licking, flicking or jumping was found between NFH;Na_V_1.8;hM4D_i_ and control mice. *n* = 9 NFH;Na_V_1.8;hM4D_i_ mice (4 saline, 5 CNO), *n* = 9 control mice (4 saline, 5 CNO). ns, *p* > 0.05, two-way ANOVA followed by Tukey’s post hoc test. **e** Nocifensive behavior induced by optogenetic stimulation of the plantar hind paw. Left, percentage of responses of each type relative to all observed responses. Right, percentage of time spent guarding or licking the stimulated paw. *n* = 5 mice. **f** Facial expressio*n* analysis. *n* = 8 NFH;Na_V_1.8;ReaChR mice, *n* = 9 control mice. #, *p* = 0.0358; *, *p* = 0.0318; **, *p* = 0.00770; two-tailed one-sample t-test against baseline. **g** Real-time place preference. NFH;Na_V_1.8;ReaChR mice showed less time spent in the light-on chamber. *n* = 8 mice in each group. The left panel shows an example heat map of time spent in the light-on and light-off chambers for an NFH;Na_V_1.8;ReaChR mouse. ****, *p* < 0.0001; unpaired two-tailed Student’s *t* test. For all box plots, the center line indicates median, box limits indicate interquartile range, and whiskers indicate minimum/maximum values. Detailed statistics are available in Supplementary Data 1. Source data are provided as a Source Data file.

A-nociceptors are generally assumed to have a role in evoking protective nocifensive responses, but this notion has been difficult to test by direct manipulation of such fibers^14,16,26^. Thus, we employed NFH;Na_V_1.8;ReaChR mice to assess behavioral responses to optogenetic stimulation of NFH^+^/Na_V_1.8^+^ fibers. Notably, a single pulse of light (5 ms) applied to the plantar hind paw resulted in a very rapid withdrawal of the paw (Supplementary Movies 1 and 2); using a high-speed camera, we measured the latency to withdrawal from the start of the pulse to 21 ± 3 ms (mean ± S.D.; Fig. 6a). To further characterize the role of these fibers in withdrawal reflexes, we generated NFH;Na_V_1.8;hM4D_i_ mice, where the inhibitory chemogenetic receptor hM4D_i_ is expressed in NFH;Na_V_1.8 fibers. In control mice (positive for NFH^CreERT2^ and hM4D_i_ alleles but negative for Na_V_1.8^FlpO^), i.p. injection of the hM4D_i_ agonist CNO did not affect the mechanical threshold to von Frey filaments (Fig. 6b). However, in mice triple heterozygous for the NFH^CreERT2^, Na_V_1.8^FlpO^, and hM4D_i_ alleles, CNO administration resulted in a marked increase in mechanical threshold. To corroborate this finding, we injected neonatal NFH;Na_V_1.8 mice with an AAV coding for Cre/Flp-dependent tetanus toxin light chain (TeTxLC), which abolishes vesicular glutamate release from the central terminals of neurons expressing the toxin. Compared to NFH;Na_V_1.8 mice injected with a control AAV, these mice, when tested as adults, had a similarly increased mechanical threshold to von Frey filaments applied to the plantar hind paw (Fig. 6c). In contrast to these effects on von Frey thresholds, no effect of chemogenetic inhibition was found with respect to latency to nocifensive responses in the hot plate test (Fig. 6d).

It has been suggested that selective activation of myelinated nociceptors may trigger exaggerated nocifensive responses, including jumping with both hind limbs or all four limbs, and that normal, well-coordinated NWRs restricted to the stimulated hind paw require simultaneous activation of A-LTMRs^16^. However, we found that continuous optogenetic hind paw stimulation of NFH^+^/Na_V_1.8^+^ fibers during a period of 1 min yielded primarily simple, ipsi-/unilateral withdrawal reflexes. Of all nocifensive responses, 85 ± 7% (mean ± S.D., *n* = 5 mice) were such well-coordinated, unilateral reflexes; 5 ± 3% of responses involved jumping with hind limbs, and 2 ± 1% of responses comprised jumping with all limbs (Fig. 6e). Further, 8 ± 4% of responses were guarding of the ipsilateral hind paw whereas 1 ± 2% of responses consisted of paw licking; only two of five mice exhibited paw licking behavior during the time period scored. Guarding and licking episodes covered 9.6 ± 4.5% and 2.4 ± 4.2% of the time, respectively. Thus, we conclude that NFH^+^/Na_V_1.8^+^ fibers are sufficient and necessary for normal, rapid mechanical NWRs, but not heat nocifensive behavior.

### A-MNs can mediate affective pain

To probe a putative role of NFH^+^/Na_V_1.8 fibers in affective aspects of pain, we first used our recently developed tool for facial expression analysis with optogenetic stimulation of plantar hind paw skin^49^. We detected subtle yet significant changes in facial expression reminiscent of a “pain face” (Figs. 6f and S5; see also Supplementary Movie 2). Qualitative inspection of recorded videos showed that salient pain-associated features, such as orbital tightening, were induced by optogenetic stimulation, but occurred only intermittently during the stimulation period. This was likely partly due to the nature of the stimulus; since the manual stimulation of the hind paw resulted in rapid withdrawal and paw guarding/licking, the stimulation was not held at constant intensity or location throughout the stimulation period. To further substantiate a role for these fibers in affective pain, we performed a real-time place preference assay (RTPP) in NFH;Na_V_1.8;ReaChR mice. Unlike littermate control mice, these mice showed a strong aversion to the light-paired chamber (Fig. 6g), confirming that these fibers were able to activate supraspinal pathways mediating affective pain.

### A-fibers are required for NWRs and normal pain sensation in humans

**Fig. 7:**
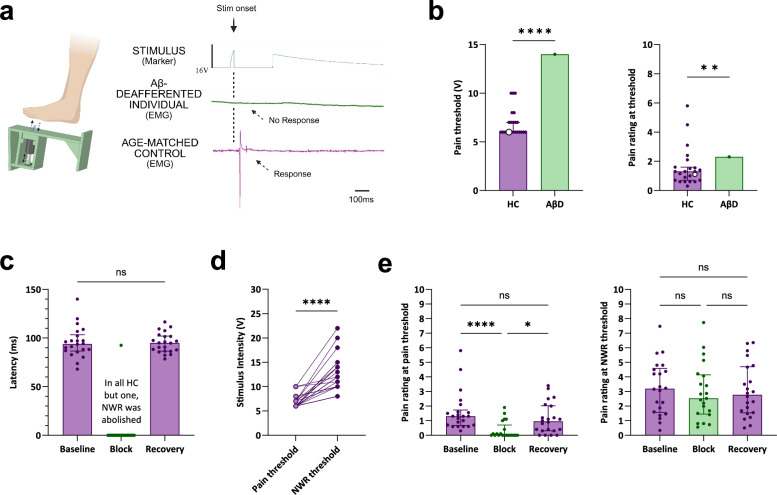
Human nociceptive reflexes and pain psychophysics in healthy control subjects and an Aβ-deafferented individual. **a** Mechanically evoked nociceptive withdrawal reflexes (NWRs) were absent in an individual lacking Aβ fibers. *Left*, experimental setup for rapid mechanical stimulation of the sole of the foot. NWR response was assessed using electromyography (EMG) of m. tibialis anterior. *Right*, example EMG traces from the Aβ-deafferented individual and an age-matched control. The top trace shows the stimulus (Stim) represented as voltage applied to the mechanical NWR apparatus. Note that an NWR response is completely absent in the Aβ-deafferented individual. **b** Pain threshold (*left*) and ratings (*right*) of rapid mechanical stimulation in the Aβ-deafferented individual (AβD) and healthy control subjects (HC; *n* = 23). Large black circles indicate the age-matched control. ****, *p* < 0.0001; two-tailed one-sample t-test against the Aβ-deafferented individual. **c** Effects of Aβ-preferential nerve block on mechanical NWRs. In all HCs except one, NWRs could not be evoked at the NWR threshold. By contrast, NWRs were evoked with similar latencies at baseline and after recovery from the nerve block. ns, *p* = 0.940, two-tailed Mann–Whitney test. *n* = 22 HCs. **d** Mechanical stimulus intensities (represented as applied voltage to the apparatus) to evoke pain were lower than those required to evoke NWR. ****, *p* < 0.0001, two-tailed Wilcoxon matched pairs test. *n* = 22 HCs. **e** Pain ratings at pain threshold (*left*) were lower during nerve block than at baseline (*p* < 0.0001) and after recovery (*p* = 0.014). Pain ratings at the NWR threshold were not significantly affected by nerve block (*right*). Kruskal–Wallis test followed by Dunn’s post hoc test. Error bars in all panels indicate median ±  interquartile range. Detailed statistics are available in Supplementary Data 1. Source data are provided as a Source Data file.

The above observations indicate that A-MNs are essential for mechanically evoked NWRs as well as affective pain. To further test this notion, we turned to a human individual (a 70-year-old male) with a selective Aβ deafferentation and sparing of C-fibers^50^. This individual developed a sensory ganglionopathy from the C3 level downwards and completely lacks Aβ fibers, including Aβ-mechano-nociceptors^51^. We used a custom-made device for rapid punctate mechanical stimulation of the sole of the foot with variable stimulus intensity, and randomized timing to avoid habituation or descending suppression (Fig. 7a). NWR responses were recorded using electromyography (EMG) of the tibialis anterior muscle. Remarkably, mechanical stimulation failed to evoke an NWR at any stimulus intensity up to the individual’s pain tolerance [0–10 visual analog scale (VAS) > 7], unlike in an age-matched control (female, 77 years) (Fig. 7a). Furthermore, mechanical pain threshold (assessed using the same device) was substantially higher in the deafferented subject than in the age-matched control and other healthy control individuals (Fig. 7b); the VAS pain rating at pain threshold was somewhat higher. This is in line with the higher mechanical threshold of C- versus A-nociceptors^51^. Importantly, temperature detection thresholds in the Aβ-deafferented individual were intact, and motor conduction only slightly impaired (Fig. S6). The tested Aβ-deafferented individual is currently the only known living person with selective Aβ deafferentation. Thus, to complement the observations from this individual, we subjected healthy individuals to Aβ-preferential nerve block. Before the nerve block, the individuals exhibited NWR responses to mechanical stimulation at NWR threshold with a median latency of 94 ms (interquartile range 87–104 ms) (Fig. 7c); however, after confirmed establishment of preferential nerve block of Aβ fibers (Fig. S7a–c), the NWR at threshold was almost completely abolished (Fig. 7c). Indeed, NWRs could not be triggered even at the individual’s pain tolerance, although innocuous cool stimuli remained detectable. While detection thresholds were elevated, all participants were still able to perceive cooling within the non-painful range (>10–15 °C)^52^ and heat detection thresholds were preserved, indicating that residual Aδ-fiber input and preserved C-fiber function were not sufficient to trigger NWRs. We further noted that the mechanical threshold (established prior to nerve block) to evoke an NWR was higher than the threshold to evoke pain (Fig. 7d). Pain ratings were strongly attenuated during Aβ preferential nerve block when mechanical stimuli were applied at pain threshold, but at the higher NWR threshold, pain ratings were similar before and after nerve block (Fig. 7e). After recovery from nerve block, NWR responses and pain sensations returned to normal. During the nerve block, cold sensitivity was reduced (Fig. S7c), indicating that cooling-sensitive Aδ fibers were affected in addition to Aβ fibers. In 9/22 individuals, the cold detection threshold (ΔCDT) exceeded −6.5 °C, the upper 95% confidence interval in the sole of the foot for healthy men and women^52^. However, no differences were observed with respect to pain ratings or the ability to elicit NWRs between these individuals and those that retained cold sensitivity within the normal range (Fig. S7d–f). Thus, variation in the degree of cold sensory impairment during the nerve block was not associated with differences in mechanically evoked pain perception or withdrawal reflexes, suggesting that the observed effects are not explained simply by greater impairment of Aδ afferents. Together, these observations indicate that Aβ nociceptors are critical for mechanical NWRs, as well as for normal pain sensation induced by moderate mechanical forces, in humans.

### A-MNs can induce mechanical allodynia and central and peripheral sensitization

**Fig. 8:**
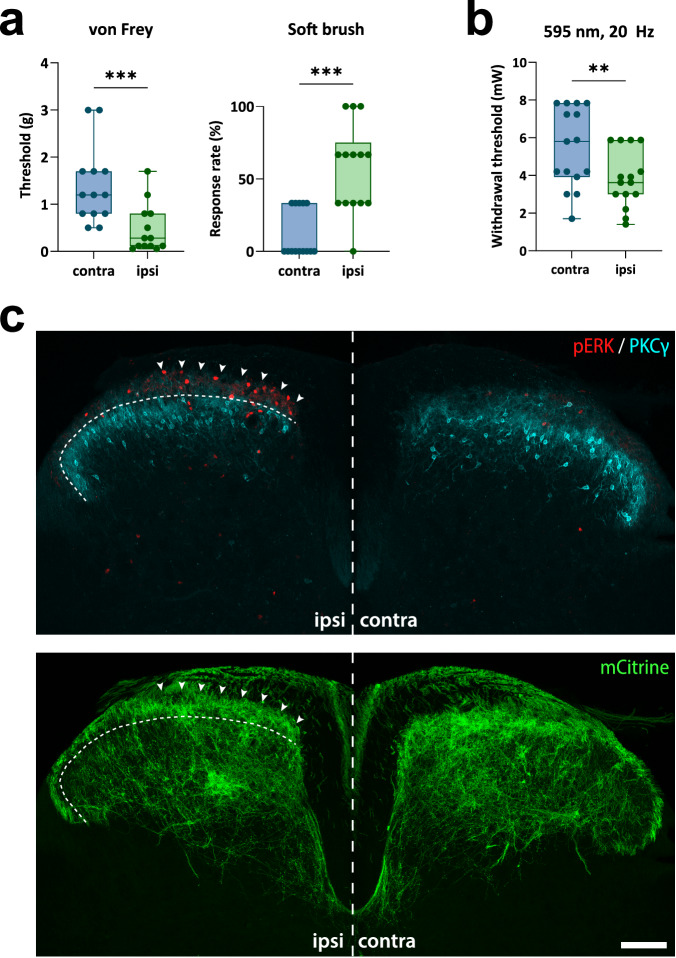
Sensitization by prolonged optogenetic stimulation of NFH^+^/Na_V_1.8^+^ fibers. Optogenetic stimulation (465 nm, 20 Hz, 5 min) was applied to the plantar hind paw of NFH;Na_V_1.8;ReaChR mice. Paw withdrawal reflexes were then assayed with respect to **a** mechanical stimulation (*left*, von Frey assay; *right*, soft cotton brush), and **b** optogenetic stimulation (595 nm, 20 Hz). Hind paws subjected to prolonged optogenetic stimulation (ipsi) were shown to have significantly decreased thresholds compared to the non-stimulated (*contra*) paws. *n* = 13 NFH;Na_V_1.8;ReaChR mice. In **a**, *** indicates *p* = 0.0002 in the left panel and *p* = 0.0005 in the right panel; two-tailed Wilcoxon matched-pairs signed rank test. In **b**, ** indicates *p* = 0.0012, two-tailed paired t-test. For all box plots in (**a**, **b**), the center line indicates median, box limits indicate interquartile range, and whiskers indicate minimum and maximum values. **c** Immunostaining of phospho-ERK (pERK) in the lumbar spinal cord after optogenetic stimulation of the left plantar hind paw. *Top*, pERK and protein kinase Cγ (PKCγ) immunolabeling in a transverse L4 spinal cord section. Note pERK^+^ cells concentrated in a somatotopically appropriate manner in the medial superficial dorsal horn (indicated by arrowheads), receiving input from the hind paw plantar skin. *Bottom*, NFH^+^/Na_V_1.8^+^ fiber innervation in the same section is indicated by mCitrine fluorescence. Thin dashed lines indicate the border between lamina II_i_ and lamina II_o_ as determined from the dorsal border of the PKCγ immunoreactive band. Vertical dashed lines separate the ipsi- (ipsi) and contralateral (*contra*) dorsal horn. Micrographs are maximum intensity projections of 15 optical sections acquired at 1.04 µm separation with a 20×/0.75 objective. Scale bar, 100 µm. The micrographs are representative of independent experiments from five mice. Statistics details are available in Supplementary Data 1. Source data are provided as a Source Data file.

The role of C fiber nociceptors in the induction of pain hypersensitivity is well-established, and these fibers are widely considered to be the primary inducers of central and peripheral sensitization in tissue injury and inflammation^53,54^. By contrast, whether A-nociceptor activation can induce pain hypersensitivity and central sensitization is unknown. As a first step to investigate this, we tested whether strong, prolonged selective stimulation of NFH^+^/Na_V_1.8^+^ fibers could induce sensitization. Indeed, after a 5 min long optogenetic stimulation (20 Hz) of plantar hind paw skin (administered during isoflurane anesthesia) in NFH;Na_V_1.8;ReaChR mice, the mechanical threshold to von Frey filaments after awakening was substantially reduced compared to the contralateral hind paw, while the response rate to soft brushing of the stimulated paw was increased, indicating that prolonged stimulation of NFH^+^/Na_V_1.8^+^ fibers induced both static and dynamic mechanical allodynia (Fig. 8). Further, the light intensity of an optogenetic stimulus needed to evoke paw withdrawal was reduced, suggesting that the sensitization in part was mediated by increased excitability of NFH^+^/Na_V_1.8^+^ fibers. However, prolonged optogenetic stimulation also induced prominent phosphorylation of extracellular signal-regulated kinase (pERK), a well-established marker of central sensitization, in the superficial dorsal horn of the spinal cord (Fig. 8c). Thus, allodynia induced by prolonged stimulation of NFH^+^/Na_V_1.8^+^ fibers could be mediated by both peripheral and central sensitization. These observations suggest the possibility that A-MNs may have a larger and more instructive role in pain hypersensitivity produced by tissue injury than previously envisioned.

## Discussion

We employed an intersectional genetic targeting approach to visualize and manipulate myelinated nociceptors as a broad population in mice. This contrasts with most earlier studies, which have often involved electrophysiological recordings of single fibers, sometimes combined with morphological or neurochemical characterization^1,8,9,30,37,55–57^; these studies have by their nature limited sample sizes and were not designed to assess the function of A-nociceptors with respect to pain behavior. More recently, three subpopulations of A-MN were identified in mice using genetic targeting approaches^11,13,14,16^. One of these populations forms hair follicle endings and has been shown to mediate hair pull pain^13,14^, while another population, targeted using an *Npy2r*^Cre^ mouse line, may be involved in pin prick-mediated withdrawal reflexive behavior^16^. A third type of A-MN, characterized by expression of *Smr2*, has recently been described, but its role in nociception and pain behavior is yet unknown^11^. Notably, all A-nociceptor populations identified so far express CGRP. In the present study, we found that only ~50% of NFH^+^/Na_V_1.8^+^ A-nociceptors have this neuropeptide at detectable levels (in line with previous observations in guinea pig^57^). This strongly suggests that one or more populations of A-nociceptor remain unidentified. A-MNs are assumed to primarily form epidermal free nerve endings. Perl and co-workers used the spatial correlation of post hoc identified cutaneous nerve endings in cat hairy skin with the receptive fields of electrophysiologically recorded A-MNs to support this notion^37^. However, more direct evidence has been sparse. *Npy2r*^Cre^-targeted CGRP^+^ A-nociceptors were indeed found to form free nerve endings in the epidermis^16^, while another CGRP^+^ population instead formed circumferential nerve endings around hair follicles^14^. Here, we confirmed that while certain CGRP^+^ A-MNs form circumferential endings, others that express this peptide form free nerve endings in the epidermis of both hairy and glabrous skin. In addition, we found that A-MNs that lack CGRP also form epidermal free nerve endings in hairy and glabrous skin. No other type of nerve ending was observed in the skin of these mice.

In humans, A-MNs generally form Aβ fibers as evidenced by microneurography^51^ (see Supplementary Note 1); by contrast, single-fiber recordings have indicated a mixed Aδ/Aβ population in mice, with some A-MN fibers having CVs above 10 m/s, above the CV range of D-hair Aδ-LTMRs and overlapping with that of Aβ-LTMRs in this species^3,35^. In accordance with this, we observed that optogenetic stimulation of NFH^+^/Na_V_1.8^+^ fibers triggered both Aδ and Aβ responses. Moreover, soma size, which shows a positive correlation with CV in rodents^17,36^, was similar between targeted neurons in NFH;Na_V_1.8;ReaChR mice and non-targeted NFH^+^, presumed A-LTMR, neurons. Notably, among NFH^+^/Na_V_1.8^+^ fibers in the sciatic nerve, those with undetectable CGRP were considerably larger than those expressing this peptide, suggesting that A-MNs with Aβ fibers in the mouse are preferentially non-peptidergic.

Conventionally, A-MNs have been believed to mainly terminate in lamina I and, to a lesser extent, lamina V^9,38^. By contrast, Boada and Woodbury found extensive arborization of some A-MNs throughout the dorsal horn^8^, including in laminae III and IV, which have long been thought to mainly receive direct afferent input from A-LTMRs^38,39,41^. Our present observations suggest that A-MNs provide the most extensive targeting in lamina I and lamina II_o_, while laminae III–IV are more sparsely innervated, and lamina V is largely devoid of innervation, except for its dorsalmost part. Moreover, the innervation of lamina II appears to be dependent on peripheral origin, as only regions of ventral lamina II receiving input from glabrous skin have substantial A-MN innervation. While our observations may appear at odds with those of Boada and Woodbury, re-examination of the reconstructed fibers shown in their study suggests that those fibers that project deeply in the dorsal horn, in fact, show a rather restricted journey into lamina V, with the most dense arborization in laminae III–IV^8^. Thus, we argue it is warranted to revise the general view of the spinal termination of A-MNs, such that these fibers terminate primarily in laminae I–II_o_, less densely in laminae III to dorsal lamina V and only very sparsely in mid-to-ventral lamina V, while some A-MNs innervating glabrous skin provide prominent innervation of the middle and inner parts of lamina II. While the superficial dorsal horn is broadly considered to be an essential hub for the processing of affective components of pain, the role of the deeper dorsal horn in nociception is less well-understood; however, some neurons in laminae III–V have been suggested to drive withdrawal reflexes^58,59^. The A-MN innervation in laminae III–V observed in this study and previously^8^ is therefore appropriately localized to engage spinal reflex circuits, while A-MN terminals in laminae I/IIo are well-localized to activate supraspinal pathways mediating affective pain. Moreover, our electron microscopic observations that many A-MN terminals form synaptic glomeruli, occasionally receiving presynaptic presumed inhibitory synaptic contacts, suggest that signals from A-MNs may be subject to complex processing even at the first synapse, possibly including gating from other sensory input^60^.

Little is known about the function of A-MNs with respect to affective pain and nocifensive behavior. While direct evidence has been sparse, it has generally been thought that one major role is to mediate NWRs^16,26^. However, activation of the *Npy2r*^+^ subpopulation of A-MN induced exaggerated withdrawal reflexes, which also involved withdrawal of the contralateral hind paw, and often guarding and jumping; only concurrent activation of low-threshold Aβ-fiber mechanoreceptors (A-LTMRs) would yield an appropriate, well-coordinated reflex^16^. Nevertheless, here we found that a broader activation of A-MNs did not induce an exaggerated reflex but instead a much more restricted and precise reflex of the stimulated paw, with only repeated stimulation evoking licking or guarding. Although we cannot exclude that a small population of A-LTMRs may have been targeted in our mice, which could influence these observations, we found neither any innervation indicating association with specialized receptor structures associated with A-LTMRs in the glabrous skin, such as Meissner corpuscles or Merkel cell complexes, nor responses of targeted DRG neurons to low-threshold mechanical stimulation in in vivo Ca^2+^ imaging. Thus, it is clear that activating A-MNs in glabrous skin is sufficient to induce well-defined and precise NWRs.

C-nociceptors, which have been widely implicated in nociceptive reflexes^19,20,22–25^, were not able to rescue mechanical NWRs in a human Aβ-deafferented individual, nor in healthy human individuals subjected to Aβ-preferential nerve block. In line with a critical role for A-MNs but not C-nociceptors in mechanical NWRs, von Frey thresholds for such reflexes were increased ~3-fold in mice where A-MN signaling was impeded. A previous report found a more moderate (roughly 40–50%) increase in mechanical threshold during optogenetic inhibition of A-MNs in rats^26^; the much stronger increase observed here could possibly be attributed to more complete inhibition of A-MNs conferred by hM4D_i_-mediated hypoexcitability or TeTxLC-mediated abolition of synaptic transmission. Optogenetic activation of MrgprD^+^ C fibers evokes paw withdrawal with a latency exceeding 700 ms, while activation of TRPV1-lineage fibers (which include C- and some A-nociceptors) yields withdrawal latencies of ~150 ms^61^. This is substantially slower than the latency of ~50 ms observed for pin prick-evoked NWRs^16,61^. By contrast, the ~20-ms latency for paw withdrawal induced by optogenetic activation of A-MNs observed here (and previously for *Npy2r*^+^ fibers^16^) appears well-suited to mediate mechanical NWRs, allowing for both nociceptor signaling, spinal processing, efferent signaling and muscle activation within the time course for such reflexes. Thus, we conclude that A-MNs are necessary and sufficient for mechanical NWRs, whereas behavioral responses observed after MrgprD^+^ C-mechanonociceptor activation are not attributed to spinal reflexes but instead depend on supraspinal, potentially including cortical, processes.

In addition to a fundamental role in NWRs, we found that activation of NFH^+^/Na_V_1.8^+^ fibers was also sufficient to trigger affective pain and aversive behavior, in line with what has been reported for a hair follicle-innervating A-MN subpopulation^14^. This was mirrored in human experiments, where an Aβ-deafferented individual or healthy individuals subjected to Aβ-preferential nerve block reported deficits in pain perception. However, in a parallel study, we have recently identified an A-MN subpopulation characterized by its expression of TrkC, which mediates NWRs but does not appear to be involved in affective pain behavior^62^. Thus, there appears to be a functional division of different cutaneous A-MN populations. Further efforts are needed to untangle the contributions of different subpopulations of A-nociceptor to different aspects of nociception and pain.

An unexpected finding was that prolonged stimulation of NFH^+^/Na_V_1.8^+^ fibers alone induced a marked punctate and dynamic mechanical allodynia. This phenomenon appears to rely on central sensitization, as evidenced by the concomitant prominent phosphorylation of ERK in the spinal cord^63^, but potentially also on an increase in excitability of NFH^+^/Na_V_1.8^+^ fibers themselves, as suggested by the decrease in the light intensity required to activate these fibers by optogenetic means to evoke a withdrawal reflex.

In conclusion, we established intersectional genetic targeting of mouse NFH^+^/Na_V_1.8^+^ neurons as a powerful tool to study A-MN function and morphology. We showed a fundamental and sufficient role for A-MNs as a broad population in the generation of precise and rapid mechanical nociceptive withdrawal reflexes, but also in affective-motivational aspects of pain in mice and humans. We further provided detailed morphological views of A-MN processes, confirming epidermal free nerve endings as the major form of cutaneous innervation and showing unexpectedly restricted spinal cord termination in lamina V but rich lamina II innervation from glabrous skin A-MNs. Finally, we discovered a form of sensitization and allodynia induced by selective stimulation of A-MNs, suggesting an expansive role for these fibers in not only acute nociception but also in long-lasting and pathological pain.

## Methods

### Animals

The previously reported NFH^CreERT2^^31^ and Na_V_1.8^FlpO^^32^ mouse lines were crossed with each other and with dual Cre/Flp-dependent reporter mice to generate intersectional triple-crosses; alternatively, NFH^CreERT2^;Na_V_1.8^FlpO^ mice were used for adeno-associated virus (AAV) injections (see below). The following reporter mice were used: R26-LSL-FSF-ReaChR-mCitrine (JAX #024846; here called ReaChR mice), ROSA26DR-Matrix-dAPEX2 (JAX #032764; here called APEX2 mice)^64^, Ai195 (JAX #034112; here called GCaMP7s), RC::FPDi (JAX #029040; here called hM4D_i_)^65^. All reporter mice were obtained from Jackson Laboratory. Resulting crosses were on a mixed 129S × C57Bl/6 background. Animals were housed in standard laboratory cages with ad libitum access to food and water, under a 12:12 h dark:light cycle at 22 ± 2 °C and a humidity of 55 ± 10%. All animal experiments were approved by the Animal Ethics Committee at Linköping University (permits no. 2439-2021 and 214-2021) or Uppsala tingsrätt (5.8.18-01428 and 5.8.18-09954) and performed in accordance with the EU Directive 2010/63/EU.

### Tamoxifen injection

For NFH;Na_V_1.8;ReaChR, NFH;Na_V_1.8;APEX2, NFH;Na_V_1.8;GCaMP7s, NFH;Na_V_1.8;hM4D_i_, or AAV-injected NFH;Na_V_1.8 mice, tamoxifen (T5648, Sigma-Aldrich; 15495719, Fisher Scientific) dissolved in corn oil at a concentration of 20 mg/mL was administered at 8–10 weeks of age by either a single injection or by two injections (100 μL, i.p.). The mice were used for further experiments no earlier than 2 weeks after injection.

### Transganglionic neuronal tracing

In 11-week-old triple heterozygous NFH;Na_V_1.8;ReaChR mice of either sex, CTB conjugated to CF594 (CTB^CF594^; Biotium) was under isoflurane anesthesia (4% induction, 1.5% maintenance) intradermally injected into several glabrous skin sites, including digits 2–4, the plantar skin between the pads, and the heel (1 mg/mL, 1 µL per site). Six days after CTB^CF594^ injection, the animals were subjected to perfusion fixation as described below, and the lumbar enlargement of the spinal cord was collected. Transverse vibrating microtome sections (50 µm thickness) were mounted on slides and coverslipped with Prolong Glass or SlowFade Diamond.

### Tissue preparation

Adult (13–16 weeks old) mice of either sex were anesthetized (100 mg/kg sodium pentobarbital or a mixture of 120 mg/kg ketamine and 0.5 mg/kg dexmedetomidine, i.p.) and transcardially perfused using 5 mL phosphate buffer (PB, 0.1 M pH 7.4) followed by 50 mL of fixative (for light microscopy, 4% paraformaldehyde; for electron microscopy, 2% paraformaldehyde and 2.5% glutaraldehyde). Tissues of interest were post-fixed overnight at 4 °C and then stored in 1/10 fixative or PB until further processing.

### Immunofluorescence

Dissected tissue specimens were cryoprotected in 30% sucrose, embedded in OCT, and cut at 15 μm (or, where noted, 100 µm) thickness in a cryostat. Some tissue specimens were instead embedded in 4% low-melting agarose (Fisher Scientific #10377033) and sectioned into 50 µm thick sections on a vibrating microtome (Campden Instruments 7000smz-2). Slide-mounted or free-floating sections were incubated in blocking solution [phosphate-buffered saline (PBS) with 3% normal goat serum, 0. 5% bovine serum albumin and 0.5% Triton X-100] for 1 h, and then in primary antibody cocktail (diluted in blocking solution; see Supplementary Table 1) overnight. After rinsing in PBS or PBS with Triton X-100, the sections were incubated in blocking solution containing appropriate fluorophore-conjugated secondary antibodies (Supplementary Table 2; all at 1:500 dilution). To detect isolectin B_4_ (IB_4_) binding sites, biotinylated IB_4_ (Life Technologies, I21214) was included in the primary antibody solution at 1:1000 dilution; biotin was detected using streptavidin-Alexa Fluor 568 (1:250–500; Life Technologies, S11226) in the secondary antibody solution. In some instances, DAPI or SYTOX Deep Red (ThermoFisher Scientific, S11380) were used to visualize cell nuclei. All incubations were performed at room temperature. After coverslipping with Prolong Diamond, Prolong Glass or SlowFade Diamond (Life Technologies), the sections were examined using Zeiss LSM800 (10×/0.3, 20×/0.8, 40×/1.3 and 63×/1.4 objectives) using Zen 3.1 software or Leica Stellaris 5 (25×/0.95, 40×/1.25 and 63×/1.4 objectives) using LAS X v4.8.0 software.

### In situ hybridization

In situ hybridization of DRG tissue was performed using the RNAScope protocol for frozen fixed tissue sections. Cryostat sections (15 μm thickness) were first dehydrated and pretreated with 0.3% hydrogen peroxide and protease IV before the incubation in *Scn10a* probe (ACD Bio, cat # 426011) solution. After probe incubation, the sections were washed using the probe wash buffer. For amplification, the sections were then incubated in *Amp1*, *Amp2*, *Amp3*, HRP and OPAL 620 (1:1500 in TSA buffer) solutions. The amplification was stopped by incubation in HRP-blocker solution. The sections were coverlsipped with Prolong Diamond and imaged in a Zeiss LSM800 confocal microscope.

### Image analysis

For analysis of DRG neuron soma sizes, z-stacks through the full thickness of DRG sections labeled for mCitrine and NeuN were obtained (24–33 optical sections at 0.53 μm separation) using a Zeiss LSM800 confocal microscope and a 20×/0.8 objective. The resulting z-stacks were manually analyzed in Fiji v1.54p. For each mCitrine^+^ and mCitrine^−^/NeuN^+^ DRG neuron, the maximal cross-sectional area in the z-stack was determined. Neurons where the largest cross-sectional area was observed in the first or last optical section of the z-stack were excluded. To measure the diameters of mCitrine^+^ fibers in the sciatic nerve, each sciatic nerve section (one section per animal), triple immunolabeled for GFP, CGRP and MBP, was imaged in full as a tile scan using a Zeiss LSM800 confocal microscope and a 63×/1.4 oil immersion objective and analyzed in Fiji. For each mCitrine^+^ fiber, the outer border of the myelin sheath, as determined by MBP immunoreactivity, was outlined and the minimum Feret diameter measured. All mCitrine^+^ axons in the nerve section with a well-defined MBP^+^ myelin sheath and a circularity (defined as $4\pi \,\times {area}/{perimete}{r}^{2}$) greater than 0.85 were included in the analysis.

### Electron microscopy

Lumbar spinal cord from triple heterozygous NFH;Na_V_1.8;APEX2 mice (4 females, 13–16 weeks old) was embedded in 4% low-melting agarose and transverse 150 µm sections cut on a vibrating microtome. For APEX2 histochemistry, sections were pre-incubated in 3,3′-diaminobenzidine (DAB; Vector Laboratories) without H_2_O_2_ for 30 min, and subsequently incubated in DAB with H_2_O_2_ for 90 min. The tissue was then osmicated with 1% OsO_4_, counterstained en bloc using 1% uranyl acetate (Electron Microscopy Sciences) in 50% ethanol, dehydrated in a graded series of ethanol and embedded in Durcupan ACM (Sigma-Aldrich #44610) using a standard protocol. Ultrathin sections (70 nm thickness) were cut and placed on single-slot copper grids. Some sections were counterstained using 2% uranyl acetate in H_2_O and 0.5% lead citrate in H_2_O, while others were used with en bloc staining only. Sections were examined in a JEOL JEM1400 Flash transmission electron microscope at 80 kV with EMSIS RADIUS 2.1 software.

### In vivo imaging

In vivo Ca^2+^ imaging of DRGs were performed in NFH;Na_V_1.8;GCaMP7s mice (11–16 weeks old) essentially as previously described^31^. Each mouse was anesthetized with isoflurane (4% induction, 1.5% maintenance) and placed on a custom-made surgical platform. A heating pad was used to maintain body temperature. The dorsal aspect of the spinal cord was surgically exposed between the T12-L1 vertebrae and stabilized with spinal clamps. L4 DRGs were exposed using a dental drill, after which the mouse was transferred to the stage of a custom light microscope (Thorlabs Cerna) with a 4×/0.28 air objective (Thorlabs). GCaMP7s fluorescence images were acquired using ImageJ/µManager 2.0 for 40 s epochs at 5 Hz with an sCMOS camera (Sona 4.2, Andor) using a standard green fluorescent protein (GFP) filter cube. A battery of stimuli was applied to the glabrous skin of the ipsilateral hind paw. The stimuli included a gentle brush (using a puffed-up cotton bud), a set of von Frey filaments (0.16, 0.60, 1.40, 8.00, and 26.0 g), pinblock, and temperature (22 °C → 48 °C → 22 °C). The pinblock stimulus was applied using a resin 3D printed block with an evenly spaced array of 3 ×  10 pins; the pin tips had a diameter of ca 100 µm and were spaced 2 mm apart along the width and length axes of the block. The hind paw was placed on a 3D printed flexible cushion that limited total downward force to around 3 N. In a separate set of experiments, the stimulus battery consisted of pinblock, a cold ramp (34 °C → 10 °C → 34 °C) and a heat ramp (34 °C → 10 °C → 34 °C). Analysis of Ca^2+^ imaging was performed as previously described^14^. Regions of interest (ROI) of responding cells were outlined in Fiji, and the relative change of GCaMP7s fluorescence (Δ*F*/*F*) was calculated. Background signal (e.g., from out-of-focus tissue and neighboring cells) was removed using a custom MATLAB script^14^ by subtracting the fluorescence of a donut-shaped area surrounding each ROI. The response threshold was set to 10%.

### Ex vivo optogenetics and electrophysiology

#### Conduction velocity measurements

NFH;Na_V_1.8;ReaChR mice (ages 2–3 months) were anesthetized (0.5 mL isoflurane for 1–2 min) and euthanized by cervical dislocation. The vertebral column was extracted, and lumbar dorsal root ganglia (DRG) with connected dorsal roots were gently harvested in a continuously oxygenated (95% oxygen, 5% carbon dioxide) ice-cold solution (composed of, in mM: 93 *N*-methyl-d-glucamine, 2.50 KCl, 1.20 NaH_2_PO_4_, 30 NaHCO_3_, 20 HEPES, 25 glucose, 5 sodium ascorbate, 2 thiourea, 3 sodium pyruvate, 10 MgSO_4_, 0.5 CaCl_2_). The root-connected DRGs were subsequently incubated for 30 minutes at 36 °C in continuously oxygenated (95% oxygen, 5% carbon dioxide) aCSF (in mM: 126 NaCl, 2.5 KCl, 1.25 NaH_2_PO4, 26 NaHCO_3_, 10 glucose, 1.5 CaCl_2_, 1.5 MgCl_2_). The root-connected DRG was then transferred to a recording chamber, where it was continuously perfused with oxygenated (95% oxygen, 5% carbon dioxide) aCSF held at a temperature of 34–36 °C using a HPT-2 heated perfusion tube (ALA Scientific Instruments Inc.) controlled by a Scientifica temperature controller (Scientifica, UK). For electrical stimulation the DRG was stimulated with a suction pipette connected to an A365 Stimulus Isolator (World Precision Instruments); for optogenetic stimulation of ReaChR expressing DRG neurons, a blue light (415 nm) from a fluorescent LED light source (CoolLED system, Andover, United Kingdom) was applied through a 10x water immersion objective (LUMPlan FI, 0.90 numerical aperture (NA), Olympus). Evoked compound action potentials (CAP) were measured at the distal end of the dorsal root via a suction pipette (0.5 MΩ) filled with aCSF. Signals were amplified with a MultiClamp 700B amplifier (Molecular Devices, San Jose, CA), digitalized at 20 kHz with Digidata 1440A (Molecular Devices), low-pass filtered at 10 kHz, acquired in WinWCP software (Dr. J. Dempster, University of Strathclyde, Glasgow, United Kingdom), and analyzed in Clampfit 11.2 (Molecular Devices). The conduction velocity (CV) was calculated by dividing the distance from the stimulated DRG to the recording pipette by the time latency from the stimulation to the signal detected by the recording pipette [Eq. 1]. In contrast to the instant activation upon electrical stimulation, the ReaChR activation kinetics introduce error to the latency. Thus, the ReaChR activation delay was also measured and subtracted from the total latency to calculate the correct CV [Eq. 2].

1 $${Conduction}\,{velocity}\,({electrical})=\frac{{\mathrm{Distance}}}{{\mathrm{latency}}}$$

2 $${Conduction}\,{velocity}=\frac{{Distance}}{({Total}\,{latency}-{ReaC}hR\,{activation}\,{delay})}$$

#### ReaChR activation kinetics

To determine the latency of activation of the ReaChR channelrhodopsin, DRGs were collected as above, and then incubated in continuously oxygenated (95% oxygen, 5% carbon dioxide) DMEM (Gibco) cell media containing 0.25% collagenase type IV (Thermofisher) for 1 h at 36 °C (adapted from ref. ^66^). Subsequently, the DRGs were carefully dissociated using a fire-polished glass pipette and the cell suspension centrifuged at 1200 × *g* for 5 min. After removal of the supernatant, the cells were resuspended in the aCSF solution and transferred onto a glass cover slip in a petri dish perfused with continuously oxygenated aCSF solution. After at least 2 h to allow the cells to attach to the cover slip, the petri dish was transferred to the microscope (same as above). ReaChR positive cells (identified by native mCitrine fluorescence) were subjected to cell-attached current-clamp recordings via a recording pipette (7–9 MΩ) filled with the same aCSF solution as above. Electrophysiological recordings and optogenetic stimulation were performed as described above.

### Behavioral assays

#### Optogenetically evoked paw reflex latencies

Mice (either sex, 13–16 weeks old) were placed individually in an observation cubicle (*W* × *D* × *H* 9 × 5 × 5 cm^3^) made of transparent acrylic glass with a 5 mm thick floor, and stimulated on the plantar hind paw. The mice were video recorded from a lateral view using a high-speed camera (a customized C-mount Sony RX0 II (Back-bone, Kanata, Canada)) at 1000 frames/s (yielding a temporal resolution of 2 ms). Optogenetic stimulation was applied to the hind paw (460 nm, single 5 ms pulse) via the tip of a 960 µm/NA 0.63 optical fiber patch cord manually placed immediately below the floor beneath the hind paw.

#### von Frey assays

Mice were placed in a cubicle (*W* × *D* × *H* 9 × 5 × 5 cm^3^) with clear acrylic walls and a mesh floor. After 15 min habituation, the mechanical paw withdrawal threshold was assessed using manual von Frey filaments applied to the plantar hindpaw. The mechanical von Frey threshold was determined using the simplified up-down method^67^.

To determine von Frey thresholds during chemogenetic inhibition, triple heterozygous NFH;Na_V_1.8;hM4D_i_ mice and control littermates (bearing the same alleles except being double negative for Na_V_1.8^FlpO^), 28–29 weeks old, were on day 1 placed in the mesh floor cubicle and the baseline threshold was determined. On day 2, the animals were injected with either saline or CNO (1 mg/kg i.p.), and von Frey threshold was assessed 30 min after injection. On day 3, the animals were again tested after being injected with the solution not administered on day 2; the order of solutions was individually randomized for each mouse. Treatment order and genotype were unknown to the experimenter at the time of testing.

Mechanical paw withdrawal thresholds were also tested in mice, where synaptic transmission from NFH^+^/Na_V_1.8^+^ fibers had been abrogated using TeTxLC. Here, 9 NFH^CreERT2^;/Na_V_1.8^FlpO^ pups (P0–P4) of either sex were briefly anesthetized with isoflurane and injected i.p. with AAV9.Con/Fon-TeTxLC (1 µL of 8.2 × 10^12^ vg/mL solution diluted 1:10 in saline, for a final injected volume of 10 µL). Each pup received two injections, either at P0 and P3 or P2 and P4. The TeTxLC plasmid was constructed by Dr Hendrik Wildner, University of Zürich, and the AAVs were produced by the University of Zürich Viral Vector Facility. As controls, 6 littermate mice with the same genotype were instead injected with an unrelated AAV (AAV9.Con/Fon-syp-mCherry, from the same source; 1 µL of 1.1 × 10^13^ vg/mL diluted 1:10 in saline) at P0 and P3. The mice received tamoxifen injections at 6 weeks age, and were subjected to von Frey assays at 9 weeks as above.

#### Hot plate assay

On two separate days, NFH;Na_V_1.8;hM4D_i_ mice and control littermates (32–35 weeks old) were injected with saline or CNO (1 mg/kg i.p.). After 30 min, the mouse was placed on a hot plate (IITC Life Science analgesia meter) set at a constant temperature of 50 °C, and the latency to jumping or paw licking/flicking was measured. The mouse was immediately removed from the plate after a nocifensive reaction was observed. Each mouse was subjected to three trials (minimum inter-trial interval 15 min) and the average latency calculated. On the second day, the mouse was administered the solution that it did not receive the first day. The order of saline and CNO injections was individually randomized. The experimenter was blind to both treatment order and genotype.

#### Real-time place preference

On day 1 (pre-test), triple heterozygous NFH;Na_V_1.8;ReaChR mice and littermates (same genotype except being FlpO negative), 20–25 weeks old, were placed in a two-chamber clear acrylic glass apparatus (each chamber 175 × 90 mm^2^) connected via a 50 mm wide opening. The floor consisted of 5 mm thick clear acrylic. Mice were video recorded from above using a webcam (Logitech C920) and AnyMAZE 6.33 software (Stoelting). During the pre-test, the mice were allowed to freely explore both chambers for 15 min. The chamber in which each mouse spent most of its time during pre-test was defined as its initially preferred one. On day 2 (test phase), the mice were again placed in the apparatus for a duration of 15 min. During this phase, one hind paw was continuously tracked using an optic fiber tip (NA 0.63, 960 µm core, attached to a Prizmatix 460 nm LED), manually placed under the hind paw directly beneath the floor. When the mouse entered the initially preferred (as determined during the pre-test) chamber, optogenetic stimulation (20 Hz, 5 ms pulses) was switched on automatically via the AnyMAZE software; the stimulation continued until the mouse exited to the other chamber. The preference score $P$ was calculated as $P=100\times ({t}_{{test}}-{t}_{{pre}})/{t}_{{pre}}$, where ${t}_{{test}}$ is the time spent in the light-on chamber during the test phase and ${t}_{{pre}}$ the time spent in the same chamber during the pre-test phase. The experimenter was nominally blind to the genotype of the mice during the experiment. However, because triple heterozygous mice, but not FlpO-negative mice, exhibited clear nocifensive behavior in response to optogenetic stimulation, full blinding was not possible.

#### Facial expression and nocifensive behavior assays

Triple heterozygous NFH;Na_V_1.8;ReaChR mice and littermates (13–17 weeks old) were placed in a transparent acrylic glass cubicle (9 × 5 × 5 cm^3^). The lateral view of the cubicle was recorded using a color FLIR camera (Blackfly BFS-U3-23S3C-C) with a 1:1.8/4 mm Basler lens (C125-0418-5M) at 60 frames/second with OpenBroadcaster Software 27.0.1 (https://obsproject.com). The mouse was habituated for 10 min, after which a 3 min baseline was recorded. Immediately thereafter, optogenetic stimulation was applied to the plantar hind paw through the transparent floor via a fiberoptic ferrule (0.63 NA, 960 µm; 5 ms pulses at 20 Hz using a Prizmatix 460 nm LED or a Doric Lenses 595 nm LED). After stimulation for 3 min, a 3 min recovery period was also recorded. Facial expression analysis was performed as described^49^. For analysis of nocifensive behavior, the first 60 s of the optogenetic stimulation period was analyzed using Noldus The Observer XT 15 software with respect to paw withdrawal, jumping (on hind limbs or all four limbs, counted separately), paw licking and paw guarding. Event counts were obtained for each behavior, as were the durations of licking and guarding behaviors.

#### Prolonged optogenetic stimulation

NFH;Na_V_1.8;ReaChR mice (13–14 weeks, of either sex) were during isoflurane anesthesia (4% induction, 2.5% maintenance) subjected to optogenetic stimulation (465 nm, 20 Hz, 5 ms pulses) for 5 min to the left plantar hind paw. The mouse was immediately thereafter placed in a cubicle with a mesh floor. After 10 min of recovery from anesthesia, a soft brushing stimulus was applied using a puffed-up cotton bud to the hind paw; this was repeated three times, and the response frequency was recorded. Next, the mechanical withdrawal threshold was tested using von Frey filaments (as above) in ipsi- and contralateral hind paws. Thereafter, the light intensity needed to evoke a paw withdrawal by optogenetic stimulation was tested. To assess this threshold, 5 ms pulses (20 Hz, NA 0.63, 960 µm core, attached to a 595 nm LED from Doric Lenses) were applied to the hindpaw from below the mesh floor at five set intensity levels, from the lowest intensity to the first that initiated a withdrawal response. Because the measured power from the LED changed somewhat between experimental sessions, absolute threshold values varied slightly between mice.

A separate group of mice were used to assess spinal phosphorylation of extracellular signal-related kinase 1/2 at Thr^202^/Tyr^204^ (pERK), a marker of central sensitization in the spinal dorsal horn^63^. Here, immediately after the prolonged optogenetic stimulation and under continued isoflurane anesthesia, the mice were subjected to perfusion fixation using 4% paraformaldehyde, after which spinal cord tissue was processed for immunofluorescence as above.

### Human reflex electromyography and psychophysics

#### Participants

Twenty-eight healthy controls (HCs), 18–40 years old (females, 18; males, 10), were originally recruited from an existing database at the Center for Social and Affective Neuroscience (Linköping University) and advertisements on social media sites. Exclusion criteria were diabetes, muscular, skeletal, skin, or neurological diseases, analgesic and/or psychoactive medication. Due to insufficient data (e.g, missing NWR thresholds or incomplete data collection), a total of 22 controls were included in the analysis. The individual with Aβ deafferentation (male, 70 years) is well-characterized and suffers from a rare sensory ganglionopathy syndrome^50^. Because this individual belongs to an older age group, an age-matched individual (female, 77 years) was used for comparison in NWR and thermal threshold testing. Written informed consent was obtained from all individuals before the start of the experiment, and all data processing followed the GDPR guidelines. The study was approved by the Swedish Ethical Review Authority (dnr 2020-04207).

#### Mechanical reflex elicitation

To evoke the reflex mechanically, a custom-built device delivered a single pin prick. A solenoid motor (Mecalectro 819AB83) launched a pin (1 mm diameter) to a maximum travel distance of 9 mm before retracting after each stimulus. An optical sensor placed next to the pin registered the time the pin passed through its hole to signal stimulus onset. The stimulus had a bandwidth up to ~20 Hz, amplitudes up to ~20 µV, and a pulse width of ~400 µs. The mechanical indentation was adjusted by changing the voltage of the power supply (EA-PS 2342-10 B), which ranged from 5 to 35 V. The power sequence was controlled using EasyPS2000 (version 2.05, with LabView runtime, 236 MB, Elektro-Automatik, Viersen, Germany). The timing of the stimulus was manually randomized between 3 s and 1 min to avoid habituation of the reflex and maintain the novelty of the stimulus. A silent computer mouse was used to trigger the device, and a physical partition blocked visual cues.

#### Experimental setup

Participants were seated in a comfortable chair with the right foot hanging freely at a 90–130° angle. The foot was placed on the device platform, at a slight upward angle over a small rectangular hole, allowing the pin to deliver the stimulus just below the anterior lateral eminence of the foot sole, an area corresponding to the reflex receptive field of the tibialis anterior muscle^68^. Two adhesive electrodes were placed 2 cm apart on the gently abraded and degreased skin over the belly of the tibialis anterior muscle on the leg, as anode and cathode, and a third electrode was placed over the patella as reference (Kendall ECG electrodes 57 × 34 mm, Medtronics, USA).

The foot soles were examined after each stimulus to mark the spot where a reflex could be evoked and to ensure that the skin was not sensitized, the absence of redness and any lingering pain or discomfort was confirmed following the stimulus. Reflexes were recorded using PowerLab (16/35 AD Instruments, Oxford, UK), and stored in LabChart (v8.1.16 AD Instruments, Oxford, UK) with a low pass filter of 1 khz and a high pass filter of 0.3 Hz, a range of 1 mV, sampled at a rate of 20,000 Hz with an acquisition delay of 160 µs. A clear deviation from baseline, evoking a reflex response with a z-score >1 was included in the analysis. Reflex latencies were further calculated, post-acquisition, in MATLAB (R2021b, MathWorks Inc, Natick, Massachusetts). A time analysis window of 40–200 ms was used to include both short- and long-latency NWR responses.

#### Pain and reflex thresholds

To assess pain intensity, a visual analog scale (VAS) was used, ranging from 0 representing “no pain” (to the far left) to 10 representing “worst imaginable pain” (to the far right). The mechanical indentation was increased stepwise, in 1 V increments, until the participant reported a painful sensation. This was taken as the pain threshold, i.e., defined as the voltage at which the participant first rated something above 0 on the scale. Pain ratings were further analyzed, post-acquisition, in MATLAB (R2021b, MathWorks Inc, Natick, Massachusetts). Reflex thresholds were defined as the stimulus voltage at which the NWR was elicited three times at the same location on the foot sole. All stimulations were delivered during a relaxed EMG state, without any visible muscle contractions. Once thresholds were established during baseline, stimulus intensities at pain and NWR thresholds were applied a minimum of 3 times during the block and at least once during recovery.

#### Nerve block in healthy individuals

A pressure cuff (Riester Gmbh, Jungigen, Germany) was used to induce an ischemic nerve block on the right foot. Such a nerve block first affects the Aβ afferents, as they are more susceptible to a conduction block than the smaller fibers^69–71^. The pressure cuff used to induce a preferential block of Aβ afferents was placed around the right ankle and inflated to a pressure of 250–300 mmHg for up to an hour^72^. Vibration sensation is known to be mediated by Aβ afferents^73^, and was therefore used to assess large-fiber function before, during, and upon nerve block. Successful establishment of the nerve block was determined primarily by loss of tuning-fork vibration detection, while two additional vibration tasks were performed to further characterize the degree of large-fiber impairment. Specifically, three different vibration tasks were conducted: (1) A simple detection task (a 2 alternative force choice), where a tuning fork (128 Hz, American Diagnostic Corporation, NY, USA) was held against the testing site (foot sole) and individuals were asked to report if they could feel vibration or not in a series of 5 trials. (2) A 3 alternative force choice (3-AFC), where a tactile vibrator (Piezo, Dancer Design, UK) generated vibration frequencies of 200 Hz, 20 Hz, or 0 Hz (no vibration). Individuals were asked to report “high,” “low,” or “no” vibration in a series of 9 trials. (3) In an intensity rating task, where subjects could rate the intensity of vibration, alternating between 20 and 200 Hz, performed 6 times (three of each frequency). The intensity rating task was performed before and during the nerve block. All individuals wore earplugs during all vibration tests to avoid auditory cues.

#### Temperature detection thresholds

As Aδ and C fibers mediate cold and warm sensations, respectively^74,75^ quantitative thermal testing (TCSII, Strasbourg, France) was performed to determine cold and warm detection thresholds (CDT and WDT) at a rate of 1 °C/s in a series of 4 trials before, during, and after nerve block. During the initiation of the nerve block, brass rods immersed in ice or hot water (45 °C) were placed under the metatarsophalangeal joint of the foot sole every ~5 min to detect differences in thermal sensibility between feet before conducting quantitative sensory testing. In the Aβ deafferented individual and age-matched individual (age-matched control), thresholds were assessed three times before reflex testing at 1 °C/s, in a series of 4 trials. All baseline temperatures were set to the individual’s skin temperature, and CDT and WDT thresholds are reported as baseline temperature – threshold temperature (or delta thresholds ΔCDT and ΔWDT).

#### Nerve conduction velocity testing in the Aβ-deafferented individual

To ensure that any effect on the NWR was not due to slow conducting efferent fibers, nerve conduction velocity testing was conducted on the individual with Aβ deafferentation using standard laboratory equipment (Nicolet EDX, Natus Neurology Incorporated, Middletown, Wisconsin, USA). Recording electrodes (Nicolet Biomedical, EMG surface electrode, Cephalon, Denmark) were attached to the belly of the tibialis anterior, extensor digitorum brevis, and the abductor digiti minimi muscles 2 cm apart and connected to a stimulator (Nicolet AT2+6 amplifier, Natus Neurology Incorporated, Middletown, Wisconsin, USA). Stimulating electrodes (Nicolet Biomedical, EMG surface electrode, Cephalon, Denmark) were placed over the ulnar, peroneal, and tibial nerves. Conduction velocities (CV) were automatically calculated between a distal and proximal site on all nerves by the computer software (Synergy CareFusion EDX, V.20.0, 2010, Middleton, Wisconsin, USA).

### Statistics

All descriptive statistics and statistical tests were performed in GraphPad Prism v10.5. Normal distribution of data was assessed using the Shapiro–Wilk normality test. All statistical tests were two-tailed. Measures are given as mean ± SD or as median ±  interquartiles and range, as appropriate.

### Reporting summary

Further information on research design is available in the Nature Portfolio Reporting Summary linked to this article.

## Supplementary information

Supplementary Information

Description of Additional Supplementary Files

Supplementary Data 1

Supplementary Movie 1

Supplementary Movie 2

Supplementary Movie 3

Reporting Summary

Transparent Peer Review File

## Source data

Source Data

## Data Availability

All quantitative data underlying statistical analyses are available in the Source Data file. Because of the large size of the raw image and video files, the data were not deposited in a public repository. All data are available from the corresponding authors upon request. Source data are provided with this paper.
