# Supplementary material for: Central role for fast nociceptors in mechanical nocifensive behavior and sensitization
Source: Nat Commun. 2026 Jul 25;17:7271. doi: 10.1038/s41467-026-75948-z (PMC13401607; doi:10.1038/s41467-026-75948-z)
Supplement: Supplementary file 1 — Supplementary Information [file 41467_2026_75948_MOESM1_ESM.pdf]

Supplementary information

Central role for fast nociceptors in mechanical nocifensive behavior and sensitization

John Chwen-Yu Chen[1], Oumie Thorell[2,3], Felipe Meira de-Faria[2], Aikeremu Ahemaiti[4], Olivia Le Moëne[1], Lech Kaczmarczyk[1,5], Katharina Henriksson[3], Jonathan Cole[6], David A Mahns[3], Håkan Olausson[2], Walker S Jackson[1,5], Saad S Nagi[2,4], Malin C Lagerström[4], Marcin Szczot[2], Max Larsson[1]

[1]Department of Biomedical and Clinical Sciences, Division of Cell- and Neurobiology, Linköping University, Linköping, Sweden

[2]Department of Biomedical and Clinical Sciences, Centre for Social and Affective Neuroscience, Linköping University, Sweden

[3]School of Medicine, Western Sydney University, Sydney, Australia

[4]Department of Immunology, Genetics and Pathology, Uppsala University, Uppsala, Sweden

[5]Department of Biomedical and Clinical Sciences, Wallenberg Centre for Molecular Medicine, Linköping University, Linköping, Sweden

[6] Clinical Neurophysiology, University Hospitals Dorset and Bournemouth University, Poole, UK.

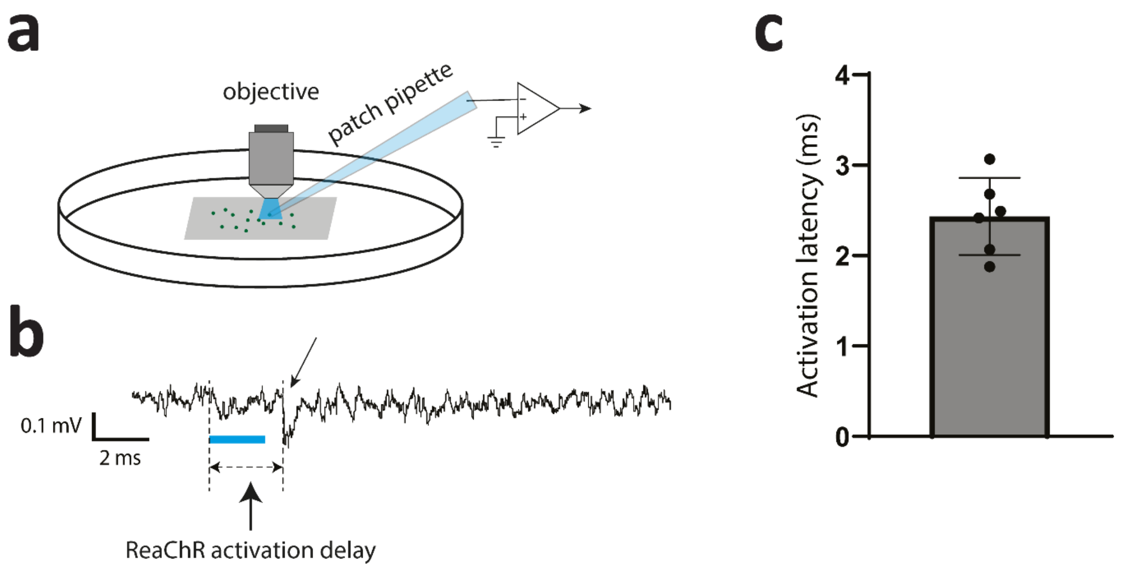

Figure S1. Measurement of ReaChR activation kinetics in DRG neurons. (**a**) Schematic of the setup. Primary mCitrine$^+$ DRG neurons isolated from NFH;$Na_V1.8$;ReaChR mice were subjected to optogenetic stimulation while patch-clamped. (**b**) An example voltage trace shows a response (arrow) to a 2 ms light pulse (blue line). The ReaChR activation delay was measured from the start of the light pulse to the initiation of the activation response (marked with dash lines and arrows). **(c)** A summary plot of all measured ReaChR activation delays. Error bars indicate mean ± S.D. *n* = 6 cells. Source data are provided as a Source Data file.

a

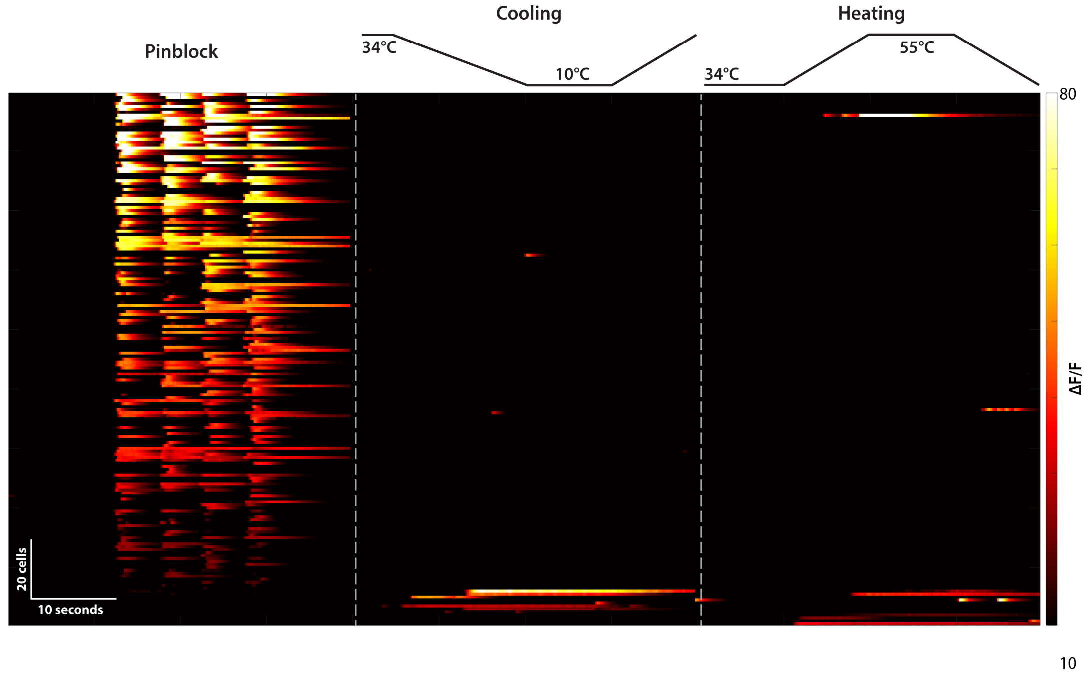

b

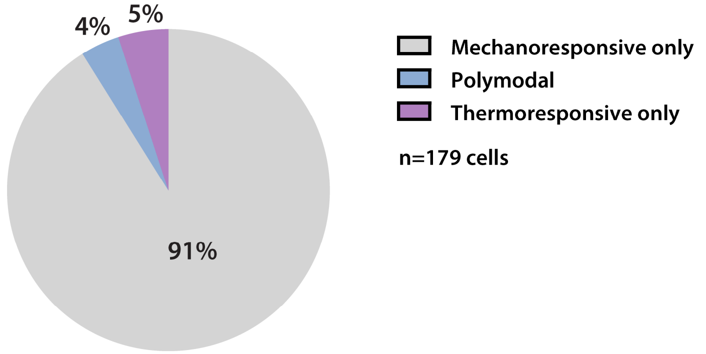

Figure S2. Thermal responses of NFH^{+}/NaV1.8^{+} fibers. (a) Assessment of cold and heat responses of NFH^{+}/NaV1.8^{+} DRG neurons using *in vivo* Ca^{2+} imaging. Shown is a heat map of responses of GCaMP7s^{+} neurons in L4 DRGs to a pinblock stimulus (used to identify A-MNs), a cold ramp stimulus and a heat ramp stimulus, applied as indicated. Each row indicates the responses of an individual cell. *n* = 179 cells, collected from three L4 DRGs (one ganglion per mouse). **(b)** Percentages of cells responsive to pinblock alone (Mechanoresponsive only), to both pinblock and either thermal stimulus (Polymodal) or only to one or both thermal stimuli (Thermoresponsive only). Source data are provided as a Source Data file.

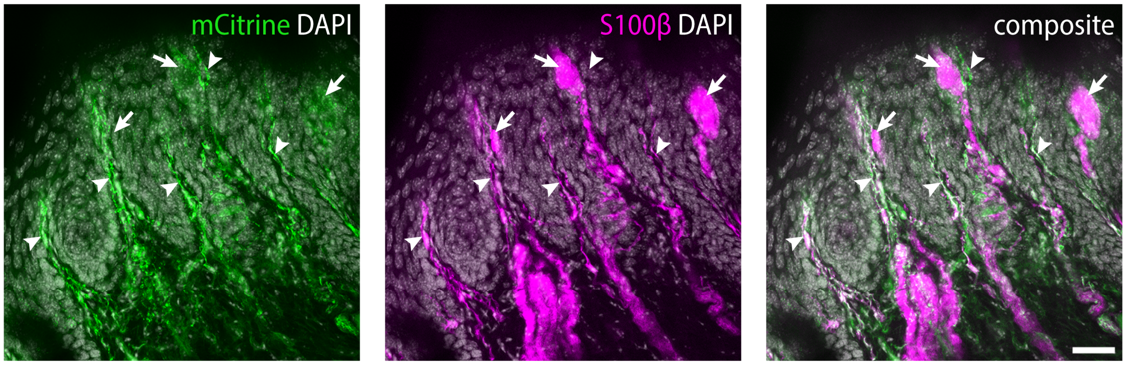

Figure S3. NFH⁺/Na$_V$1.8⁺ fibers do not innervate Meissner corpuscles. Thick (100 µm) plantar hind paw sections were co-immunolabeled for mCitrine and S100β, a marker of myelinated afferent fibers, Schwann cells and Meissner corpuscles. mCitrine⁺ fibers often entered the epidermis via dermal papillae (arrows), but were excluded from Meissner corpuscles (arrows). Scale bar, 25 µm. Images are maximum intensity projections of 19 optical slices obtained at 0.36 µm separation using a 40x/1.25 glycerol immersion objective. The micrograph is representative of observations from three mice.

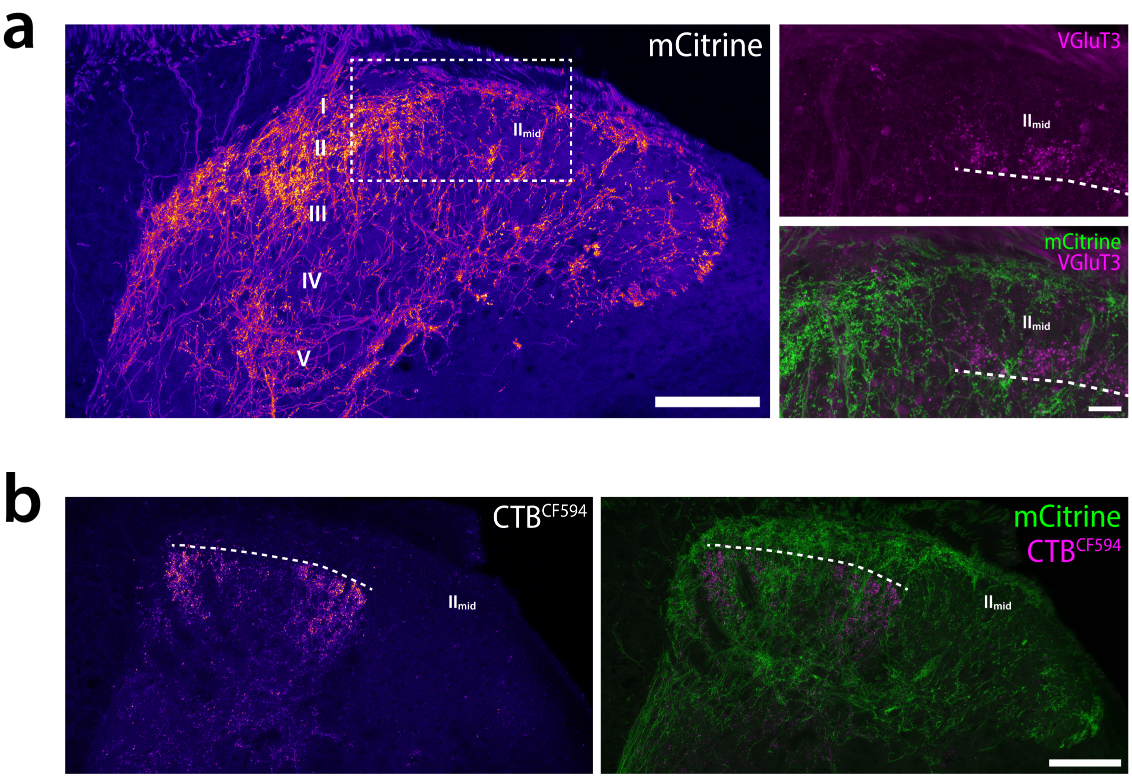

Figure S4. Dense termination in lamina II of NFH⁺/Na$_V$1.8⁺ afferent fibers innervating glabrous skin. (a) *Left,* transverse section of C7 spinal cord in NFH;Na$_V$1.8;ReaChR mice showing dense innervation by mCitrine⁺ fibers in mid-lamina II (**II$_{mid}$**) of the medial but not lateral dorsal horn. Dashed frame indicates the region magnified in the right panels showing VGluT3 immunoreactivity, a marker of C-LTMR terminals, and mCitrine fluorescence. Dashed lines in the right panels show the mediolateral extent of VGluT3⁺ C-LTMR terminals, which are restricted to the parts of lamina II$_i$ receiving input from hairy skin. Note that mCitrine innervation is sparse in lamina II$_{mid}$ dorsal to the band of VGluT3⁺ terminals, while VGluT3⁺ terminals are not found in medial lamina II showing dense innervation of mCitrine⁺ fibers. Roman numerals indicate Rexed's laminae. **DF**, dorsal funiculus. Scale bar in left panel, 200 µm; scale bar in bottom right panel is 20 µm, valid also for top right panel. Maximum intensity projection of 15 optical slices acquired at 0.36 µm separation with a 40x/1.25 glycerol immersion objective. **(b)** Transverse section of L4 spinal cord from NFH;NaV1.8;ReaChR mice subjected to transganglionic tracing of cholera toxin B subunit conjugated to CF594 (CTB^{CF594}) from hind paw glabrous skin. Right panel shows CTB^{CF594} fluorescence pseudo-colored using the Fire look-up table in Fiji to enhance the visibility of labeled terminals. Dashed line indicates the mediolateral extent of CTB^{CF594}-labeled nerve endings in the medial part of the dorsal horn. Note that CTB^{CF594}-labeled terminals originating from glabrous skin afferents are excluded from the lateral region of the dorsal horn with sparse mCitrine⁺ innervation in lamina II$_{mid}$. Scale bar, 50 µm, valid for both panels. Maximum intensity projection of 15 optical slices obtained at 0.36 µm separation using a 40x/1.25 glycerol immersion objective. Micrographs are representative of experiments from three mice.

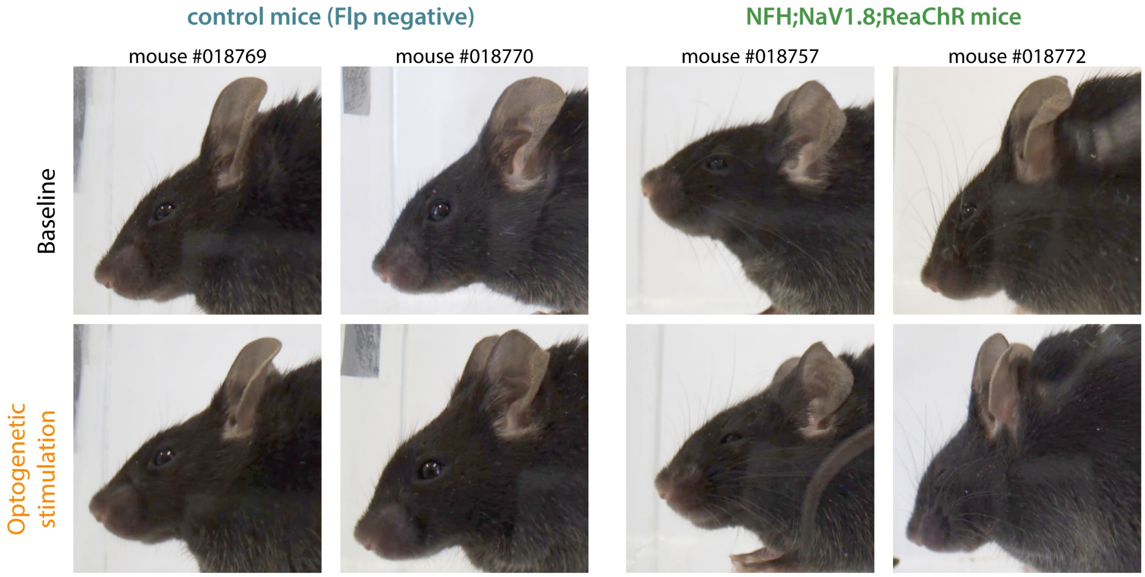

Figure S5. Facial expression changes induced by optogenetic stimulation of NFH^{+}/Na$_{V}$1.8^{+} fibers. Shown are example frames extracted from video recordings of triple heterozygous NFH;Na$_{V}$1.8;ReaChR mice and Flp-negative litter mate control mice during baseline and during optogenetic stimulation (595 nm, 5 ms pulses, 20 Hz) of the plantar hind paw. Each column shows an individual mouse during baseline and stimulation phases. Note that the baseline image of mouse #018770 and the optogenetic stimulation image of mouse #018772 have been horizontally flipped and thus show the right side of the face.

a

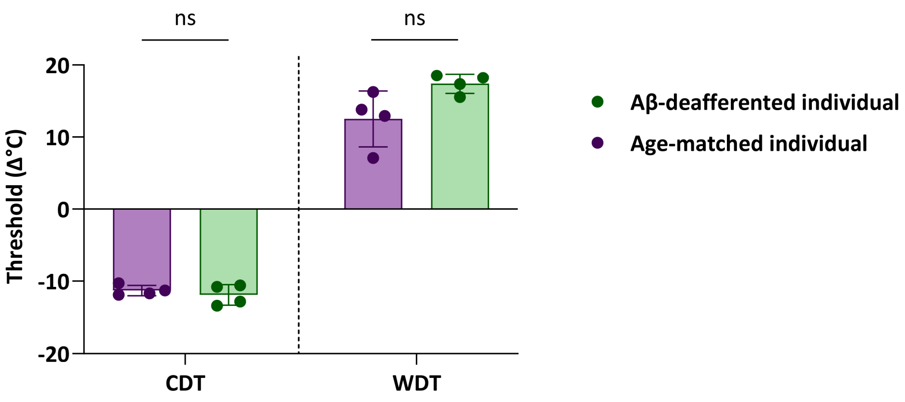

b

	Latency (ms)	Amplitude (mV)	Amplitude (%)	Conduction velocity (m/s)
**R Ulnar – ADM fractioned**				
Wrist	3.28	9.4	100	
Under Elbow	7.86	4.1	43.8	44.7
Above Elbow	10.42	7.8	82.9	37.2
Axil	13.91	5.8	61.5	45.9
**R Peroneal**				
Ankle	6.56	2.8	100	
Knee	18.13	2.3	82.9	34.6
**R Tibial**				
Ankle	6.46	1.3	100	
Knee	18.65	0.8	59	36.1

Figure S6. Assessment of peripheral nerve fiber function in the Aβ-deafferented individual. (**a**) Temperature detection thresholds. Cold (**CDT**) or warm (**WDT**) temperature detection thresholds did not differ between the Aβ-deafferented individual and the age-matched individual, suggesting that the absence of an NWR in the Aβ-deafferented individual was likely not due to non-functioning Aδ or C fibers. ns, $p = 0.49$ (CDT) and $p = 0.08$ (WDT); unpaired t-test with Welch's correction. Error bars indicate median ± interquartile range. (**b**) Nerve conduction velocity testing. Amplitudes in the upper limb were normal and conduction velocities (CV) were within normal range. Amplitudes were lower in the legs, with CV slightly under normal values. This could have possibly led to slower NWR latencies, but it seems unlikely that mild motor CV impairment leads to a complete NWR abolishment. **ADM**, abductor digiti minimi. Detailed statistics are available in Supplementary Data 1. Source data are provided as a Source Data file.

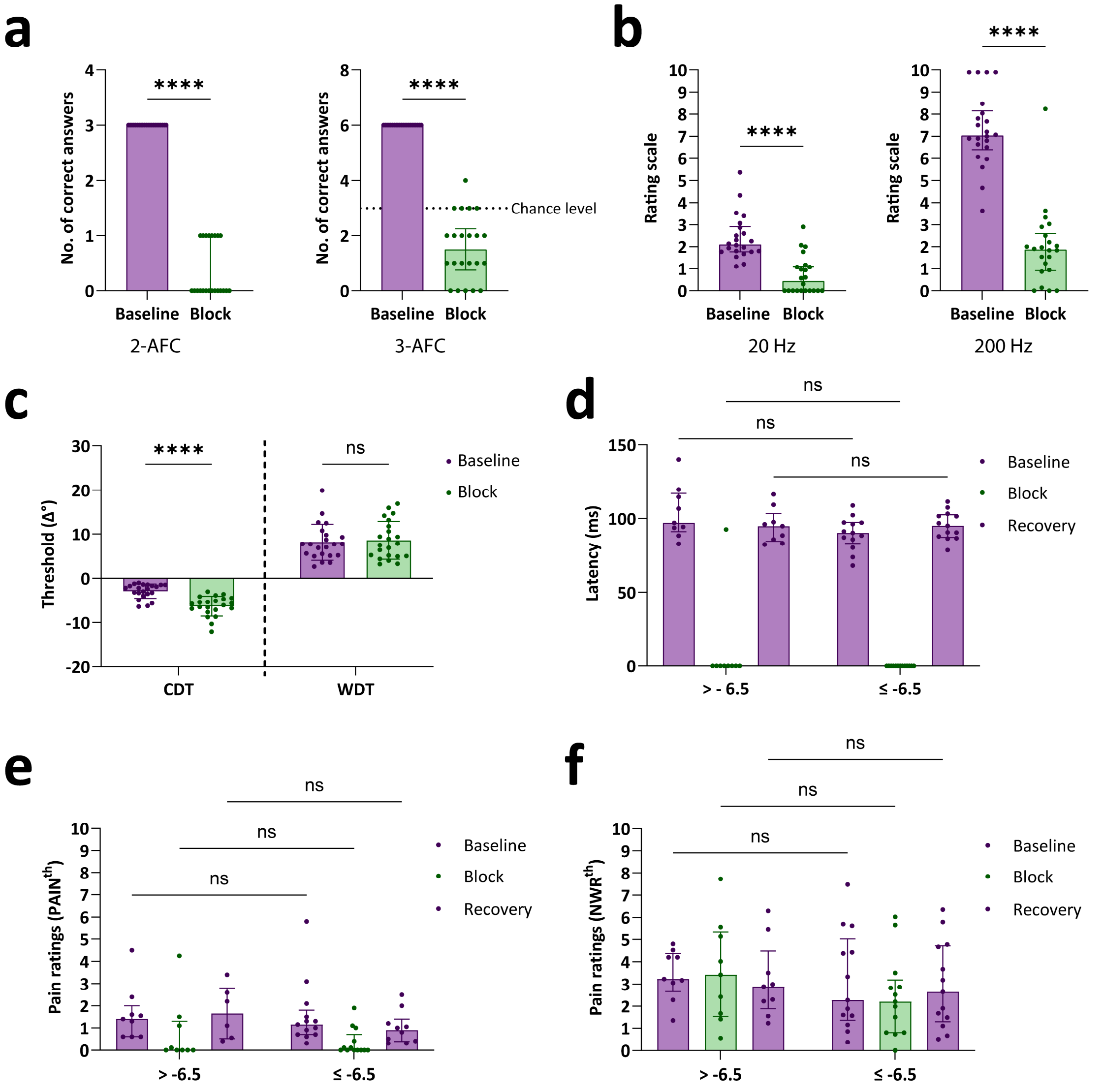

Figure S7. Verification of nerve block in healthy control subjects (HCs). (**a**) Two-alternative forced choice (2-AFC) task (*left*). Eight out of 22 HCs had one correct answer (out of three) in the 2-AFC task during the nerve block, in sharp contrast to the baseline condition (Baseline: 3.00 (3.00), Block 0.00 (1.00), p<0.0001, Wilcoxon test). Three-alternative forced choice (3-AFC) task (*right*). Although a spread was seen in the 3-AFC task, there was a statistically significant impairment compared to baseline (Baseline: 6.00 (0.00), Block: 1.50 (1.50), p<0.0001, Wilcoxon test). (**b**) Intensity rating tasks. Vibration ratings were significantly lower during nerve block for both 20 Hz (*left*) and 200 Hz (r*ight*) (20 Hz: Baseline 2.10 (1.15), Block 0.43 (1.09); 200Hz: Baseline 7.03 (1.75), Block 1.87 (1.68); ****, p<0.0001, Wilcoxon test). (**c**) Temperature detection thresholds. Cold detection threshold (ΔCDT) decreased during nerve block while warm detection threshold (WDT) remained unchanged between conditions (CDT: Baseline -2.33 (-2.17), Block -5.88 (-2.61), p<0.0001. WDT: Baseline 7.69 (4.86), Block 7.67 (7.09), p=0.68, Wilcoxon test). **(d-f)** Comparisons of **(f)** reflex latency and pain ratings at **(e)** pain threshold (PAINth) and at **(f)** nociceptive withdrawal reflex threshold (NWRth) between HCs showing affected CDT during nerve

block (ΔCDT >-6.5°C) and HCs that showed no change in CDT (ΔCDT ≤-6.5°C). No differences were found between the groups (ns, $p > 0.05$; two-way repeated measures ANOVA in **d** and **f**; mixed-effects model in **e**; followed by Šídák's multiple comparisons test). Error bars in all panels indicate median ± interquartile range. Detailed statistics are available in Supplementary Data 1. Source data are provided as a Source Data file.

Supplementary Note 1

On conduction velocities of A-nociceptors in humans

Adriaensen et al. (1983) reported A fiber nociceptors conducting in the Aδ velocity range, in apparent contradiction to the Aβ range conduction velocities (CVs) observed in Nagi et al (2019). However, Adriaensen reported a mean CV of ~19 m/s for all myelinated afferents in the radial nerve, with the fastest units below 35 m/s, and similar CVs for low- and high-threshold afferents. In that study, recordings were obtained at the wrist, implying relatively short conduction distances to many hand receptive fields. By contrast, radial nerve microneurography studies using more proximal recording sites (e.g., near the elbow) commonly report faster A-fiber velocities, with mean values above 40 m/s and upper values approaching 60 m/s (Bhuiyan et al. 2025; Nagi et al. 2019). The basis for this difference is not entirely clear; however, CV estimates over short conduction paths may be influenced by delays at the terminal or branch level, which represent a larger fraction of the measured latency when conduction distances are short. Regardless of the cause, the study by Adriaensen et al. (1983) is in agreement with those of Nagi et al. (2019) and Bhuyian et al. (2025) in that the CVs of A-nociceptors are not dissimilar from those of low-threshold mechanoreceptor fibers. Nevertheless, it should be noted that few microneurography recordings of Aδ fibers have been published, and as morphological observations confirm the existence of an appreciable Aδ-fiber population in humans (e.g., Schellens et al. 1993), this may reflect technical limitations of microneurography compared with, for instance, teased nerve fiber recordings as employed in non-human primates (Treede et al. 1998). Thus, the paucity of published microneurography recordings of human nociceptors conducting in the Aδ range does not preclude the existence of such fibers, and whereas convergent findings in the present study point to a dominant role for Aβ nociceptors in mechanical pain and reflexive behavior, other as yet unavailable approaches may be required to fully resolve this question.

Supplementary Table 1. Primary antibodies

Antigen	Host, isotype	Supplier	Cat #	Lot #	Dilution
**CGRP**	Guinea pig	Synaptic Systems	414 004	1-5	1:1000
**CGRP**	Rabbit	Bachem (Peninsula)	T-4031	A03398	1:2000
**GFP (mCitrine)**	Rabbit	Life Technologies	A11122	939306	1:200
**GFP (mCitrine)**	Chicken	Abcam	ab13970	GR236651	1:500
**GFP (mCitrine)**	Chicken	Aves Labs	GFP-1020	N/A	1:2000
**MBP**	Mouse IgG_1	Santa Cruz Biotechnology	sc-271524	A1224	1:200
**NFH**	Chicken	Thermo Fisher Scientific	PA1-10002	YH4025641	1:1000
**pERK**	Rabbit	Cell Signaling Technology	4370	15	1:200
**PKCγ**	Guinea pig	Frontier Institute	PKCg-GP-Af350	N/A	1:1000
**S100β**	Rabbit	Proteintech	15146-1-AP	00134531	1:200
**TRPV1**	Rabbit	Synaptic Systems	444 033	1-1	1:1000
**VGluT3**	Mouse IgG_{2a}	Synaptic Systems	135 211	135211/1	1:500

N/A, not provided by supplier.

Supplementary Table 2. Secondary antibodies

Host, target	Conjugate	Supplier	Cat #	Lot #
**Goat α-chicken IgY (H+L)**	Alexa Fluor 488	Life Technologies	A32931	WF325106
**Goat α-chicken IgY (H+L)**	Alexa Fluor 555	Life Technologies	A21437	2720395
**Goat α-guinea pig IgG (H+L)**	Alexa Fluor 647	Life Technologies	A21450	1378366
**Goat α-mouse IgG_1**	Alexa Fluor 555	Life Technologies	A21127	2384708
**Goat α-mouse IgG_{2a}**	Alexa Fluor 647	Life Technologies	A21241	2366136
**Goat α-rabbit IgG (H+L)**	Alexa Fluor 488	Life Technologies	A11034	2861864
**Goat α-rabbit IgG (H+L)**	Alexa Fluor Plus 555	Life Technologies	A32732	YA361054
**Goat α-rabbit IgG (H+L)**	Alexa Fluor 647	Life Technologies	A21245	1445259
**Goat α-rabbit IgG (H+L)**	Alexa Fluor 750	Life Technologies	A21039	2652949

References

Adriaensen H, Gybels J, Handwerker HO, Van Hees J (1983) Response properties of thin myelinated (A-delta) fibers in human skin nerves. J Neurophysiol 49 (1):111-122. doi:10.1152/jn.1983.49.1.111

Bhuiyan SA, Nagi SS, Sankaranarayanan I, Semizoglou E, Usoskin D, Yang L, Yu H, Arendt-Tranholm A, Bertels Z, Bhatia P, Bouchatta O, Boyer K, Cervantes A, Chalif J, Chintalapudi H, Cicalo A, Copits B, Cronin C, Curatolo M, Dong X, Dougherty PM, Dourson A, Funk G, Gabriel K, Griesemer DS, Guo H, Gupta P, Hofstetter C, Horton P, Hsieh A, Inturi NN, Jain A, Jayakar S, Johnston B, Kim R, Krauter D, Kupari J, Lemen J, Lesnak JB, Liu W, Lopez I, Lu Y, MacMillan HJ, Mazhar K, Meriau P, Moffitt JR, Moreno MM, Mwirigi JM, Naz H, O'Brein J, Payne M, Del Rosario J, Rosen SF, Shiers S, Simpson E, Slivicki R, Stone JR, Tavares-Ferreira D, Uhelski M, Woolf CJ, Xu Q, Yi J, Yousuf MS, Zhu D, Cavalli V, Zhao G, Olausson H, Ernfors P, Gereau RW, Luo W, Price TJ, Renthal W, Network NPHP (2025) A reference atlas of the human dorsal root ganglion. bioRxiv:2025.2011.2005.686654. doi:10.1101/2025.11.05.686654

Nagi SS, Marshall AG, Makdani A, Jarocka E, Liljencrantz J, Ridderstrom M, Shaikh S, O'Neill F, Saade D, Donkervoort S, Foley AR, Minde J, Trulsson M, Cole J, Bonnemann CG, Chesler AT, Bushnell MC, McGlone F, Olausson H (2019) An ultrafast system for signaling mechanical pain in human skin. Sci Adv 5 (7):eaaw1297. doi:10.1126/sciadv.aaw1297

Schellens RLLA, van Veen BK, Gabreëls-Festen AAWM, Notermans SLH, van't Hof MA, Stegeman DF (1993) A statistical approach to fiber diameter distribution in human sural nerve. Muscle Nerve 16 (12):1342-1350. doi:https://doi.org/10.1002/mus.880161212

Treede R-D, Meyer RA, Campbell JN (1998) Myelinated mechanically insensitive afferents from monkey hairy skin: heat-response properties. Journal of Neurophysiology 80 (3):1082-1093. doi:10.1152/jn.1998.80.3.1082
