# Supplementary material for: Central role for fast nociceptors in mechanical nocifensive behavior and sensitization
Source: Nat Commun. 2026 Jul 25;17:7271. doi: 10.1038/s41467-026-75948-z (PMC13401607; doi:10.1038/s41467-026-75948-z)
Supplement: Supplementary file 2 — Description of Additional Supplementary Files [file 41467_2026_75948_MOESM2_ESM.pdf]

Description of Additional Supplementary Files

Supplementary Data 1. Detailed statistics.

Supplementary Movie 1. Optogenetic stimulation of the plantar hindpaw of an NFH;NaV1.8;ReaChR mouse induced a rapid unilateral withdrawal reflex with a latency of 23 ms from the start of the light pulse (5 ms, 460 nm). The video was recorded at 1000 fps and slowed down 20x for visualization. To increase the visibility of paw movement a long-pass red filter (610 nm) was used to block the light from the fiber optic cable. To visualize the stimulus an infrared light was lit at the start of the pulse and remained lit for the duration of the trial.

Supplementary Movie 2. Optogenetic stimulation of the plantar hindpaw of an NFH;NaV1.8;ReaChR mouse induced a rapid unilateral withdrawal reflex with a latency of 22 ms from the start of the light pulse (5 ms, 460 nm). The mouse also showed a transient orbital tightening, guarding of the paw, and attended the paw. The video was recorded at 1000 fps and slowed down 20x for visualization. To increase the visibility of paw movement a long-pass red filter (610 nm) was used to block the light from the fiber optic cable. To visualize the stimulus an infrared light was lit at the start of the pulse and remained lit for the duration of the trial.

Supplementary Movie 3. Optogenetic stimulation of the plantar hindpaw of a litter mate control mouse did not induce paw withdrawal nor other behavioral response. The mouse was heterozygous for $Nefh^{CreERT2}$ and LSL_FSF_ReaChR but negative for the $NaV1.8^{FlpO}$ allele, and received tamoxifen injection as its triple heterozygous NFH;NaV1.8;ReaChR litter mates. The video was recorded at 1000 fps and slowed down 20x for visualization. To increase the visibility of paw movement a long-pass red filter (610 nm) was used to block the light from the fiber optic cable. To visualize the stimulus an infrared light was lit at the start of the pulse and remained lit for the duration of the trial.
