# Supplementary material for: Central role for fast nociceptors in mechanical nocifensive behavior and sensitization
Source: Nat Commun. 2026 Jul 25;17:7271. doi: 10.1038/s41467-026-75948-z (PMC13401607; doi:10.1038/s41467-026-75948-z)
Supplement: Supplementary file 7 — Reporting Summary [file 41467_2026_75948_MOESM7_ESM.pdf]

nature portfolio

Corresponding author(s): Max Larsson
Last updated by author(s): Jul 2, 2026

Reporting Summary

Nature Portfolio wishes to improve the reproducibility of the work that we publish. This form provides structure for consistency and transparency in reporting. For further information on Nature Portfolio policies, see our Editorial Policies and the Editorial Policy Checklist.

Statistics

For all statistical analyses, confirm that the following items are present in the figure legend, table legend, main text, or Methods section.

n/a	Confirmed	
☐	☒	The exact sample size (*n*) for each experimental group/condition, given as a discrete number and unit of measurement
☐	☒	A statement on whether measurements were taken from distinct samples or whether the same sample was measured repeatedly
☐	☒	The statistical test(s) used AND whether they are one- or two-sided<br>*Only common tests should be described solely by name; describe more complex techniques in the Methods section.*
☐	☒	A description of all covariates tested
☐	☒	A description of any assumptions or corrections, such as tests of normality and adjustment for multiple comparisons
☐	☒	A full description of the statistical parameters including central tendency (e.g. means) or other basic estimates (e.g. regression coefficient) AND variation (e.g. standard deviation) or associated estimates of uncertainty (e.g. confidence intervals)
☐	☒	For null hypothesis testing, the test statistic (e.g. *F*, *t*, *r*) with confidence intervals, effect sizes, degrees of freedom and *P* value noted<br>*Give P values as exact values whenever suitable.*
☒	☐	For Bayesian analysis, information on the choice of priors and Markov chain Monte Carlo settings
☒	☐	For hierarchical and complex designs, identification of the appropriate level for tests and full reporting of outcomes
☒	☐	Estimates of effect sizes (e.g. Cohen's *d*, Pearson's *r*), indicating how they were calculated

Our web collection on statistics for biologists contains articles on many of the points above.

Software and code

Policy information about availability of computer code

Data collection	LAS X v4.8.0 (Leica)<br>Zen 3.1 (Zeiss)<br>ImageJ/µManager 2.0 (micro-manager.org)<br>RADIUS 2.1 (EMSIS)<br>WinWCP (Dr. J Dempster, University of Strathclyde)<br>ANY-maze 6.33 (Stoelting)<br>Open Broadcaster Software 27.0.1 (obsproject.com)<br>EasyPS2000 v2.05 (Elektro-Automatik)<br>PowerLab 16/35 (AD Instruments)<br>LabChart v8.1.16 (AD Instruments)<br>Synergy CareFusion EDX 20.0 (CareFusion)
Data analysis	GraphPad Prism 10.5 (GraphPad)<br>Fiji/ImageJ v1.54p (NIH)<br>Clampfit 11.2 (Molecular Devices)<br>Matlab R2021b (MathWorks)<br>The Observer XT 15 (Noldus)<br>Kinovea 2023.1.2 (kinovea.org)

For manuscripts utilizing custom algorithms or software that are central to the research but not yet described in published literature, software must be made available to editors and reviewers. We strongly encourage code deposition in a community repository (e.g. GitHub). See the Nature Portfolio guidelines for submitting code & software for further information.

Data

Policy information about availability of data

All manuscripts must include a data availability statement. This statement should provide the following information, where applicable:

- Accession codes, unique identifiers, or web links for publicly available datasets
- A description of any restrictions on data availability
- For clinical datasets or third party data, please ensure that the statement adheres to our policy

All quantitative data underlying statistical analyses are available in the Source Data file. Because of the large size of the raw image and video files, data were not deposited in a public repository. All data are available from the corresponding authors upon request. Source data are provided with this paper.

Research involving human participants, their data, or biological material

Policy information about studies with human participants or human data. See also policy information about sex, gender (identity/presentation), and sexual orientation and race, ethnicity and racism.

Reporting on sex and gender	The sex of the human subjects was noted but was not considered in the analysis.
Reporting on race, ethnicity, or other socially relevant groupings	Ethnicity, race or other socially relevant groupings were not considered.
Population characteristics	The healthy participants, of which 19 were females and 10 males, were between 18 and 40 years old, except for the age-matched control (a 77-year old female). The Aβ deafferented individual was a 70-year old male.
Recruitment	Healthy participants were recruited from an existing database at the Center for Social and Affective Neuroscience (Linköping University) and advertisements on social media sites. Self-selection biased the cohort towards a younger age range less likely to be subject to age-related somatosensory deficits, and for this reason an age-matched control was used for comparison with the Aβ deafferented individual. Written informed consent was obtained from all individuals before the start of the experiment.
Ethics oversight	The study was approved by the ethics committee of Linköping University.

Note that full information on the approval of the study protocol must also be provided in the manuscript.

Field-specific reporting

Please select the one below that is the best fit for your research. If you are not sure, read the appropriate sections before making your selection.

[x] Life sciences   [ ] Behavioural & social sciences   [ ] Ecological, evolutionary & environmental sciences

For a reference copy of the document with all sections, see nature.com/documents/nr-reporting-summary-flat.pdf

Life sciences study design

All studies must disclose on these points even when the disclosure is negative.

Sample size	Statistical methods were not used to predetermine sample sizes. For animal behavior assays, we used sample sizes similar to those previously widely used to successfully detect meaningful group differences in related studies: for von Frey/chemogenetic inhibition see e.g. https://doi.org/10.1002/art.40118, for optogenetics/real time place preference see e.g. https://doi.org/10.1016/j.neuron.2017.07.024. For optogenetics facial expression analysis we used a similar number of animals as in our previous study (Le Moëne and Larsson, https://doi.org/10.1523/ENEURO.0349-22.2022). For morphological data, observations were generally from four or more but at least three individual animals, in accordance with common practice and based on the assumption that these morphological features show little variability between individual inbred animals. In the human experiments, sample size was limited by the fact that only one Aβ deafferented individual is currently known and alive. The number of healthy participants was similar to what was used by us in a recent related study https://doi.org/10.1371/journal.pone.0309048. For these experiments any meaningful effects would, if present, be large and evident in a very small sample.
Data exclusions	No data from animal experiments were excluded. In the human experiments, six out of 29 healthy control subjects were excluded from the analysis because of incompletely collected data or a lack of detectable NWR response.
Replication	All animal experiments were reliably replicated in several individual animals derived from multiple litters and consistent with pilot experiments. Human experimental data were partly based on intra-individual replicates because of the single Aβ deafferented individual. All

animal and human experiments were successfully replicated.

Randomization

Where applicable, animals were randomly assigned to different treatment groups (e.g. which animals received saline or chemogenetic agonist, or in which order). Collected tissue samples were not part of separate experimental groups and randomization therefore not relevant. Human healthy participants were not divided into separate experimental groups and randomization therefore not relevant.

Blinding

For animal behavioral assays, the experimenter was blind to treatment (vehicle vs chemogenetic agonist, and TeTxLC vs control AAV), and when possible also for genotype. However, because behavioral responses of NFH;NaV1.8;ReaChR mice to optogenetic stimulation were obvious whereas control litter mates did not respond to the optogenetic light, it was not possible for the experimenter to be blind to genotype in these instances. Similarly, in vivo imaging, ex vivo optogenetics/electrophysiology and morphological analysis were not blinded to genotype. In addition, it was not practically possible to blind the behavioral sensitization experiments with respect to stimulated versus non-stimulated hind paws, as the experimenter performed both the sensitization step and the behavioral testing. Because of the very robust differences detected and reliable replication by multiple experimenters (for the behavioral assays, including the sensitization experiments), we do not expect this to have meaningfully affected the analysis. The human experiments were not possible to blind because the Aβ-deafferented individual was previously known to the experimenters and tested in separation; we do not expect this to have effected the analysis of the human data because of the very substantial differences between the Aβ-deafferented individual and the control subjects, including the age-matched control.

Reporting for specific materials, systems and methods

We require information from authors about some types of materials, experimental systems and methods used in many studies. Here, indicate whether each material, system or method listed is relevant to your study. If you are not sure if a list item applies to your research, read the appropriate section before selecting a response.

Materials & experimental systems

n/a	Involved in the study
☐	☒ Antibodies
☒	☐ Eukaryotic cell lines
☒	☐ Palaeontology and archaeology
☐	☒ Animals and other organisms
☒	☐ Clinical data
☒	☐ Dual use research of concern
☒	☐ Plants

Methods

n/a	Involved in the study
☒	☐ ChIP-seq
☒	☐ Flow cytometry
☒	☐ MRI-based neuroimaging

Antibodies

Antibodies used

anti-CGRP, guinea pig, Synaptic Systems, cat# 414 004, lot# 1-5
anti-CGRP, rabbit, Bachem (Peninsula Labs), cat# T-4031, lot# A03398
GFP, rabbit, Life Technologies, cat# A11122, lot# 939306
GFP, chicken, Abcam, cat# ab13970, lot# GR236651
GFP, chicken, Aves Labs, cat# GFP-1020, lot# not provided by supplier
MBP, mouse, Santa Cruz Biotechnology, cat# sc-271524, lot# A1224
NFH, chicken, Thermo Fisher Scientific, cat# PA1-10002, lot# YH4025641
pERK, rabbit, Cell Signaling Technology, cat# 4370, lot# 15
PKCγ, guinea pig, Frontier Institute, cat# PKCg-GP-Af350, lot# not provided by supplier
S100β, Proteintech, cat# 15146-1-AP, lot# 00134531
TRPV1, rabbit, Synaptic Systems, cat# 444 033, lot# 1-1
VGluT3, mouse, Synaptic Systems, cat# 135 211, lot# 135211/1
Goat α-chicken IgY (H+L) Alexa Fluor 488, Life Technologies, cat# A32931, lot# WF325106
Goat α-chicken IgY (H+L) Alexa Fluor 555, Life Technologies, cat# A21437, lot# 2720395
Goat α-guinea pig IgG (H+L) Alexa Fluor 647, Life Technologies, cat# A21450, lot# 1378366
Goat α-mouse IgG1 Alexa Fluor 555, Life Technologies, cat# A21127, lot# 2384708
Goat α-mouse IgG2a Alexa Fluor 647, Life Technologies, cat# A21241, lot# 2366136
Goat α-rabbit IgG (H+L) Alexa Fluor 488, Life Technologies, cat# A11034, lot# 2861864
Goat α-rabbit IgG (H+L) Alexa Fluor Plus 555, Life Technologies, cat# A32732, lot# YA361054
Goat α-rabbit IgG (H+L) Alexa Fluor 647, Life Technologies, cat# A21245, lot# 1445259
Goat α-rabbit IgG (H+L) Alexa Fluor 750, Life Technologies, cat# A21039, lo# 2652949

Validation

The guinea pig CGRP antibody produces in our hands identical labeling patterns in mouse spinal cord, skin, peripheral nerve and dorsal root ganglia as other well-tested CGRP antibodies, including the rabbit CGRP antibody used here. The rabbit anti-CGRP antibody has been validated by the manufacturer by ELISA and have been shown by us to selectively label dense core vesicles as expected in primary afferent terminals by immunogold electron microscopy (e g https://doi.org/10.1111/j.1460-9568.2005.04081.x). The GFP antibodies, when detected using red-fluorescent secondary antibodies, labeled the same structures as the endogenous mCitrine fluorescence. The MBP antibody was validated by the manufacturer by Western blot, shows the expected labeling pattern in peripheral nerve tissue and co-localizes with other markers of myelinated fibers (e g S100β) in peripheral tissue. The NFH antibody has been validated by the manufacturer to detect phosphorylated NFH using Western blot. We and others (eg http://

pubmed.ncbi.nlm.nih.gov/pubmed/34735549) have also observed selective labeling by this antibody of myelinated primary afferent fibers using myelination markers (MBP, S100β) as well as labeling patterns in the CNS consistent with neurofilament staining. The pERK antibody has been validated by the manufacturer using Western blot and is very widely used (9352 total citations, 742 citations for immunocyto-/histochemical applications); in addition, we detect no or very sparse labeling with this antibody in the spinal cord of naïve animals, or in the spinal cord contralateral to peripheral stimulation as shown in this study. The PKCγ antibody has been validated by the manufacturer by Western blot and produces the expected labeling pattern in the spinal cord. The TRPV1 antibody has been validated by the manufacturer using Western blot and produces in our hands and by the manufacturer the expected labeling pattern in the spinal cord. The VGluT3 antibody has been validated by us to co-label the same structures as two other VGluT3 antibodies (https://pubmed.ncbi.nlm.nih.gov/30783617/) and shows the expected distribution in the spinal cord.

Animals and other research organisms

Policy information about studies involving animals; ARRIVE guidelines recommended for reporting animal research, and Sex and Gender in Research

Laboratory animals	We used the following mouse strains:<br>NFHCreERT2 (https://www.nature.com/articles/s41598-025-95874-2)<br>NaV1.8FlpO (https://doi.org/10.3389/fnmol.2025.1574219)<br>R26-LSL-FSF-ReaChR-mCitrine (JAX #024846, here called ReaChR mice)<br>ROSA26DR-Matrix-dAPEX2 (JAX #032764; here called APEX2 mice)<br>Ai195 (JAX #034112; here called GCaMP7s)<br>RC::FPDi (JAX #029040; here called hM4Di)<br>The mice were used for experiments between 10 and 35 weeks of age. All crosses used were on mixed 129S x C57Bl/6 background.
Wild animals	The study did not involve wild animals.
Reporting on sex	In all experimental groups we sought to balance the sex ratio as evenly as possible and continuously monitored for possible sex differences. No such differences were found, although experimental groups when further divided by sex were generally too small to perform statistical analyses on such data. As sexual dimorphism was not a major focus of the study, we deemed it not justifiable with respect to animal use and resources to increase experimental group sizes enough to perform such analyses in the absence of observed tendencies towards any sex differences.
Field-collected samples	The study did not involve samples collected from the field.
Ethics oversight	The animal experiments were approved by the Animal Ethics Committees at Linköping University or Uppsala tingsrätt.

Note that full information on the approval of the study protocol must also be provided in the manuscript.

Plants

Seed stocks	*Report on the source of all seed stocks or other plant material used. If applicable, state the seed stock centre and catalogue number. If plant specimens were collected from the field, describe the collection location, date and sampling procedures.*
Novel plant genotypes	*Describe the methods by which all novel plant genotypes were produced. This includes those generated by transgenic approaches, gene editing, chemical/radiation-based mutagenesis and hybridization. For transgenic lines, describe the transformation method, the number of independent lines analyzed and the generation upon which experiments were performed. For gene-edited lines, describe the editor used, the endogenous sequence targeted for editing, the targeting guide RNA sequence (if applicable) and how the editor was applied.*
Authentication	*Describe any authentication procedures for each seed stock used or novel genotype generated. Describe any experiments used to assess the effect of a mutation and, where applicable, how potential secondary effects (e.g. second site T-DNA insertions, mosiacism, off-target gene editing) were examined.*
