# Supplementary material for: Central role for fast nociceptors in mechanical nocifensive behavior and sensitization
Source: Nat Commun. 2026 Jul 25;17:7271. doi: 10.1038/s41467-026-75948-z (PMC13401607; doi:10.1038/s41467-026-75948-z)
Supplement: Supplementary file 8 — Transparent Peer Review File [file 41467_2026_75948_MOESM8_ESM.pdf]

nature portfolio

Peer Review File

Central role for fast nociceptors in mechanical nocifensive behavior and sensitization

Corresponding Author: Dr Max Larsson

This file contains all reviewer reports in order by version, followed by all author rebuttals in order by version.

Version 0:

Reviewer comments:

Reviewer #1

(Remarks to the Author)
In this manuscript, the authors develop a novel genetic approach to access mechanically responsive A-nociceptors (A-MNs) in mice and demonstrate their causal link to pain and nocifensive behaviors in both mice and humans. By crossing NFH (neurofilament heavy chain) CreER mice with Nav1.8 Flp mice, they validate the high specificity and selectivity of this intersectional strategy for targeting A-MNs co-expressing NFH and Nav1.8. Through manipulation of these neurons, the authors convincingly show that A-MNs are not only necessary and sufficient for rapid mechanical nociceptive withdrawal reflexes (NWRs) in mice, but also contribute to the affective-motivational dimensions of pain, as evidenced by place aversion assays and changes in facial expression.
Overall, this work constitutes a comprehensive and technically impressive study that dissects the role of A-MNs in nocifensive behavior and pain perception. I have only minor suggestions to further strengthen the conclusions:
1. Given that Nav1.8-expressing neurons are also implicated in cold pain (PMID: 18669863), the authors should examine whether the identified A-MNs respond to cold stimuli and contribute to cold-related nociception.
2. The observation that prolonged activation of A-MNs induces mechanical allodynia and central sensitization suggests these neurons may play a role in pain sensitization following injury. To directly test this hypothesis, the authors need to silence these neurons in models such as spared nerve injury (SNI) or CFA-induced inflammation, and assess the impact on mechanical allodynia.
3. Electrophysiological and in vivo imaging data indicate that the major component of A-MNs defined by NFH and Nav1.8 co-expression corresponds to Aδ fibers. Clarifying how these fibers in mice relate to human Aβ fibers would help contextualize the translational relevance of the findings.
4. The human case presented is compelling, but limited to a single subject. It would be helpful to explicitly acknowledge the limitation of n = 1 while also emphasizing the rarity of the condition.

Reviewer #2

(Remarks to the Author)
A. Key Results with Significance
This study addresses the functional role of myelinated A-fiber mechano-nociceptors (A-MNs) in pain behavior, nociceptive withdrawal reflexes, and sensitization. Key findings: (1) Optogenetic activation of A-MNs is sufficient to induce rapid withdrawal reflexes (~21 ms latency), while chemogenetic or synaptic silencing impairs mechanical withdrawal thresholds; (2) A-MNs terminate densely in laminae I-IIo and form glomerular synapses receiving inhibitory input; (3) Selective A-MN stimulation induces both peripheral and central sensitization; (4) A human individual lacking A-beta fibers shows complete absence of mechanical withdrawal reflexes despite intact pain perception. The authors use a novel intersectional genetic approach (NFH-CreERT2 x NaV1.8-FlpO) to target the broad A-MN population, combined with optogenetics, chemogenetics, calcium imaging, electron microscopy, and human psychophysics. A very thorough study.
This study addresses a significant gap in the pain field: We really don't know much about A-MN sensory neurons in comparison to nociceptors and mechanoreceptors. They are a very critical subpopulation of sensory neurons. Thus this study will shape our understanding of nociception and pain. The human validation substantially elevates this work also.
Major key limitation that can be addressed in revision: the precise distinction between A-delta and A-beta nociceptors relies on conduction velocity measurements that require additional methodological clarification (see comments below)

B. Validity

Point #1 (Electrophysiology): Figure 2B shows conduction velocities with a wide spread. The distinction between A-delta and A-beta neurons is not clearly justified - some neurons near the boundary could reasonably be classified as either. The dashed line at 10 m/s appears to indicate a cutoff but this is not explained in the figure legend? Further statistical analysis to support the clustering could strengthen this classification.
Point #2 (Electrophysiology): Figure S1 shows the ReaChR latency correction experiment but includes only a few example traces. More raw data should be presented, ideally as a table showing each recording with its calculated latency. Additionally, all example traces shown appear to be in the C-fiber range - an example of a light-stimulated A-beta neuron trace would strengthen the claim that A-beta neurons were successfully recorded.
Point #3 (Anatomy): Figure 2A images are small and it is difficult to assess how axon diameter was measured. The use of CGRP to assess axon diameter may not be the best, perhaps a marker like tubulin or neurofilament H that fills the axon would be more appropriate. Alternatively, co-staining with a myelin marker would help distinguish myelinated (A-fiber) from unmyelinated (C-fiber) populations.
Point #4 (Anatomy): Figure 3A shows skin innervation but one arrowhead in glabrous skin resembles a Meissner corpuscle ending. An S100-beta co-stain would help rule out labeling of Meissner afferents, which would be off-target for this study.

C. Data and Methodology Clarifications
The ages for some experiments (EM, von Frey assay) were mentioned, but what were the ages of the mice for the other experiments?
How was the distance for the conduction velocity calculation determined?
How did you determine the cutoffs for C-fibers, A-deltas, and A-betas? The C-fiber cluster is pretty obvious but the slowest neuron in the A-betas appears that it could be an A-delta?
Secondary antibodies were not included in the antibody table.

D. Analytical Approach
Statistical tests were well detailed and the appropriate tests were used for the data types. P-values were reported.
E. Suggested Improvements
MAJOR Suggestions
1. Conduction velocity calculation methodology: The methodology used to calculate conduction velocity should be explicitly stated and ideally shown with example recordings (maybe in the figure?). This is critical since the classification of neurons as A-beta nociceptors is central to the paper's claims. For example, Figure S1 needs additional data: More raw traces could be shown. A summary table of all recorded neurons with their calculated latencies would strengthen confidence in the conduction velocity measurements.
2. Framing for broad readership: For a diverse audience, the results section would benefit from more context explaining why each experiment was performed. Currently, results are presented sequentially without sufficient framing of the scientific questions being addressed. In particular, the lamina innervation section (Figure 4) should explain the functional significance of superficial vs. deep termination patterns for readers unfamiliar with spinal cord organization.
3. Von Frey filament context: The authors should reference literature establishing which force thresholds (e.g., 8g, 26g) are considered nociceptive versus innocuous, to help readers interpret the calcium imaging results in Figure 2C.
4. Figure 2B: The dashed line at 10 m/s is not explained in the figure legend. Please clarify what this indicates (presumably the A-delta/A-beta cutoff). Consider displaying conduction velocities as a histogram to better show the distribution.
5. Figure 1D: The color scheme for mCitrine differs from the rest of the paper, making comparison difficult. Consider using consistent colors throughout. The overlapping histogram bars also make it hard to see the Scn10a+ population at larger cross-sectional areas - separating these into distinct panels or using transparency would help.
6. Figure 3: Consider adding an S100-beta co-stain to confirm that labeled endings in glabrous skin are free nerve endings rather than Meissner corpuscles.
7. Figure 8: The sensitization experiments feel disconnected from the preceding affective pain section. Consider reframing this section to explain how prolonged nociceptor activation relates to chronic pain mechanisms, which would strengthen the narrative flow.
Minor Suggestions

Figure 4: The authors speculate that superficial lamina innervation corresponds to glabrous skin afferents while deeper innervation corresponds to hairy skin. A simple CTB injection experiment into glabrous vs. hairy skin could test this hypothesis and would strengthen the paper.8. Secondary antibodies used for immunohistochemistry should be listed in the methods or reagent table.
Figure 6F: The representation of facial expression data is somewhat confusing. Additional example images or supplementary videos would help readers interpret these results.
Figure 3: Titrating tamoxifen to label single neurons and quantifying receptive field sizes (number of hair follicles innervated) would allow comparison with published data on CGRP+ circumferential endings.
Figure 6G: The dotted line at zero should be labeled (e.g., "no preference") for clarity

F. Clarity and Context
1. Light stimulation correction: Please clarify how the ReaChR channel kinetics were corrected to calculate conduction velocity. In our experience, light stimulation produces longer latencies than electrical stimulation of the same neuron due to the time required for channel opening and depolarization. More detail on this correction, with additional raw traces in Figure S1, would strengthen confidence in the A-beta classification.
2. Audience considerations: The manuscript would benefit from additional context explaining: (a) why the lamina termination patterns matter functionally (superficial = anterolateral pathway/emotion vs. deep = reflex coordination); (b) what distinguishes nociceptive von Frey forces from innocuous touch; (c) why the pERK/PKC-gamma results in Figure 8 are significant for understanding chronic pain. Currently, experiments are presented without sufficient explanation of the

questions being addressed.
3. Narrative structure: The Results section reads as a sequential list of experiments rather than a coherent story. Consider restructuring to better motivate each experimental question for non-specialist readers. The introduction could better frame why an intersectional genetic approach targeting the broad A-MN population advances beyond previous subpopulation studies.
4. Figure 5 (APEX ultrastructure): This is an elegant experiment showing ultrastructural organization at A-MN terminals. The significance for understanding sensory gating in the dorsal horn could be emphasized more in the results/discussion.

Reviewer #3

(Remarks to the Author)
I co-reviewed this manuscript with one of the reviewers who provided the listed reports. This is part of the Nature Communications initiative to facilitate training in peer review and to provide appropriate recognition for Early Career Researchers who co-review manuscripts.

Reviewer #4

(Remarks to the Author)
In this manuscript an intersectional genetic approach was used to isolate A-fiber nociceptors combining the expression of Nav1.8 and NFH in mice. The authors present functional data to support a role of A-nociceptors for rapid mechanical noxious withdrawal reflexes and live-cell imaging and structural data on skin endings and spinal cord connectivity using electron microscopy and in situ hybridization/immunohistochemistry.

Major concerns:
The intersectional genetic approach resulted in a mixed population concerning fiber class (A delta & A beta nociceptors) and functional class with mechanical activation thresholds ranging from 0.6g to pin prick. It remains unclear whether the heat stimulation took into account the preference of AMH nociceptors for rapid or prolonged heating (also note the high thermal thresholds of some AMH that can be sensitized in a burn 1) – thus, information about heat sensitivity might be limited. No information is given on cold sensitivity. The structural data on epidermal vs. circumferential endings and the CGRP+ vs. CGRP- patterns support the view of a mixed population. Optogenetic activation provoked a convincing nociceptive response supporting the nociceptive nature of at least part of the population.
The authors present human data in a translational attempt that shows reduced pin-prick pain and absence of a mechanically induced withdrawal reflex. This should suggest A-beta mechanonociceptors are crucial for mechanically induced withdrawal reflexes and pinprick pain. In humans. The authors claim that A-beta mechano-nociceptors are the only known type of A-fiber mechanonociceptors (line 238). That is puzzling as the role of A-delta mechanonociceptors in humans is well established including direct microneurography recordings 2. It is even more puzzling that pain ratings to suprathreshold pin-prick stimuli increase and pin-prick pain thresholds decrease in humans when A-fiber conduction is differentially blocked in healthy volunteers, suggesting a major role of C-mechanonociceptors for pin-prick pain 3. Thus, it appears that the result in the individuum lacking A-beta fibers do not fit the expected results based on healthy volunteers. Moreover, the contribution of the human data to the mouse results is unclear.
In summary, the authors succeeded to generate a mouse that provides an interesting tool to study a population of different A-fiber nociceptors. Unfortunately, the exact nature of functional nociceptor classes in the animal model remains unclear. Moreover, the translational attempt is of limited value, not only based on species differences, but also because of the focus on A-beta nociceptors that does not match the mix of A-beta and A-delta in the mouse model.

Specific comments:

Line 217 Increase in mechanical withdrawal threshold using chemogenetics – v.Frey thresholds increased with hot plate test unchanged (but laser evoked pain was not tested - is the lack of heat nocifensive behaviour due to the slow heating ramp 4

Paragraph human – lines 233 to 249
The authors provide data from a human individual with selective Abeta deafferentiation, but presumably intact A-delta nociceptors. This individual had no mechanically induced withdrawal reflex and higher mechanical pain thresholds. However, it is important to note that in healthy humans, pain ratings to pin-prick stimuli increase upon A-fiber block indicating that the net effect of low and high thresholds A-fiber input on intensity ratings (in contrast to withdrawal thresholds in animal pain behaviour) appears to be analgesic (mechanical pinprick pain sensitivity (z>1) and mechanical pinprick pain thresholds (z>2) as assessed by QST) 3. It also important to note that this is the only attempt in this manuscript to differentiate A-beta and A-delta nociceptors – the intersectional approach in the mice resulted in a combined A-beta A-delta population and thus does not allow for such a differentiation.

Line 260 reduced light intensity might indicate peripheral of central sensitization as the response is elicited with input from fewer fibers – prolonged activation of nociceptors is expected to lead to adaptation rather than sensitization thus, the arguments for a peripheral sensitization are not convincing to me.

Line 333-336 It is obvious, that based on conduction velocity rapid reflexes require A-fibers. It is unclear why the data would imply that behavioural responses after C-mechanonociceptor activation cannot be spinally mediated. Also note that most of the C-mechanonociceptors are polymodal – heat pain threshold with slow temperature increase is a function of polymodal nociceptors and leads to a spinal withdrawal reflex.

Line 340-342 It is important to note that the strategy used resulted in a broad spectrum of A-beta / A-delta fibers with mechanical activation thresholds between 0.6 g and pinprick – thus, it appears inadequate to regard the NFH+/NAv1.8 fibers as one population.

Literatur

1. Meyer RA and Campbell JN. Myelinated nociceptive afferents account for the hyperalgesia that follows a burn to the hand. Science 1981; 213: 1527–1529.
2. Adriaensen H, Gybels J, Handwerker HO, et al. Response properties of thin myelinated (A-delta) fibers in human skin nerves. JNeurophysiol 1983; 49: 111–122.
3. Vollert J, Magerl W, Baron R, et al. Pathophysiological mechanisms of neuropathic pain: comparison of sensory phenotypes in patients and human surrogate pain models. Pain 2018; 159: 1090–1102. 2018/03/02. DOI: 10.1097/j.pain.0000000000001190.
4. Yeomans DC and Proudfit HK. Nociceptive responses to high and low rates of noxious cutaneous heating are mediated by different nociceptors in the rat: electrophysiological evidence. Pain 1996; 68: 141–150. DOI: 10.1016/S0304-3959(96)03177-6.

Version 1:

Reviewer comments:

Reviewer #1

(Remarks to the Author)
The authors have successfully addressed my concerns. I have not more questions.

Reviewer #2

(Remarks to the Author)
The authors have addressed all of our concerns. Congratulations on a beautiful and impactful study.

Reviewer #3

(Remarks to the Author)
I co-reviewed this manuscript with one of the reviewers who provided the listed reports. This is part of the Nature Communications initiative to facilitate training in peer review and to provide appropriate recognition for Early Career Researchers who co-review manuscripts.

Reviewer #4

(Remarks to the Author)
The revision has markedly improved the paper.
However, I have still two major concerns:

1) The authors added data on a “peripheral nerve block of Abeta fiber” or “ Abeta-preferential block”:
Figure S7a suggests that there is a preferential block of A-beta fibers as compared to the less pronounced effect on A-delta cold fibers S7c. However, it appears that vibration ratings to the 200 Hz stimulus were reduced from 7 to 2 and cold detection thresholds worsened from around 3 degrees centigrade to 2.5 to 5.5°C. Thus, if a cold stimulus would have been 5.5°C colder that skin temperature, 50% of the volunteers would not have felt it (compared to 4 of 22 that gave a zero rating to the 200 Hz vibration stimulus. From my perspective, the test paradigms do not allow to draw firm conclusions on Abeta vs Adelta preference of the block.
It is important to note that the actual key experiment, i.e. the effect of an A-fiber block on pinprick-pain vs. cold and tactile sensation has already been published2. An A-fiber pressure block indeed blocks cold and tactile sensation while warmth detection is unchanged – however, the loss of first pain responses to pinprick – the very parameter that is mediated by A-fiber mechanonociceptors – showed exactly the same time course of the block as compared to the cold and tactile sensation. Thus, this direct evidence suggests that the time course of impaired A-beta and A-delta function following a pressure nerve block are both following the impaired first prinprick pain in humans-
[The absolute reaction time had a clear peak between 0.25 and 0.3 seconds – assuming a reaction time of 0.15s the remaining 0.1 to 0.15 s would be inline with conduction velocities of 10m/s i.e. in the Adelta range).

2) The differentiation between A-beta and A-delta fibers in humans
Bhuiyan and Nagi acknowledge that thinly myelinated Adelta fibers are “rarely encountered” in human microneurography. (there have been speculations that the minute tip of the electrode need to be close a node of Ranvier in A-delta fibers to produce a sufficient signal and thus, minute movements will lead to a loss of the recording in contrast to C and A-beta fibers). In monkey, teased fiber techniques can be used that allow for stable recordings of A-delta fibers. These recording result in a clear bimodal distribution of A-fiber conduction velocity (A-delta peak between 10 and 15 m/s; A beta peak between 40 and 45m/s) 4. A corresponding bimodal distribution is also found in the diameter of myelinated axons in human sural nerve 1

(peaks around 4 and 8 µm) – thus, it appears that there is a lack of functional data on human A-delta fibers. Even though there is obvious variability of conduction velocities -for example, skin temperature differences are a major source as seen by a lower conduction velocity of human A-fibers in the distal as compared to the proximal phalangeal segment with a difference of more than 10 m/s 3 – however, the clear bimodal distribution of axonal diameter in human sensory nerves and the clear bimodal distribution of conduction velocities in myelinated fibers in the monkey suggests that we do have a lack of information on single fiber function of A-delta fibers by microneurography.

In summary, I would suggest to downtown the claim, that (first) pin-prick pain is mediated by A-beta fibers and acknowledge that there is a certain lack of functional data on human A-delta fibers potentially linked to limitations of the microneurography recording technique as compared to teased fiber recordings.

[1] Dyck PJ, Karnes J, Sparks M, Low PA. The morphometric composition of myelinated fibres by nerve, level and species related to nerve microenvironment and ischaemia. Electroencephalogr.Clin Neurophysiol.Suppl. 1982; 36:39–55.
[2] Henrich F, Magerl W, Klein T, Greffrath W, Treede RD. Capsaicin-sensitive C- and A-fibre nociceptors control long-term potentiation-like pain amplification in humans. Brain. 2015; 138:2505–20.
[3] Mackel R. Conduction of neural impulses in human mechanoreceptive cutaneous afferents. J Physiol. 1988; 401:597–615.
[4] Treede RD, Meyer RA, Campbell JN. Myelinated mechanically insensitive afferents from monkey hairy skin: heat-response properties. J Neurophysiol. 1998; 80:1082–93.

Open Access This Peer Review File is licensed under a Creative Commons Attribution 4.0 International License, which permits use, sharing, adaptation, distribution and reproduction in any medium or format, as long as you give appropriate credit to the original author(s) and the source, provide a link to the Creative Commons license, and indicate if changes were made.
In cases where reviewers are anonymous, credit should be given to 'Anonymous Referee' and the source.
The images or other third party material in this Peer Review File are included in the article's Creative Commons license, unless indicated otherwise in a credit line to the material. If material is not included in the article's Creative Commons license and your intended use is not permitted by statutory regulation or exceeds the permitted use, you will need to obtain permission directly from the copyright holder.
To view a copy of this license, visit https://creativecommons.org/licenses/by/4.0/

Response to referees

Reviewer #1 (Remarks to the Author):

In this manuscript, the authors develop a novel genetic approach to access mechanically responsive A-nociceptors (A-MNs) in mice and demonstrate their causal link to pain and nocifensive behaviors in both mice and humans. By crossing NFH (neurofilament heavy chain) CreER mice with Nav1.8 Flp mice, they validate the high specificity and selectivity of this intersectional strategy for targeting A-MNs co-expressing NFH and Nav1.8. Through manipulation of these neurons, the authors convincingly show that A-MNs are not only necessary and sufficient for rapid mechanical nociceptive withdrawal reflexes (NWRs) in mice, but also contribute to the affective-motivational dimensions of pain, as evidenced by place aversion assays and changes in facial expression.
Overall, this work constitutes a comprehensive and technically impressive study that dissects the role of A-MNs in nocifensive behavior and pain perception. I have only minor suggestions to further strengthen the conclusions:
1. Given that Nav1.8-expressing neurons are also implicated in cold pain (PMID: 18669863), the authors should examine whether the identified A-MNs respond to cold stimuli and contribute to cold-related nociception.
Response: We thank the reviewer for the comment. We have now conducted a new set of in vivo DRG imaging experiments to assess responses to lower and higher temperatures (new Fig. S2). We found that only 4 % of A-MNs responded to either cold temperatures down to 10°C, or to heat temperatures up to 55°C, and that 5 % of cells responded to cold/heat but not to mechanical stimuli. Note that our thermal device did not reach below 10°C, and because some A mechano-cold nociceptors may have a threshold below this temperature (PMID 11287480) it is possible that some mechano-cold (or pure cold) receptors were not identified as such in our experiments.

2. The observation that prolonged activation of A-MNs induces mechanical allodynia and central sensitization suggests these neurons may play a role in pain sensitization following injury. To directly test this hypothesis, the authors need to silence these neurons in models such as spared nerve injury (SNI) or CFA-induced inflammation, and assess the impact on mechanical allodynia.
Response: Thank you for the comment. Assessing the involvement of myelinated nociceptors in inflammatory or neuropathic models of allodynia is certainly of very high interest. However, teasing out the roles of these fibers in the induction versus expression/maintenance of different forms of allodynia requires a large-scale effort involving several sets of experiments silencing/unsilencing A-MNs at different timepoints that we expect would take a significant time to complete (at least 9-12 months) and feel is outside the scope of the present study.

3. Electrophysiological and in vivo imaging data indicate that the major component of A-MNs defined by NFH and Nav1.8 co-expression corresponds to Aδ fibers. Clarifying how these fibers in mice relate to human Aβ fibers would help contextualize the translational relevance of the findings.
Response: Thank you for the comment. We have added a paragraph on this in the Discussion as follows:
"In humans, A-MNs generally form Aβ fibers as evidenced from microneurography[49] (see Supplementary Note 1); by contrast, single-fiber recordings has indicated a mixed Aδ/Aβ population in mice, with some A-MN fibers having CVs above 10 m/s, above the CV range of D-

hair Aδ-LTMRs and overlapping with that of Aβ-LTMRs in this species[3, 35]. In accordance with this, we observed that optogenetic stimulation of $NFH^+/Na_V1.8^+$ fibers triggered both Aδ and Aβ responses. Moreover, soma size, which shows a positive correlation with CV in rodents[17, 36], was similar between targeted neurons in NFH;Na_V1.8;ReaChR mice and non-targeted NFH^+, presumed A-LTMR, neurons. Notably, among $NFH^+/Na_V1.8^+$ fibers, those with undetectable CGRP were considerably larger than those expressing this peptide, suggesting that A-MNs with Aβ fibers in the mouse are preferentially non-peptidergic.“

4. The human case presented is compelling, but limited to a single subject. It would be helpful to explicitly acknowledge the limitation of n = 1 while also emphasizing the rarity of the condition.
Response: Thank you for the comment. We have now further emphasized the rarity of this condition but importantly also included a large set of new data on Aβ-preferential nerve block in healthy individuals that complement and substantially strengthen the conclusions inferred from the observations in the single Aβ-deafferented individual.

Reviewer #2 (Remarks to the Author):

A. Key Results with Significance
This study addresses the functional role of myelinated A-fiber mechano-nociceptors (A-MNs) in pain behavior, nociceptive withdrawal reflexes, and sensitization. Key findings: (1) Optogenetic activation of A-MNs is sufficient to induce rapid withdrawal reflexes (~21 ms latency), while chemogenetic or synaptic silencing impairs mechanical withdrawal thresholds; (2) A-MNs terminate densely in laminae I-IIo and form glomerular synapses receiving inhibitory input; (3) Selective A-MN stimulation induces both peripheral and central sensitization; (4) A human individual lacking A-beta fibers shows complete absence of mechanical withdrawal reflexes despite intact pain perception. The authors use a novel intersectional genetic approach (NFH-CreERT2 x NaV1.8-FlpO) to target the broad A-MN population, combined with optogenetics, chemogenetics, calcium imaging, electron microscopy, and human psychophysics. A very thorough study.
This study addresses a significant gap in the pain field: We really don’t know much about A-MN sensory neurons in comparison to nociceptors and mechanoreceptors. They are a very critical subpopulation of sensory neurons. Thus this study will shape our understanding of nociception and pain. The human validation substantially elevates this work also.
Major key limitation that can be addressed in revision: the precise distinction between A-delta and A-beta nociceptors relies on conduction velocity measurements that require additional methodological clarification (see comments below)

B. Validity
Point #1 (Electrophysiology): Figure 2B shows conduction velocities with a wide spread. The distinction between A-delta and A-beta neurons is not clearly justified - some neurons near the boundary could reasonably be classified as either. The dashed line at 10 m/s appears to indicate a cutoff but this is not explained in the figure legend? Further statistical analysis to support the clustering could strengthen this classification.
Response: Thank you for the comment. Although the boundary between Aβ and Aδ fibers is not clear-cut, we use 10 m/s as a cut-off as suggested by Koltzenburg et al (PMID:9325353) and Djouhri and Lawson (PMID: 15464202). We have now added an inter-group statistical analysis showing that optogenetically induced responses are different from electrically induced C fiber

responses, and that electrically induced Aβ fiber responses are different from Aδ responses. The figure 2B and the legend have been updated accordingly.

Point #2 (Electrophysiology): Figure S1 shows the ReaChR latency correction experiment but includes only a few example traces. More raw data should be presented, ideally as a table showing each recording with its calculated latency. Additionally, all example traces shown appear to be in the C-fiber range - an example of a light-stimulated A-beta neuron trace would strengthen the claim that A-beta neurons were successfully recorded.
Response: Thank you for the comment. We have added the data for the ReaChR activation latency for individual cells as a plot to Figure S1c. Additionally, both Figure 2 and Figure S1 have now been updated with indicators to improve the clarity of conduction velocity measurements.

Point #3 (Anatomy): Figure 2A images are small and it is difficult to assess how axon diameter was measured. The use of CGRP to assess axon diameter may not be the best, perhaps a marker like tubulin or neurofilament H that fills the axon would be more appropriate. Alternatively, co-staining with a myelin marker would help distinguish myelinated (A-fiber) from unmyelinated (C-fiber) populations.
Response: Thank you for the comment. A good point. We have now replaced the analysis in Fig. 2a with an analysis of sciatic nerve co-immunolabeled for mCitrine, CGRP, and myelin basic protein (MBP) as a myelin sheath marker. We measured the total fiber diameter including the myelin sheath of mCitrine+ fibers with and without CGRP; we excluded unmyelinated CGRP+ fibers from this analysis as it makes less sense to compare the diameter of these with total myelinated fiber diameters. This new analysis substantially strengthened the conclusion that A-MNs without CGRP are larger than those with the peptide.

Point #4 (Anatomy): Figure 3A shows skin innervation but one arrowhead in glabrous skin resembles a Meissner corpuscle ending. An S100-beta co-stain would help rule out labeling of Meissner afferents, which would be off-target for this study.
Response: Thank you for the comment. The varicosities established by some mCitrine+ fibers are within the epidermis (not subjacent to it, like Meissner corpuscles) and too small (~1-3 μm) to be Meissner corpuscles. To rule out innervation of Meissner corpuscles, we have now co-immunolabeled with S100β, showing that mCitrine+ fibers, while coursing through dermal papillae and adjacent epidermis near Meissner corpuscles, do not actually enter the corpuscles. Example micrographs are shown in a new Figure S2 (note re-numbering of the supplementary figures – the old Fig. S2 is now Fig. S4).

C. Data and Methodology Clarifications
The ages for some experiments (EM, von Frey assay) were mentioned, but what were the ages of the mice for the other experiments?
Response: Thank you for pointing out this omission – we have now added the ages of the mice to the description of all experiments.
How was the distance for the conduction velocity calculation determined?
Response: Thank you for the comment. The distance was measured from the edge of DRG to the recording pipette, which has now been indicated more clearly in Figure 2.
How did you determine the cutoffs for C-fibers, A-deltas, and A-betas? The C-fiber cluster is pretty obvious but the slowest neuron in the A-betas appears that it could be an A-delta?
Response: Thank you for the comment. We determined the cutoffs based on both the distribution of our data and the publication Djouhri et al. (2004), where the cutoff for Aβ/Aδ was 10 m/s, and the lowest Aβ we measured was 10.3 m/s.
Secondary antibodies were not included in the antibody table.

Response: Thank you for the comment – we have added a new table with secondary antbodies.

D. Analytical Approach
Statistical tests were well detailed and the appropriate tests were used for the data types. P-values were reported.
E. Suggested Improvements
MAJOR Suggestions
1. Conduction velocity calculation methodology: The methodology used to calculate conduction velocity should be explicitly stated and ideally shown with example recordings (maybe in the figure?). This is critical since the classification of neurons as A-beta nociceptors is central to the paper's claims. For example, Figure S1 needs additional data: More raw traces could be shown. A summary table of all recorded neurons with their calculated latencies would strengthen confidence in the conduction velocity measurements.
Response: Thank you for the comment. Now, we have added the methodology used to calculate the conduction velocity in the method section. Accordingly, the Figure 2 and Figure S1 have also been updated with clear indications to clarify the method, and a summary plot of all measured ReaChR activation delays has now been added to Figure S1.
2. Framing for broad readership: For a diverse audience, the results section would benefit from more context explaining why each experiment was performed. Currently, results are presented sequentially without sufficient framing of the scientific questions being addressed. In particular, the lamina innervation section (Figure 4) should explain the functional significance of superficial vs. deep termination patterns for readers unfamiliar with spinal cord organization.
Response: Thank you for the comment. We have now added an introductory passage in the Results section with respect to spinal terminations:

" The superficial dorsal horn (Rexed's laminae I and dorsal lamina II) is considered the major target site of nociceptive primary afferent fibers and a core site of nociceptive signal processing[37]. By contrast, the deeper dorsal horn, including the ventralmost lamina II to V, are generally thought to mainly receive terminations from low-threshold mechanoreceptors (LTMRs)[37-40]. Notably, although this region also contains neurons that receive nociceptive input, such input is thought to mainly occur at synapses onto these neurons' dorsally directed dendrites in the superficial dorsal horn, or via polysynaptic routes[37, 39]. However, morphological reconstructions of intracellularly recorded afferent fibers in mice have suggested that some myelinated A-nociceptors arborize throughout the dorsal horn, including laminae III-V[8]. To resolve this discrepancy, we examined the terminations of mCitrine$^+$ fibers in the spinal cord of NFH;Na$_V$1.8;ReaChR mice.";

We also added an introductory passage to the section on sensitization:

"The role of C fiber nociceptors in the induction of pain hypersensitivity is well-established, and these fibers are widely considered to be the primary inducers of central and peripheral sensitization in tissue injury and inflammation[51, 52]. By contrast, whether A-nociceptor activation can induce pain hypersensitivity and central sensitization is unknown. As a first step to investigate this, we tested whether strong, prolonged selective stimulation of NFH$^+$/Na$_V$1.8$^+$ fibers could induce sensitization."

3. Von Frey filament context: The authors should reference literature establishing which force thresholds (e.g., 8g, 26g) are considered nociceptive versus innocuous, to help readers interpret the calcium imaging results in Figure 2C.

Response: Thank you for the comment; we have now expanded on this in the Results section, with appropriate references.

4. Figure 2B: The dashed line at 10 m/s is not explained in the figure legend. Please clarify what this indicates (presumably the A-delta/A-beta cutoff). Consider displaying conduction velocities as a histogram to better show the distribution.

Response: Thank you for the comment. The dashed line at 10m/s is the cutoff of A-beta/A-delta and now we have updated the figure legend. We have also changed the scatter plot in Fig. 2b to a histogram.

5. Figure 1D: The color scheme for mCitrine differs from the rest of the paper, making comparison difficult. Consider using consistent colors throughout. The overlapping histogram bars also make it hard to see the Scn10a+ population at larger cross-sectional areas - separating these into distinct panels or using transparency would help.

Response: Thank you for the suggestions. We have now harmonized the color scheme to make it easier to read (green is now used for sample groups where mCitrine+ fibers or (in humans) Aβ fibers have been visualized or manipulated). We have also separated the histograms in Fig. 1d.

6. Figure 3: Consider adding an S100-beta co-stain to confirm that labeled endings in glabrous skin are free nerve endings rather than Meissner corpuscles.

Response: See Point #4 above. We have now added a co-staining with S100β to a new Fig. S3.

7. Figure 8: The sensitization experiments feel disconnected from the preceding affective pain section. Consider reframing this section to explain how prolonged nociceptor activation relates to chronic pain mechanisms, which would strengthen the narrative flow.

Response: Thank you for the comment. We have now revised this section to better emphasize the rationale for these experiments using an introductory passage as shown above and a concluding remark: "These observations suggest the possibility that A-MNs may have a larger and more instructive role in pain hypersensitivity produced by tissue injury than previously envisioned".

Minor Suggestions

Figure 4: The authors speculate that superficial lamina innervation corresponds to glabrous skin afferents while deeper innervation corresponds to hairy skin. A simple CTB injection experiment into glabrous vs. hairy skin could test this hypothesis and would strengthen the paper.

Response: Thank you for the comment. To be clear, we do not claim that innervation in superficial and deep laminae correspond to glabrous and hairy skin innervation, respectively, but rather that the innervation in the ventral part of lamina II that we only observed in the medial part of the dorsal horn possibly reflects glabrous skin innervation. This was based on the fact that glabrous skin innervation terminates medially in the dorsal horn of the lumbar enlargement, something that is well-established in rats based on tracer studies (most comphrehensively studied in PMID 12761822). In mice this has been less studied, but C-LTMRs, which are not found in glabrous skin, are fully excluded from the medial dorsal horn in the lumbar enlargement (see e.g. our recent study PMID 40528874), strongly supporting a similar pattern as in rats. We have now performed transganglionic tracing experiments using CTB injected into hind paw glabrous skin in NFH;NaV1.8;ReaChR mice, showing that the dense innervation of mCitrine^{+} fibers in medial lamina II co-localizes with terminations from glabrous skin afferents. We also find that the cervical enlargement shows a similar pattern in medial lamina II of spinal segments with fore paw input. In addition, we have immunolabeled for VGluT3 as a marker of C-LTMRs, showing that C-LTMR terminals, the presence of which marks dorsal horn regions with hairy skin input, are located laterally to (and never overlaps with) the region of dense NFH/NaV1.8 fiber innervation. We have made a new Fig. S4 describing these observations.

8. Secondary antibodies used for immunohistochemistry should be listed in the methods or reagent table.
Response: See above; this has now been added.
Figure 6F: The representation of facial expression data is somewhat confusing. Additional example images or supplementary videos would help readers interpret these results.
Response: Thank you for the comment. We have now added a new supplementary Fig. S3 showing examples of optogenetically induced facial expression changes; see also Supplementary Video 2.
Figure 3: Titrating tamoxifen to label single neurons and quantifying receptive field sizes (number of hair follicles innervated) would allow comparison with published data on CGRP+ circumferential endings.
Response: Thank you for the comment. As our NFH/NaV1.8 mice target a broad population of fibers, using low tamoxifen doses to achieve sparse labeling would stochastically target different subpopulations in different individual mice, with little control over which specific populations would be targeted in different regions of the skin in different mice. Even if we were able to find skin regions with sparse labeling of circumferental nerve endings and quantify receptive field sizes of these, it is unclear to us that this would achieve useful insight that is not better studied in other models.
Figure 6G: The dotted line at zero should be labeled (e.g., "no preference") for clarity
Response: Thank you for the suggestion; we have now added the label "No preference" to the figure.

F. Clarity and Context
1. Light stimulation correction: Please clarify how the ReaChR channel kinetics were corrected to calculate conduction velocity. In our experience, light stimulation produces longer latencies than electrical stimulation of the same neuron due to the time required for channel opening and depolarization. More detail on this correction, with additional raw traces in Figure S1, would strengthen confidence in the A-beta classification.
Response: Thank you for the comment. We have added extra indicators in both Figure 2 and Figure S1 to clarify how the conduction velocity of light stimulation was corrected with ReaChR channel kinetics.
2. Audience considerations: The manuscript would benefit from additional context explaining: (a) why the lamina termination patterns matter functionally (superficial = anterolateral pathway/emotion vs. deep = reflex coordination); (b) what distinguishes nociceptive von Frey forces from innocuous touch; (c) why the pERK/PKC-gamma results in Figure 8 are significant for understanding chronic pain. Currently, experiments are presented without sufficient explanation of the questions being addressed.
Response: Thank you for the suggestions. We have added more context in the Results and Discussion with respect to these issues.
3. Narrative structure: The Results section reads as a sequential list of experiments rather than a coherent story. Consider restructuring to better motivate each experimental question for non-specialist readers. The introduction could better frame why an intersectional genetic approach targeting the broad A-MN population advances beyond previous subpopulation studies.
Response: Thank you for the comment. We have addressed this as above and also included a statement in the introduction on the rationale for targeting a broad population, as follows: "We chose to target a broad population of A-nociceptors to uncover a comprehensive picture of the integrated functional role of these fibers, rather than the more subtle roles afforded by smaller subpopulations."

4. Figure 5 (APEX ultrastructure): This is an elegant experiment showing ultrastructural organization at A-MN terminals. The significance for understanding sensory gating in the dorsal horn could be emphasized more in the results/discussion.
Response: Thank you for the comment. We have now included a sentence on this in the Discussion: "Moreover, our electron microscopic observations that many A-MN terminals form synaptic glomeruli, occasionally receiving presynaptic presumed inhibitory synaptic contacts, suggest that signals from these fibers may be subject to complex processing even at the first synapse, possibly including gating from other sensory input[59]"

Reviewer #3 (Remarks to the Author):

I co-reviewed this manuscript with one of the reviewers who provided the listed reports. This is part of the Nature Communications initiative to facilitate training in peer review and to provide appropriate recognition for Early Career Researchers who co-review manuscripts.

Reviewer #4 (Remarks to the Author):

In this manuscript an intersectional genetic approach was used to isolate A-fiber nociceptors combining the expression of Nav1.8 and NFH in mice. The authors present functional data to support a role of A-nociceptors for rapid mechanical noxious withdrawal reflexes and live-cell imaging and structural data on skin endings and spinal cord connectivity using electron microscopy and in situ hybridization/immunohistochemistry.

Major concerns:
The intersectional genetic approach resulted in a mixed population concerning fiber class (A delta & A beta nociceptors) and functional class with mechanical activation thresholds ranging from 0.6g to pin prick. It remains unclear whether the heat stimulation took into account the preference of AMH nociceptors for rapid or prolonged heating (also note the high thermal thresholds of some AMH that can be sensitized in a burn 1) – thus, information about heat sensitivity might be limited. No information is given on cold sensitivity.
Response: Thank you for the comment. We have now included a new set of in vivo imaging experiments using a cold ramp to 10°C and a heating ramp to 55°C. In these experiments, we observed very few A-MN cells that responded to heating up to 55°C or to cooling down to 10°C, indicating that a large majority of A-MNs captured in our mice were selectively mechanoreceptive. However, as our thermal device did not reach below 10°C, and because some A mechano-cold nociceptors may have a lower threshold than this (PMID 11287480), it is possible that some mechano-cold receptors were not identifed as cold responsive in our experiments.

The structural data on epidermal vs. circumferential endings and the CGRP+ vs. CGRP- patterns support the view of a mixed population. Optogenetic activation provoked a convincing nociceptive response supporting the nociceptive nature of at least part of the population.
The authors present human data in a translational attempt that shows reduced pin-prick pain and absence of a mechanically induced withdrawal reflex. This should suggest A-beta mechanonociceptors are crucial for mechanically induced withdrawal reflexes and pinprick pain. In humans. The authors claim that A-beta mechano-nociceptors are the only known type

of A-fiber mechanonociceptors (line 238). That is puzzling as the role of A-delta mechanonociceptors in humans is well established including direct microneurography recordings 2.
Response: Thank you for the comment. We have now added a discussion on this in a Supplementary Note. In short, Adriaensen et al. showed A-nociceptors conducting at Aδ velocities; however, also A-LTMRs recorded in that study conducted in the Aδ range, not different from the A-nociceptors. Thus, we believe that the differences in CVs between that study and Nagi et al (2019) and Bhuiyan et al (bioRrxiv 2025) may be attributed to technical differences.

It is even more puzzling that pain ratings to suprathreshold pin-prick stimuli increase and pin-prick pain thresholds decrease in humans when A-fiber conduction is differentially blocked in healthy volunteers, suggesting a major role of C-mechanonociceptors for pin-prick pain 3. Thus, it appears that the result in the individuum lacking A-beta fibers do not fit the expected results based on healthy volunteers. Moreover, the contribution of the human data to the mouse results is unclear.
Response: Thank you for the comment. We have now added new results from human individuals subjected to Aβ-preferential nerve block. We found that nerve block substantially decreased pain ratings to stimuli applied at pain threshold force, but not to stimuli applied at the higher reflex threshold force. This is fully in line with the observations of increased pain threshold in the Aβ-deafferented invididual, but also indicates that C-mechanonociceptors are recruited at higher forces, both in the Aβ-deafferented individual and in the healthy individuals subjected to nerve block. We hope that these additional data makes the relevance of the human part of the study clearer.
In summary, the authors succeeded to generate a mouse that provides an interesting tool to study a population of different A-fiber nociceptors. Unfortunately, the exact nature of functional nociceptor classes in the animal model remains unclear.
Response: Thank you for the comment. The aim of the present study was to make an integrated investigation into the function and anatomy of A-nociceptors as a whole, not to examine a homogeneous subpopulation. We have added text to better convey the rationale for this in the Introduction.
Moreover, the translational attempt is of limited value, not only based on species differences, but also because of the focus on A-beta nociceptors that does not match the mix of A-beta and A-delta in the mouse model.
Response: Thank you for the comment. In our view, we are studying similar populations in mice and humans. While in mice it is clear that many nociceptors form Aδ fibers, we believe that human orthologues of these rather conduct in the Aβ range (Bhuiyan et al, bioRrxiv 2025). This is also apparent for A-LTMRs, where D-hair receptors in mice form Aδ fibers whereas we do not observe Aδ-LTMRs in humans (Bhuiyan et al, bioRrxiv 2025). Thus, the fact that mouse and human A-nociceptors partly conduct at different relative velocities does not mean that the broad populations studied here in humans and mice are not composed of comparable orthologous subpopulations. We have added a section on this in the Discussion: "In humans, A-MNs generally form Aβ fibers as evidenced from microneurography[49] (see Supplementary Note 1); by contrast, single-fiber recordings has indicated a mixed Aδ/Aβ population in mice, with some A-MN fibers having CVs above 10 m/s, above the CV range of D-hair Aδ-LTMRs and overlapping with that of Aβ-LTMRs in this species[3, 35]."

Specific comments:

Line 217 Increase in mechanical withdrawal threshold using chemogenetics – v.Frey thresholds increased with hot plate test unchanged (but laser evoked pain was not tested - is the lack of heat nocifensive behaviour due to the slow heating ramp 4

Response: Thank you for the comment. The hot plate test did not involve a heat ramp but was set at a constant temperature of 50°C. We thus do not believe that the lack of an effect was due to inadequate inhibition of of A-MNs, but more likely to the retained function of C-nociceptors. However, it is certainly possible that a CO_2 laser evoked pain assay, which has a faster action than the hot plate, could uncover a role for A-mechanoheat receptors in heat nocifensive behavior.

Paragraph human – lines 233 to 249

The authors provide data from a human individual with selective Abeta deafferentiation, but presumably intact A-delta nociceptors. This individual had no mechanically induced withdrawal reflex and higher mechanical pain thresholds. However, it is important to note that in healthy humans, pain ratings to pin-prick stimuli increase upon A-fiber block indicating that the net effect of low and high thresholds A-fiber input on intensity ratings (in contrast to withdrawal thresholds in animal pain behaviour) appears to be analgesic (mechanical pinprick pain sensitivity (z>1) and mechanical pinprick pain thresholds (z>2) as assessed by QST) 3. It also important to note that this is the only attempt in this manuscript to differentiate A-beta and A-delta nociceptors – the intersectional approach in the mice resulted in a combined A-beta A-delta population and thus does not allow for such a differentiation.

Response: Thank you for the comment. As we argue that A-MNs in humans conduct in Aβ range (see above, and Bhuiyan et al, bioRrxiv 2025), we believe the Aβ deafferented individual relies on C fibers to sense mechanical pain. To complement our observations from this individual, we have now also included a new set of data from human healthy individuals subjected to Aβ-preferential nerve block. These new observations align with those from the Aβ-deafferented individual, in that such nerve block abolished mechanical withdrawal reflexes and reduced mechanical pain perception. The nerve block data is provided in a revised Fig. 7 along with the data from the Aβ-deafferented individual.

Line 260 reduced light intensity might indicate peripheral of central sensitization as the response is elicited with input from fewer fibers – prolonged activation of nociceptors is expected to lead to adaptation rather than sensitization thus, the arguments for a peripheral sensitization are not convincing to me.

Response: Thank you for the comment. We devised the stimulation paradigm to broadly mimic the time course of the activation of capsaicin-sensitive fibers in the intradermal capsaicin model of pain. In this model mechano-insensitive C fibers fire for several min after capsaicin injection in humans (PMID 10686178). Importantly, the same C fibres become sensitized to mechanical stimuli. In rats, intradermal capsaicin also induces ERK phosphorylation in TRPV1+ primary afferents, mediating thermal hyperalgesia (PMID 12196597). Thus, we believe it is entirely possible that prolonged activation of A-MNs would sensitize these fibers rather than desensitize them. Moreover, the illuminated area in our experiments were of the same size regardless of intensity and we would presumably activate a similar number of afferents. Thus we argue that the most likely explanation for the results is that heightened excitability after prolonged stimulation means that fewer channelrhodopsin molecules need to be activated in order to induce firing and a reflex.

Line 333-336 It is obvious, that based on conduction velocity rapid reflexes require A-fibers. It is

unclear why the data would imply that behavioural responses after C-mechanonociceptor activation cannot be spinally mediated. Also note that most of the C-mechanonociceptors are polymodal – heat pain threshold with slow temperature increase is a function of polymodal nociceptors and leads to a spinal withdrawal reflex.

Response: Thank you for the comment. We argue that the very long latency (>500 ms) of the withdrawal induced by MrgprpD+ C fibers, which are C-mechanonociceptors not activated by heat below 50°C, suggest that this action is not mediated by spinal reflexes. Heat-induced reflexes by polymodal C fibers can certainly be (and likely are) mediated by such reflexes. We have now clarified that it is withdrawal induced by MrgprD+ fibers specifically that we believe are not mediated by spinal circuits alone. While we agree with the reviewer that it is clear that A fibers must underlie mechanical withdrawal reflexes, we believe that it is important to discuss MrgprD+ fibers in this context as it is commonly stated in the literature that these fibers mediate mechanical withdrawal reflexes.

Line 340-342 It is important to note that the strategy used resulted in a broad spectrum of A-beta / A-delta fibers with mechanical activation thresholds between 0.6 g and pinprick – thus, it appears inadequate to regard the NFH+/NAv1.8 fibers as one population.

Response: Thank you for the comment. We are not regarding NFH+/NaV1.8+ as a single homogeneous population, but a broad population of fast-conducting nociceptors that is clearly separate from C fiber nociceptors. In this study we aimed to provide an integrative functional and morphological view of this population, in particular in relation to mechanical nociception and in contrast to C nociceptors. Hopefully this comes through better in the revised text, including with the clarified rationale for targeting this broad population that we added in the Introduction: "We chose to target a broad population of A-nociceptors to uncover a comprehensive picture of the integrated functional role of these fibers, rather than the more subtle roles afforded by smaller subpopulations."

Literatur

1. Meyer RA and Campbell JN. Myelinated nociceptive afferents account for the hyperalgesia that follows a burn to the hand. Science 1981; 213: 1527–1529.
2. Adriaensen H, Gybels J, Handwerker HO, et al. Response properties of thin myelinated (A-delta) fibers in human skin nerves. JNeurophysiol 1983; 49: 111–122.
3. Vollert J, Magerl W, Baron R, et al. Pathophysiological mechanisms of neuropathic pain: comparison of sensory phenotypes in patients and human surrogate pain models. Pain 2018; 159: 1090–1102. 2018/03/02. DOI: 10.1097/j.pain.0000000000001190.
4. Yeomans DC and Proudfit HK. Nociceptive responses to high and low rates of noxious cutaneous heating are mediated by different nociceptors in the rat: electrophysiological evidence. Pain 1996; 68: 141–150. DOI: 10.1016/S0304-3959(96)03177-6.

Response to referees

Reviewer #1 (Remarks to the Author):

The authors have successfully addressed my concerns. I have not more questions.

Response: We thank the reviewer for very valuable feedback.

Reviewer #2 (Remarks to the Author):

The authors have addressed all of our concerns. Congratulations on a beautiful and impactful study.

Response: We thank the reviewer for very valuable feedback.

Reviewer #3 (Remarks to the Author):

I co-reviewed this manuscript with one of the reviewers who provided the listed reports. This is part of the Nature Communications initiative to facilitate training in peer review and to provide appropriate recognition for Early Career Researchers who co-review manuscripts.

Reviewer #4 (Remarks to the Author):

The revision has markedly improved the paper.
However, I have still two major concerns:

1) The authors added data on a “peripheral nerve block of Abeta fiber” or “ Abeta-preferential block”:
Figure S7a suggests that there is a preferential block of A-beta fibers as compared to the less pronounced effect on A-delta cold fibers S7c. However, it appears that vibration ratings to the 200 Hz stimulus were reduced from 7 to 2 and cold detection thresholds worsened from around 3 degrees centigrade to 2.5 to 5.5°C. Thus, if a cold stimulus would have been 5.5°C colder that skin temperature, 50% of the volunteers would not have felt it (compared to 4 of 22 that gave a zero rating to the 200 Hz vibration stimulus. From my perspective, the test paradigms do not allow to draw firm conclusions on Abeta vs Adelta preference of the block.

It is important to note that the actual key experiment, i.e. the effect of an A-fiber block on pinprick-pain vs. cold and tactile sensation has already been published2. An A-fiber pressure block indeed blocks cold and tactile sensation while warmth detection is unchanged – however, the loss of first pain responses to pinprick – the very parameter that is mediated by A-fiber mechanonociceptors – showed exactly the same time course of the block as compared to the cold and tactile sensation. Thus, this direct evidence suggests that the time course of impaired A-beta and A-delta function following a pressure nerve block are both following the impaired first prinprick pain in humans-
[The absolute reaction time had a clear peak between 0.25 and 0.3 seconds – assuming a reaction time of 0.15s the remaining 0.1 to 0.15 s would be inline with conduction velocities of 10m/s i.e. in the Adelta range).

Response: We thank the reviewer for the thoughtful comment.

We agree that the ischemic pressure nerve block does not provide a complete separation between Aβ- and Aδ-fiber function. For this reason, throughout the manuscript we refer to this manipulation as an **Aβ-preferential**, rather than an Aβ-selective, nerve block.

The primary criterion for successfully established nerve block was the loss of tuning-fork sensation – this was accompanied by a retained ability to sense innocuous cooling, indicating profound impairment of Aβ-fiber function, while a substantial degree of Aδ-fiber function remained. The vibration rating tasks shown in Fig. S7b were included as complementary measures to further characterize the nerve block, whereas successful establishment of the block was determined primarily by loss of tuning-fork sensation (Fig. S7a, left), accompanied by a retained ability to sense innocuous cooling (>10-15°C); change in cold detection threshold (ΔCDT) relative to individual's skin temperature are shown in Fig. S7c. Taken together, these findings are consistent with a preferential (but not exclusive) effect on Aβ fibers.

We also agree with the reviewer that the cold sensitivity was reduced to some extent during the nerve block. To further address this point, we have now added new analyses (Fig. S7d-f), in which participants were stratified according to whether their ΔCDT exceeded −6.5°C, corresponding to the upper 95% confidence interval previously reported for the sole of the foot in healthy individuals (Magerl et al., PMID: 20965658). No differences were observed between these groups with respect to pain ratings or the ability to elicit withdrawal reflex.

Thus, variation in the degree of cold sensory impairment during the nerve block was not associated with differences in mechanically evoked pain perception or withdrawal reflexes, suggesting that the observed effects cannot be explained simply by greater impairment of Aδ afferents. Although this analysis cannot exclude a contribution of Aδ fibers, our observations are better explained by a dominant role for Aβ fibers.

We have added the following text to the Results:

"During the nerve block, cold sensitivity was reduced (Fig. S7c), indicating that cooling-sensitive Aδ fibers were affected in addition to Aβ fibers. In 9/22 individuals, the cold detection threshold (ΔCDT) exceeded −6.5°C, the upper 95 % confidence interval in the sole of the foot for healthy men and women[52]. However, no differences were observed with respect to pain ratings or the ability to elicit NWRs between these individuals and those that retained cold sensitivity within the normal range (Fig. S7d-f). Thus, variation in the degree of cold sensory impairment during the nerve block was not associated with differences in mechanically evoked pain perception or withdrawal reflexes, suggesting that the observed effects are not explained simply by greater impairment of Aδ afferents."

2) The differentiation between A-beta and A-delta fibers in humans Bhuiyan and Nagi acknowledge that thinly myelinated Adelta fibers are "rarely encountered" in human microneurography. (there have been speculations that the minute tip of the electrode need to be close a node of Ranvier in A-delta fibers to produce a sufficient signal and thus, minute movements will lead to a loss of the recording in contrast to C and A-beta fibers). In monkey, teased fiber techniques can be used that allow for stable recordings of A-delta fibers. These recording result in a clear bimodal distribution of A-fiber conduction velocity (A-delta peak between 10 and 15 m/s; A beta peak between 40 and 45m/s) 4. A corresponding bimodal distribution is also found in the diameter of myelinated axons in human sural nerve 1 (peaks around 4 and 8 μm) – thus, it appears that there is a lack of functional data on human A-delta fibers. Even though there is obvious variability of conduction velocities -for example, skin

temperature differences are a major source as seen by a lower conduction velocity of human A-fibers in the distal as compared to the proximal phalangeal segment with a difference of more than 10 m/s 3 – however, the clear bimodal distribution of axonal diameter in human sensory nerves and the clear bimodal distribution of conduction velocities in myelinated fibers in the monkey suggests that we do have a lack of information on single fiber function of A-delta fibers by microneurography.

In summary, I would suggest to downtown the claim, that (first) pin-prick pain is mediated by A-beta fibers and acknowledge that there is a certain lack of functional data on human A-delta fibers potentially linked to limitations of the microneurography recording technique as compared to teased fiber recordings.

[1] Dyck PJ, Karnes J, Sparks M, Low PA. The morphometric composition of myelinated fibres by nerve, level and species related to nerve microenvironment and ischaemia. Electroencephalogr.Clin Neurophysiol.Suppl. 1982; 36:39–55.

[2] Henrich F, Magerl W, Klein T, Greffrath W, Treede RD. Capsaicin-sensitive C- and A-fibre nociceptors control long-term potentiation-like pain amplification in humans. Brain. 2015; 138:2505–20.

[3] Mackel R. Conduction of neural impulses in human mechanoreceptive cutaneous afferents. J Physiol. 1988; 401:597–615.

[4] Treede RD, Meyer RA, Campbell JN. Myelinated mechanically insensitive afferents from monkey hairy skin: heat-response properties. J Neurophysiol. 1998; 80:1082–93.

Response: We thank the reviewer for this important comment.

We agree that there is presumably a bias against obtaining Aδ-fiber recordings in human microneurography likely owing to limitations of the microneurography technique, and that for this reason nociceptors conducting at Aδ velocities in humans may have been underrepresented in the published literature. To further clarify this in the manuscript, we have added the following text to the Supplementary Note 1:

"Nevertheless, it should be noted that few microneurography recordings of Aδ fibers have been published, and as morphological observations confirm the existence of an appreciable Aδ-fiber population in humans (e.g., Schellens et al. 1993), this may reflect technical limitations of microneurography compared with, for instance, teased nerve fiber recordings in non-human primates (Treede et al. 1998). Thus, the paucity of published microneurography recordings of human nociceptors conducting in the Aδ range does not preclude the existence of such fibers, and whereas convergent findings in the present study point to a dominant role for Aβ nociceptors in mechanical pain and reflexive behavior, other as yet unavailable approaches may be required to fully resolve this question."
